# The Clinical Translation of *α* -humulene – A Scoping Review

**DOI:** 10.1055/a-2307-8183

**Published:** 2024-05-08

**Authors:** Nishaanth Dalavaye, Martha Nicholas, Manaswini Pillai, Simon Erridge, Mikael H. Sodergren

**Affiliations:** 1 Medical Cannabis Research Group, Department of Surgery and Cancer, Imperial College London, UK; 2 Curaleaf Clinic, London, UK; 3 Curaleaf International, London, UK

**Keywords:** terpenes, anti-bacterial agents, *α* -humulene, *Humulus lupulus*, *Cannabis indica*, *Cannabis sativa*, Cannabaceae

## Abstract

*α* -humulene, a sesquiterpene found in essential oils of various plant species, has garnered interest due to its potential therapeutic applications. This scoping review aims to consolidate *α* -humuleneʼs evidence base, informing clinical translation, and guiding future research directions. A scoping review was conducted of EMBASE, MEDLINE, and PubMed databases up to 14th July 2023. All studies describing original research on *α* -humulene extraction, as well as pre-clinical and clinical research, were included for review. Three hundred and forty articles were analysed. *α* -humulene yields ranged from negligible to 60.90% across plant species. *In vitro* experiments demonstrated cytotoxicity against adenocarcinomas (such as colorectal, pulmonary, breast, prostatic, lung, and ovarian), with varying responses in other cell models. Mechanistic insights revealed its involvement in mitochondrial dysfunction, diminished intracellular glutathione levels, and the induction of oxidative stress. In rodent studies, oral administration of *α* -humulene at 50 mg/kg reduced inflammation markers in paw oedema and ovalbumin-induced airway inflammation. Intraperitoneal administration of *α* -humulene (50 – 200 mg/kg) exhibited cannabimimetic properties through cannabinoid 1 and adenosine A2a receptors. *α* -humulene also exhibited a multitude of properties with potential scope for therapeutic utilisation. However, there is a paucity of studies that have successfully translated this research into clinical populations with the associated disease. Potential barriers to clinical translation were identified, including yield variability, limited isolation studies, and challenges associated with terpene bioavailability. Consequently, rigorous pharmacokinetic studies and further mechanistic investigations are warranted to effectively uncover the potential of *α* -humulene.

## Introduction

Terpenoids are a vast and diverse group encompassing several classes of secondary metabolites from plants, each being investigated for biomedical properties 1 . Notably, they have been described as having anti-inflammatory, analgesic, antimicrobial, antioxidant, and estrogenic properties 2 , 3 . Sesquiterpenes are a class of terpene to which *α* -humulene (also known as *α* -caryophyllene) and its isomer *β* -caryophyllene belong. These sesquiterpenes share a three-isoprene unit structure, formed from the precursor farnesyl diphosphate, leading to the formation of cyclic or multi-ring compounds that contribute to their distinctive aroma 1 .

*α* -humulene and *β* -caryophyllene, though structurally similar, are differentiated by the open-ring structure present in *α* -humulene 4 . Historically, *α* -humulene was first identified as a major component in the essential oils of *Humulus lupulus* L., Cannabaceae, the common hop plant, from which it derived its name. Its structural elucidation was achieved through nuclear magnetic resonance spectroscopy. Furthermore, *α* -humulene has not only been sourced from *Humulus lupulus* but also from *Cannabis Indica* L., Cannabaceae, signifying its prevalence in botanical species for which there are already well-established agricultural processes 5 . The isolation and extraction of *α* -humulene from its botanical sources have been refined over time. Modern techniques, such as steam distillation, are employed to capture the volatile essential oils containing this sesquiterpene. Its abundance in various botanical sources makes it a subject of interest for both traditional and modern medicinal applications.

There is continual demand to identify novel compounds that possess anticancer, anti-inflammatory, and antimicrobial properties, in light of a persisting cancer burden, rising incidence of inflammatory conditions, and emergence of antimicrobial resistance 6 , 7 , 8 . Despite promising preclinical evidence supporting the multimodal therapeutic potential of ***α*** -humulene, there are several barriers to its clinical translation. Whilst certain plant species are known to be rich sources of ***α*** -humulene, plant yields are often reported as being low, and so far, researchers have not reached a consensus on a named plant species that consistently yields high concentrations of *α* -humulene. At present, biosynthesis pathways are therefore being explored as an avenue to create synthetic *α* -humulene to overcome inherent challenges with the manufacturing of compounds that are reliant upon favourable agricultural conditions 9 . In addition, much of the research conducted to date has utilised a combination of organic compounds contained within the plant essential oil, rather than assessing the properties of *α* -humulene in isolation, resulting in a paucity of evidence summarising *α* -humuleneʼs individual properties. It is therefore important to evaluate studies that report specifically the properties of *α* -humulene and identify species with acceptable yields in order to advance this scientific field.

A systematic review by Leite et al. 10 summarised the preclinical properties of sesquiterpene compounds, including *α* -humulene and *β* -caryophyllene. Whilst preclinical evidence has been promising regarding the properties of ***α*** -humulene, there has been minimal progress into clinical translation of this research. Hence, this review aimed to provide a synthesised evidence base for the prioritisation of future research, including optimisation of agriculture and manufacturing, alongside identification of the most promising biomedical applications.

## Results

### Search results

**Fig. 1:**
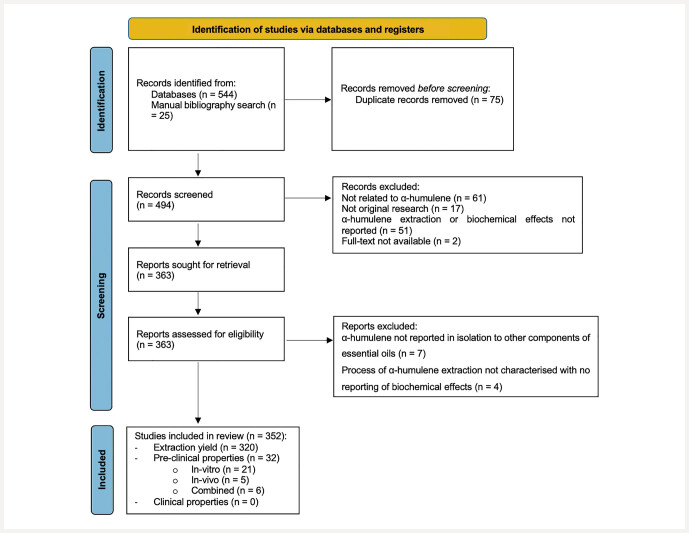
PRISMA flow chart showing the process of inclusion and exclusion of patients for analysis in this scoping review.

The database and manual bibliography search initially returned 544 studies ( Fig. 1 ). Four hundred and twelve full-text articles were assessed for eligibility, with 340 articles included for qualitative synthesis. Three hundred and seven studies included reported the extraction yields of *α* -humulene (Supplementary Material A). Thirty-two studies 11 , 12 , 13 , 14 , 15 , 16 , 17 , 18 , 19 , 20 , 21 , 22 , 23 , 24 , 25 , 26 , 27 , 28 , 29 , 30 , 31 , 32 , 33 , 34 , 35 , 36 , 37 , 38 , 39 , 40 , 41 , 42 were included for evaluation of the pre-clinical properties of *α* -humulene. These included investigations conducted *in vitro* 11 , 12 , 13 , 14 , 15 , 16 , 17 , 18 , 19 , 20 , 21 , 22 , 23 , 24 , 25 , 26 , 27 , 28 , 29 , 30 , *in vivo* 31 , 32 , 33 , 34 , 35 , 36 , and combined *in vitro* and *in vivo* experiments 37 , 38 , 39 , 40 , 41 , 42 . Notably, no studies were found to assess the clinical properties of *α* -humulene.

### Yield of *α* -humulene from extraction

Yields of *α* -humulene were reported from 462 different plant and animal species (Supplementary Material B). Reported yields varied from nil to 60.90%. Table 1 highlights the five species that exhibited the highest reported yields among the included studies. The most common method of *α* -humulene extraction was hydrodistillation in a Clevenger-type apparatus. Concurrently, isolation most frequently relied on gas chromatography–mass spectrometry (GC-MS). Among the species analysed, *Lantana camara* L., Verbenaceae; *Origanum majorana* L., Lamiaceae; *Cordia verbenacea* DC., Boraginaceae; *Cannabis sativa;* and *Daucus carota* L., Apiaceae were prominent contributors, with the greatest number of studies reporting *α* -humulene extraction data.

**Table:** **Table 1** Summarising the species with the top five highest reported yields of *α* -humulene.

Species	Chemovar	Extraction	Isolation	Yield	Reference
*Aframomum melegueta* [alligator pepper]	Nigeria	Hydrodistillation for 3 h	Fractionation	60.90%	Ajaiyeoba E et al. 1999 77
*Leptospermum sp.* [Mt Maroon A. R. Bean 6665]	Australia	Hydrodistillation with incubation	GC-MS	44.00 – 51.00%	Brophy J et al. 2000 78
*Humulus lupulus* . [Chinook variety]	Brazil	Hydrodistillation using a Clevenger-type apparatus	GC-MS	31.50 – 34.62%	Duarte et al. 2023 79
*Camponotus japonicus* [insect]	Japan	Macerated in 10 mL of pentane	GC-MS	35.80%	Sakurai K et al. 2020 80
*Zingiber nimmonii*	India	Hydrodistillation using a Clevenger-type apparatus for 8 h	GC-MS	19.60%	Govindarajan et al. 2016 81
GC-MS – gas chromatography–mass spectrometry					

### Specific properties of *α* -humulene

#### Antiproliferative properties

Thirteen studies evaluated the effects of *α* -humulene in cancer models ( Table 2 ) 11 , 12 , 13 , 14 , 15 , 16 , 17 , 18 , 19 , 20 , 31 , 37 , 38 . Across these studies, *α* -humulene consistently demonstrated cytotoxic activity against tumour cells, with one exception by Loizzo et al. (2008) 20 involving human amelanotic melanoma (C32) and renal cell adenocarcinoma (ACHN) at a concentration of 9.3 × 10 ^−7^ to 1.2 × 10 ^−4^ . *α* -humulene, sourced from *Myrica rubra* Siebold & Zucc., Myricaceae, has demonstrated substantial anti-proliferative effects on colorectal cancer cell lines *in vitro* , marked by mitochondrial membrane potential disruption and enhanced efficacy when combined with conventional anticancer drugs 11 . In hepatocellular carcinoma (HCC), *α* -humulene from *Eupatorium odoratum* L., Asteraceae exhibited selective inhibition of HCC cell proliferation primarily *in vitro* , associated with the suppression of protein kinase B signalling 37 . Notably, *α* -humulene demonstrated dose-dependent inhibition of ovarian and lymphoblast cancer cell proliferation *in vitro* and synergistic effects with doxorubicin 12 . Its preferential cytotoxicity towards tumour cells, sparing non-tumour cells, indicates potential selectivity for actively dividing cancer cells. This anti-proliferative activity has also been related to apoptosis induction and modulation of reactive oxygen species (ROS) production 37 . In an *in vivo* study on clove terpenes, *α* -humulene induced significant glutathione S-transferase activity in mouse liver and small intestine tissues, suggesting a role in detoxification processes 31 . Fukuoka et al. (2004) 21 showed *α* -humuleneʼs antiproliferative properties in rat arterial smooth muscle cells, utilising a cell assay that induced proliferation with heat shock protein. The study reported an IC50 value of 0.122 µM, showcasing dose-dependent effects and superior potency compared to its analogue zerumbone. Inhibitory effects were demonstrated even at a concentration of 4.89 × 10 ^− 6^ mol/L.

**Table:** **Table 2** Summary of studies investigating the anticancer properties of isolated *α* -humulene.

Model	Concentration/Dose	Results	Reference
*In vitro* colorectal adenocarcinoma epithelial cells [CaCo-2 and SW-620]	5 × 10 ^−5^ , 1 × 10 ^−4^ , and 1.5 × 10 ^−4^ M	*α* -humulene exhibited antiproliferative activity in combination with oxaliplatin and 5-fluorouracil at 100 and 150 µmol/L due to decreased mitochondrial membrane potenzial.	Ambroz et al. 2019 11
*In vitro* hepatocellular carcinoma cells [huh7, SMMC-7721, HepG2 and Hep3B] and *in vivo* HepG2-bearing nude mice	*In vitro* : 6.1 × 10 ^−6^ – 2.4 × 10 ^−4^ M *In vivo* : 10 and 20 mg/kg	*In vitro, α* -humulene inhibited proliferation of all hepatocellular carcinoma cell lines at 15 µmol/L, inducing cytotoxicity via intrinsic apoptotic pathways. Similar findings were reported *in vivo* at 10 mg/kg.	Chen et al. 2019 37
*In vitro* ovarian cancer cells [A2780 and SKOV3 and lymphoblasts CCRF/CEM and CEM/ADR]	20, 40, 100, and 200 µM	*α* -humulene showed antiproliferative activity against certain ovarian cancer cell lines [A2780 at 40 µM, SKOV3 at 200 µM] and lymphoblast cell lines [CCRF/CEM at 200 µM, no effect on CEM/ADR at 200 µM].	Ambroz et al. 2017 12
*In vitro* colon adenocarcinoma [CaCo-2] and non-cancer cells [rat hepatocytes]	0 – 2.4 × 10 ^−4^ M	*α* -humulene demonstrated antiproliferative activity against cancer cells at 4.9 × 10 ^−5^ mol/L, with an IC50 of 24.4 ± 2.4. Additionally, *α* -humulene potentiated doxorubicinʼs anticancer properties in cancer cells, while showing no effect on non-cancer cell viability.	Ambroz et al. 2015 13
*In vitro* mice melanoma, human hepatocellular carcinoma, chronic human myelocytic leukaemia, and human promyelocytic leukaemia	9.3 × 10 ^−7^ – 1.2 × 10 ^−4^ M	No significant anticancer activity of *α* -humulene was identified for any concentration tested.	Costa et al. 2015 38
*In vitro* : colon human cancer [HT-29], human hepatocellular carcinoma [J5], and human pulmonary adenocarcinoma [A549]	0 – 9.8 × 10 ^−4^ M	*α* -humulene exhibited significant cytotoxicity against all cell lines, with IC50 values of 5.2 × 10 ^−5^ , 1.8 × 10 ^−4^ , and 1.3 × 10 ^−4^ mol/L for HT-29, J5, and A549, respectively.	Su et al. 2015 14
*In vitro* : human colorectal. adenocarcinoma [HCT-116], human breast cancer [MCF-7] and murine macrophages [RAW264.7]	7.6 × 10 ^−6^ – 4.9 × 10 ^−4^ M	*α* -humulene demonstrated cytotoxic potenzial by inhibiting cancer cell growth, with IC50 values of 3.1 × 10 ^−4^ , 4.2 × 10 ^−4^ , and 1.9 × 10 ^−4^ mol/L for HCT-116, MCF-7, and RAW264.7 cell lines, respectively.	Hadri et al. 2010 15
Murine small bowel mucosa and liver	9.8 × 10 ^−5^ M	*α* -humulene showed potenzial inhibitory action against carcinogenesis by increasing glutathione S-transferase [GST] activity. The enzyme activity increased by 99% in the liver and 152% in the small bowel.	Zheng et al. 1992 31
Cell lines of human breast adenocarcinoma [MCF-7], prostatic adenocarcinoma [PC-3], lung carcinoma [A-549], colon adenocarcinoma and fibroblasts [DLD-1 e L-929]	2.4 × 10 ^−4^ and 9.8 × 10 ^−4^ M	*α* -humulene caused dose-dependent glutathione depletion of 38% and 71% at 50 and 200 µM, respectively, along with increased production of reactive oxygen species by 163% and 278% after 1 and 4 hours. Normal human fibroblasts showed lower cytotoxic effects.	Legault et al. 2003 16
Cell lines of human breast adenocarcinoma [MCF-7], colon adenocarcinoma [DLD-1: ATCC # CCL-221], murine fibroblasts [L-929 ATCC # CCL-1]	7.8 × 10 ^−5^ – 3.1 × 10 ^−4^ M	*α* -humulene showed cytotoxicity at 1.6 × 10 ^−4^ and 3.1 × 10 ^−4^ mol/L. Cell growth inhibition by *α* -humulene was significantly increased from 50 ± 6% alone to 75 ± 6% by co-administration of non-cytotoxic levels [10 µg/mL] of caryophyllene, potenzially due to altered membrane permeability.	Legault et al. 2010 17
Cell lines of human breast adenocarcinoma [MCF-7 and MDA-MB-468], human malignant melanoma: [UACC-257]	Concentration not reported	*α* -humulene from *Eugenia zuchowskiae* inhibited all cell lines, with similar cytotoxicity against MCF-7 line as doxorubicin [LC50 of 1.1 × 10 ^−4^ and 1.4 × 10 ^−4^ mol/L, respectively].	Cole et al. 2007 18
Cell lines of human cervical carcinoma [HeLa], human colon adenocarcinoma [HT-29], monkey kidney [Vero]	9.8 × 10 ^−7^ – 9.8 × 10 ^−4^ M	*α* -humulene demonstrated cytotoxicity against all cell lines. Tumour cell lines were more sensitive to cytotoxic activity than non-tumour Vero cells and murine macrophages.	Silva et al. 2008 19
Cell lines of human amelanotic melanoma [C32], renal cell adenocarcinoma [ACHN]	up to 4.9 × 10 ^−4^ M	*α* -humulene did not demonstrate cytotoxicity with IC50 > 4.9 × 10 ^−4^ mol/L against both C32 and ACHN lines. However, *β* -caryophyllene showed cytotoxic activity against both.	Loizzo et al. 2008 20
IC50 – half maximal inhibitory concentration; LC50 – half maximal lethal concentration			

#### Anti-inflammatory properties

Exploration into the *in vivo* anti-inflammatory properties of *α* -humulene, isolated from *Cordia verbenacea* , has yielded significant insights as well. Passos et al. (2007) 39 showed the potent anti-inflammatory attributes of *α* -humulene by demonstrating its ability to significantly inhibit carrageenan-induced paw oedema in murine models and by a notable reduction in tumour necrosis factor (TNF)- *α* levels in response to carrageenan. Fernandes et al. (2007) 32 conducted a similar evaluation using oral administration of *α* -humulene against several experimental murine and rat models. Notably, administration of *α* -humulene at 50 mg/kg demonstrated a dose-dependent reduction in paw oedema, indicating its efficacy in mitigating the acute phase of inflammation. Additionally, it exhibited a sustained anti-inflammatory effect by inhibiting the late phase of carrageenan-induced oedema. Mechanistically, *α* -humulene interfered with multiple pathways involved in inflammation, including the inhibition of bradykinin, a platelet-activating factor, and histamine-induced oedema. Basting et al. (2019) 33 also observed a reduction in carrageenan-induced paw oedema. Despite not significantly affecting neutrophil migration, *α* -humulene suppressed the release of TNF- *α* and interleukin (IL)-1 *β* and inhibited prostaglandin E2 production. Similar findings were observed by Medeiros et al. (2007) 34 in lipopolysaccharide-induced rat paw oedema. Key observations included a reduction in pro-inflammatory cytokines, inhibition of kinin B1 receptor upregulation, and suppression of neutrophil recruitment by targeting nuclear factor-kappa B (NF- *κ* B) activation. Notably, *α* -humuleneʼs efficacy surpassed that of trans-caryophyllene.

In a murine model of airway allergic inflammation, female BALB/c mice challenged with ovalbumin experienced a significant reduction in eosinophil recruitment to bronchoalveolar lavage fluid and lung tissue when administered *α* -humulene preventively or therapeutically 35 . *α* -humulene exhibited modulation of critical asthma-related mediators, including IL-5, C-C motif chemokine11, and leukotriene B4, along with the inhibition of P-selectin expression, a crucial factor in eosinophil migration. Additionally, *α* -humulene showed inhibitory effects on NF- *κ* B and activator protein-1. Histological analysis indicated a decrease in mucus hypersecretion, suggesting a potential role in balancing T-helper cell responses.

Contrary to the widely positive findings reported regarding the anti-inflammatory activity of *α* -humulene, Viveiro et al. (2022) 22 investigated pterygium fibroblasts through *in vitro* exposure experiments. Third-passage pterygium fibroblasts were subjected to *α* -humulene concentrations (0.25, 2.5, and 25 µmol/L), and the subsequent cell viability assay revealed no significant cytotoxicity and minimal variation in inflammatory markers. This highlights the importance of considering cell-type-specific responses and experimental conditions in evaluating potential therapeutic benefits.

#### Antimicrobial properties

Early exploration into the *in vitro* antimicrobial potential of *α* -humulene was undertaken by Pichette et al. (2006) 23 who observed antibacterial activity against *Staphylococcus aureus* at a mean inhibitory concentration (MIC) of 1.3 × 10 ^−5^ mol/L. Subsequent investigations by Azizan et al. (2017) 24 expanded on this by demonstrating the dose-dependent bacteriostatic and bactericidal effects of *α* -humulene. Employing the broth microdilution method and *α* -humulene sourced from *Orthosiphon stamineus* and *Ficus deltoidei* , the study showed moderate to strong inhibition across a range of bacteria. Notably, oral Gram-negative species ( *Porphyromonas gingivalis, Fusobacterium nucleatum* , and *Aggregatibacter actinomycetemcomitans* ) exhibited greater susceptibility compared to Gram-positive bacteria ( *Enterococcus faecalis, Streptococcus mutans, Streptococcus mitis* , and *Streptococcus salivarius* ). Mechanistically, electron microscopy revealed morphological alterations, indicating that *α* -humulene interfered with the membrane structure or cell wall of oral bacteria. This effect was ascribed to the substantial electronegativity resulting from the carbon double bond configurations in its molecular structure.

Jang et al. (2020) 25 evaluated the *in vitro* antibacterial and antibiofilm effects of *α* -humulene extracted from *Bacteroides fragilis* . The study determined the MIC for cell growth and biofilm formation to be 9.8 × 10 ^−6^ mol/L. Through qRT-PCR analysis, concentration-dependent reductions in the expression of *bmeB1* and *bmeB3* genes were observed in various *Bacteroides fragilis* strains. This indicated increased antibiofilm action given these genes are implicated in the development of the biofilm matrix and antibiotic resistance. Notably, there was a marked reduction in cellular metabolic activity at concentrations of 3.9 × 10 ^−5^ to 7.8 × 10 ^−5^ mol/L. Moreover, confocal laser scanning microscopy revealed that *α* -humulene not only diminished cell density and thickness but also effectively reduced protein, carbohydrate, and nucleic acid levels. Rossato et al. (2022) 26 evaluated *α* -humuleneʼs antibacterial potential against *Staphylococcus aureus* and *Enterococcus faecalis* using experimental light-cured periodontal dressing formulations and the modified direct contact model. Formulations with 10% and 20% *α* -humulene reduced bacterial growth after 1 and 24 hours of incubation compared to the control group, indicating sustained antibacterial activity.

Xing et al. (2018) 40 focused on evaluating the antifungal properties of humulene. Findings revealed a dose-dependent impediment of *Peronophythora litchii* growth, with scanning and transmission electron microscopy uncovering discernible morphological and ultrastructural changes. *In vivo* evaluations on litchi foliage and fruits demonstrated a notable reduction in disease severity. *α* -humulene exhibited weak inhibitory effects against *Peronophythora litchi* at high concentrations (8.7 × 10 ^−4^ to 4.4 × 10 ^−3^ mol/L) 40 .

#### Antiallergic properties

The antiallergic potential of *α* -humulene was demonstrated by Tanaka et al. (1996) 36 in the context of treatment for atopic conditions. Using a sensitised murine model of passive cutaneous anaphylaxis, *α* -humulene administration prior to antigen challenge demonstrated dose-dependent inhibition of allergic reactions at 20, 40, and 80 mg/kg, with approximately four times the potency of the reference drug tranilast. However, the observed effects were less potent than the antiallergy effects triggered by *β* -caryophyllene. The study suggested the bicyclic ring structure inherent in *β* -caryophyllene may have contributed to the enhanced antiallergy activity of the compound. Additionally, Fernandes et al. (2007) 32 demonstrated *α* -humulene reduced paw oedema in sensitised mice challenged with ovoalbumin, suggesting its anti-inflammatory properties in alleviating allergic responses.

#### Antiparasitic properties

De Oliveria et al. (2017) 27 evaluated the antischistosomal effects of *α* -humulene against *Schistosoma mansoni* following *in vitro* exposure. At concentrations of 1 mol/L, *α* -humulene exhibited notable efficacy, causing mortality rates of 60% for female worms and 80% for male worms after a 72-hour incubation period. The sesquiterpene also induced a substantial reduction in motor activity and oviposition across all concentrations, highlighting its potential as a promising antiparasitic agent. Additionally, there were significant inhibitory effects of *α* -humulene on the excretory system of male *Schistosoma mansoni* adult worms. However, this inhibitory activity was not observed in female worms. The mechanism underlying this was attributed to the inhibition of the expression of P-glycoprotein, a product of the multidrug resistance 2 gene, within the excretory system of male *Schistosoma mansoni* worms. The study further employed Hoechst probe and scanning electron microscopy to assess the impact of *α* -humulene on the membrane integrity of *Schistosoma mansoni* . This highlighted the substantial damage caused to the tegument by *α* -humulene exposure.

#### Gastroprotective properties

Lemos et al. (2015) 41 investigated the potential gastroprotective effects of *α* -humulene through their study involving murine gastric ulcer models The researchers administered an oral dose of 30 mg/kg of isolated *α* -humulene, equivalent to omeprazole dosing. This dose led to a substantial reduction of 76.20% in gastric lesions induced by 0.2 ml of an ethanol/hydrogen chloride solution (60%/0.3 M). This investigation revealed two significant mechanisms contributing to the gastroprotective effects: a reduction in gastric acid secretion and an increase in mucus production.

#### Larvicidal properties

The larvicidal potential of *α* -humulene was examined by Govindarajan et al. (2016) 28 in an *in vivo* study encompassing three vector species: *Anopheles subpictus* Grassi (Culicidae) *; Aedes albopictus* Skuse (Culicidae); and *Culex tritaeniorhynchus* Giles (Culicidae). The researchers determined the lethal concentration 50 (LC50) values as 3.0 × 10 ^−5^ , 3.4 × 10 ^−5^ , and 3.6 × 10 ^−5^ mol/L for the respective species. The impact on non-target species was notably less, with a significantly lower LC50 of 5.0 × 10 ^−3^ mol/L observed in *Gambusia affinis* fish. Furthermore, there were no adverse effects on fish survival or swimming activity following the administration of *α* -humulene concentrations approaching the calculated larvae LC90.

Hung et al. (2021) 29 also evaluated the larvicidal effects of *α* -humulene from the essential oil of *Lantana camara* . It showed promising larvicidal activities against important mosquito vectors, with 48-hour LC50 values of 1.9 × 10 ^−4^ mol/L for *Anopheles aegypti* L. (Culicidae), 1.9 × 10 ^−4^ mol/L for *Aedes albopictus* , and 4.3 × 10 ^−4^ mol/L for *Culex quinquefasciatus* Say (Culicidae). Additionally, *α* -humulene exhibited notable mosquito larvicidal effects with an inhibitory concentration (IC50) value of 7.9 × 10 ^−4^ against electric eel acetylcholinesterase. Furthermore, *in silico* studies have demonstrated *α* -humulene exhibits significant binding energy in docking studies targeting sterol carrier protein-2, indicating its potential as an effective antilarvicidal agent 30 .

#### Molluscicidal properties

In the context of molluscs acting as intermediate hosts for several helminths, *α* -humuleneʼs potential molluscicidal properties have been subjected to scrutiny 29 . Notably, its LC50 values at 24 hours have been reported to be 9.3 × 10 ^− 5^ mol/L, 9.3 × 10 ^− 5^ mol/L, and 9.1 × 10 ^− 5^ mol/L for *Pomacea canaliculate* (Lam.), Ampullariidae; *Gyraulus convexiusculus* (Hutton), Planorbidae; and *Tarebia granifera* (Lam.), Thiaridae, respectively.

#### Cannabimimetic properties

The cannabimimetic properties of *α* -humulene were demonstrated by LaVigne et al. (2021) 42 through *in vivo* and *in vitro* experiments. Using various behavioural assays in mice, *α* -humulene manifested notable antinociceptive effects. The study identified specific receptor targets influenced by *α* -humulene, revealing interactions with cannabinoid type 1 (CB1) and type 2 (CB2) receptors, as well as adenosine receptor A2a, through *in vitro* experiments. Furthermore, *in vivo* experiments demonstrated a synergistic interaction between *α* -humulene and the synthetic cannabinoid agonist WIN55,212 – 2, leading to enhanced antinociceptive effects. There were selective effects of *α* -humulene on various behaviours associated with the tetrad, emphasising its complex interplay with multiple receptor systems. Notably, the *in vitro* studies showed the CB1-dependent nature of *α* -humulene activation, requiring relatively high concentrations for receptor activation, a phenomenon reversible by the CB1 antagonist rimonabant.

## Discussion

The scoping review undertaken in this study unveils the landscape of *α* -humuleneʼs pharmacological potential, revealing a diverse spectrum of documented properties across various studies. These encompass anti-inflammatory, antimicrobial, antiproliferative, antiallergic, gastroprotective, and even cannabimimetic effects. *α* -humulene interacts with diverse biological pathways, suggesting its potential for addressing various health conditions.

The review further emphasises the pivotal role played by specific species that yield substantial amounts of *α* -humulene, carrying profound implications for pharmaceutical applications. Noteworthy among these is *Aframomum melegueta* , a West African spice renowned for its historical medicinal uses and significant *α* -humulene content, rendering it an enticing candidate for therapeutic extraction 43 . Likewise, several *Leptospermum* species, known for their potent antimicrobial properties, exhibit notable levels of *α* -humulene 44 . Additionally, *Humulus lupulus* , commonly known as hops, has a high *α* -humulene content. Given its well-documented applications and extensively studied properties, hops offer a versatile avenue for the development of *α* -humulene-based therapeutics 45 . Another plant of significance is *Cannabis sativa* , in which *α* -humulene is already utilised in full-spectrum cannabis-based medicinal products. The cannabimimetic effects of *α* -humulene may give rise to potential additive or synergistic effects when administered alongside cannabinoids and other active pharmaceutical ingredients, broadly referred to as ʼthe entourage effectʼ 46 . Collectively, these species enrich the available sources of *α* -humulene, highlighting its prevalence across a diverse range of botanicals. These species hold promise as potential sources for pharmaceutical extraction due to their abundant *α* -humulene content. By harnessing extracts derived from these species, either in combination with other compounds or as standalone treatments, further exploration of potential solutions for various health conditions becomes viable.

The translation of promising preclinical findings to clinical practice encounters barriers. Variability in *α* -humulene yield across different botanical sources poses logistical challenges for large-scale extraction 47 . The limited exploration of isolated *α* -humulene outside of whole essential oils highlights the importance of comprehensive investigations into isolated properties 48 . Addressing this inconsistency requires the identification of further plant species with high *α* -humulene yields or increased investigation of biosynthesis pathways for synthetic production.

The potential of *α* -humulene as an anticancer agent is particularly promising. Studies have established wide-ranging effects on various cancer cell lines, revealing nuanced interactions with distinct tumour types. This is coherent with preclinical studies on other terpenes, which similarly find anticancer potential 49 . The mechanism of action of *α* -humulene appears multifactorial, including increasing the production of reactive oxidative species and induction of apoptosis 16 , 37 . Moreover, *α* -humulene was associated with glutathione depletion, which makes cancer cells more sensitive to stress caused by reactive oxidative species 16 . *α* -humulene was associated with an increase in GST activity, which is also seen with other terpenes 50 . This feature, however, is typically associated with improved cancer cell survival and resistance to certain chemotherapeutics due to the associated efflux of anticancer agents from the cell 51 . Putra et al. investigated *α* -humuleneʼs interaction with the overexpressed HER-2 protein using docking methods and shed light on its potential as an anti-breast-cancer agent. The *in silico* molecular docking simulations reveal a binding energy of − 7.50 kcal/mol, affirming its efficacy against breast cancer 52 . As such, its effects within human studies are eagerly awaited, especially as preclinical studies show that *α* -humulene may have synergistic effects with doxorubicin and other chemotherapeutics 13 . This is particularly important as present studies indicate that *α* -humulene would not be a suitable chemotherapeutic agent in isolation and would otherwise be best used alongside traditional chemotherapeutics 53 . Its lower toxicity profile also supports this as a potential future use, provided efficacy can be determined 54 . Further mechanistic studies, including investigations into synergistic interactions with established chemotherapeutics, are ultimately necessary to fully leverage *α* -humuleneʼs potential in cancer biology 55 .

Beyond its cancer-related properties, *α* -humulene is a compelling anti-inflammatory agent. Its modulation of the NF- *κ* B pathway and subsequent suppression of key inflammatory mediators demonstrates its potential in various inflammatory conditions 56 . Insights gained from murine models of asthma highlight its immunomodulatory potential, positioning *α* -humulene as a contender for treating inflammatory and atopic conditions 35 . Additionally, its analgesic potential and observed gastroprotective effects hold significance 57 . In contrast to traditional non-steroidal anti-inflammatory drugs, notorious for causing peptic ulcer disease and adverse renal effects, *α* -humulene offers a potentially safer alternative for managing inflammation-associated conditions 58 .

The pharmacodynamic profile of *α* -humulene indicates its capability of addressing various aspects of health and disease. Its interactions with different molecular pathways suggest complex biochemical dialogues within cells and tissues. This complexity is particularly relevant for multifactorial conditions like cancer and chronic inflammatory diseases, where several dysregulated pathways contribute to aetiology and pathogenesis 59 , 60 . By targeting these pathways, *α* -humulene introduces a novel therapeutic approach distinct from the traditional ʼone-target-one-drugʼ paradigm 61 , 62 .

The antimicrobial properties of *α* -humulene enrich its pharmacological profile, spanning antibacterial, antiparasitic, and antifungal effects. Its efficacy in restraining biofilm formation and curtailing gene expression associated with biofilm matrix development and antibiotic resistance is particularly relevant given the growing appreciation for the role of biofilm in antibiotic resistance 63 , 64 . This enhances the potential to address antibiotic-resistant infections, a pressing global health concern 65 .

The paucity of clinical studies involving *α* -humulene necessitates thorough evaluation in the clinical setting to validate its efficacy, safety, and optimal dosage regimens in human subjects before widespread use 66 . In addition, it is crucial to emphasise the necessity of pharmacokinetic studies, particularly for terpenes like *α* -humulene. Due to their lipophilic nature, terpenes often exhibit poor water solubility and are susceptible to rapid metabolism and elimination, leading to low oral bioavailability 2 , 67 . Therefore, rigorous pharmacokinetic studies in animals and humans are essential to optimise dosing strategies to understand *α* -humuleneʼs therapeutic potential 68 . Strategies to enhance *α* -humuleneʼs bioavailability, such as formulations that improve solubility and stability, could significantly enhance its clinical utility 69 . There is one pilot study currently underway seeking to explore the effects of *α* -humulene on stress when combined with forest bathing, for which the results will be eagerly awaited 70 .

Acknowledging the limitations of this scoping review is vital. The heterogeneity of study methodologies, including variations in cell lines, experimental conditions, and assessment methods, poses a challenge in directly comparing the results. This heterogeneity limits the ability to perform quantitative meta-analyses and emphasises the need for cautious interpretation. Variations in study design, quality, sample size, and reporting practices could impact the overall strength of evidence. The limited number of *in vivo* studies and the absence of clinical trials restrict the ability to directly extrapolate findings to human populations to provide clinical validation 71 . Preclinical studies often involve isolated cells or animal models, which may not fully replicate human physiological responses 72 . Furthermore, it is important to acknowledge the limitations associated with the compilation of *α* -humulene yield data from various locations, with a focus solely on the highest reported yields. This approach might not account for potential variations in cultivation practices, environmental factors, and genetic influences that can significantly affect yield outcomes. Relying solely on the highest reported yields could lead to an incomplete understanding of the compoundʼs availability and potentially skew the representation of humulene yields. Moreover, studies often failed to specify whether the *α* -humulene yield was from the whole plant, flower, or another plant component. This lack of clarity restricts the interpretation, as there can be substantial variation in terpene content across various flower structures 73 .

Overall, this systematic review provides valuable insights into *α* -humuleneʼs potential therapeutic properties. However, addressing limitations through standardised methodologies, clinical trials, and consistent reporting practices is crucial for an accurate understanding of its multifaceted effects and clinical applications. The future of *α* -humuleneʼs clinical translation hinges on collaborative efforts, pharmacokinetic evaluations, rigorous clinical trials, innovative formulation strategies, and partnerships across disciplines. Through these efforts, *α* -humuleneʼs clinical translation can be accelerated in light of its many promising therapeutic properties.

## Materials and Methods

A scoping review, utilising methods outlined by Arksey and OʼMalley and Levac et al. 74 , 75 , was conducted of the current literature on *α* -humulene.

### Research question

This scoping review focused on identifying and clarifying key research aspects and characteristics of the available literature with regards to *α* -humulene. Given its potential therapeutic effects in the clinical setting, this review evaluated the evidence base for *α* -humulene in terms of its extraction and properties that may be translated for medicinal purposes. This review also aimed to identify any specific gaps in the evidence base that may inform the work of future researchers in this area of sesquiterpene research.

### Data sources and search strategy

A broad search was conducted of MEDLINE, PubMed, and EMBASE databases in accordance with the Preferred Reporting Items for Systematic Reviews and Meta-Analyses (PRISMA) guidelines 76 . A search was conducted from 1946 to July 14, 2023, utilising the search terms ʼhumuleneʼ, ‘alpha-humuleneʼ, and ʼalpha-caryophylleneʼ with the Boolean operator ʼOR′ ( **Table S1** , Supporting Information). The literature search was conducted by three independent researchers. For any discrepancies identified, a senior author planned to review these if necessary. Additional relevant articles were included through manual search of the bibliographies of included studies. Articles were screened in relation to the topic area and included if deemed to meet the inclusion criteria. The precise search strategies performed can be found in Supplementary Material C.

### Study selection criteria

Inclusion criteria consisted of original articles related to *α* -humulene that included extraction, pre-clinical, and clinical research. Studies were excluded if they did not constitute original primary research or the outcomes of *α* -humulene were not reported in isolation from other essential oils extracted from plant species.

### Data extraction and presentation

Data extraction was performed independently by three authors. If outcomes were not reported within the published article, but described within the methodology, corresponding authors were contacted for additional information. Concentrations are presented as percentage yield or micromolar concentrations (µM) with the standard deviation (S. D.), standard error (S. E.), or range if reported.

## Contributorsʼ Statement

Data collection: N. Dalavaye, M. Nicholas, M. Pillai; Design of the study: N. Dalavaye, M. Nicholas, M. Pillai, S. Erridge, M. H.Sodergren; Statistical analysis: N. Dalavaye, M. Nicholas, M. Pillai, S. Erridge; analysis and interpretation of the data: N. Dalavaye, M. Nicholas, M. Pillai, S. Erridge, M. H.Sodergren; drafting the manuscript: N. Dalavaye, M. Nicholas, M. Pillai, S. Erridge; critical revision of the manuscript: N. Dalavaye, M. Nicholas, M. Pillai, S. Erridge, M. H.Sodergren

## Supporting Information

Supplementary Material A: References for studies of extraction yields for different species
Supplementary Material B: Reported yields of *α* -humulene
Supplementary Material C: Search strategy Ergänzendes Material Supporting Information
