# Supplementary material for: The Clinical Translation of α -humulene – A Scoping Review
Source: Planta Med. 2024 May 8;90(9):664–74. doi: 10.1055/a-2307-8183 (PMC11254484; doi:10.1055/a-2307-8183)
Supplement: Supplementary file 1 — Ergänzendes Material [file 10-1055-a-2307-8183-sup_pmh0706.pdf]

Supplementary material

Supplementary Material A
Included studies reporting extraction yields of α-humulene

1. Abreu IN, Reis MG, Marsaioli AJ, Mazzafera P. Essential oil composition of *Hypericum brasiliense* choise. Flavour and Fragrance Journal. 2004;19(1):80–2.

2. Adamenko K, Kawa-Rygielska J. Effect of Hop Varieties and Forms in the Hopping Process on Non-Alcoholic Beer Quality. Molecules. 2022 Nov 16;27(22):7910.

3. Agnaniet H, Menut C, Bessiere JM. Aromatic plants of tropical central Africa. Part XLIX+: Chemical composition of essential oils of the leaf and rhizome of *Aframomum giganteum* K. Schum from Gabon. Flavour and Fragrance Journal. 2004;19(3):205–9.

4. bin Ahmad F, bin Jantan I. The essential oils of *Boesenbergia stenophylla* R. M. Sm. as natural sources of methyl (E)-cinnamate. Flavour and Fragrance Journal. 2003;18(6):485–6.

5. Ahmad FB, Jantan IB. The essential oils of *Boesenbergia stenophylla* R. M. Sm. as natural sources of methyl (E)-cinnamate. Flavour Fragr J. 2003 Nov;18(6):485–6.

6. Ahmad N, Alam MK, Shehbaz A, Khan A, Mannan A, Hakim SR, et al. Antimicrobial activity of clove oil and its potential in the treatment of vaginal candidiasis. Journal of drug targeting. 2005;13(10):555–61.

7. Ahuja A, Bakshi SK, Sharma SK, Thappa RK, Agarwal SG, Kichlu SK, et al. Production of volatile terpenes by proliferating shoots and micropropagated plants of *Santolina chamaecyparissus* L. (cotton lavender). Flavour and Fragrance Journal. 2005;20(4):403–6.

8. Ajaiyeoba EO, Ekundayo O. Essential oil constituents of *Aframomum melegueta* (Roscoe) K. Schum. seeds (alligator pepper) from Nigeria. Flavour and Fragrance Journal. 1999;14(2):109–11.

9. Ak G, Zengin G, Ceylan R, Fawzi Mahomoodally M, Jugreet S, Mollica A, et al. Chemical composition and biological activities of essential oils from *Calendula officinalis* L. flowers and leaves. Flavour and Fragrance Journal. 2021;36(5):554–63.

10. Alwakil NH, Mohamad Annuar MS, Jalil M. Synergistic Effects of Plant Growth Regulators and Elicitors on α-Humulene and Zerumbone Production in *Zingiber zerumbet* Smith Adventitious Root Cultures. Molecules. 2022 Jul 25;27(15):4744.

11. Andrade MS, Sampaio TS, Nogueira PCL, Ribeiro AS, Bittrich V, Amaral M. do CE. Volatile compounds of the leaves, flowers and fruits of *Kielmeyera rugosa* Choisy (Clusiaceae). Flavour and Fragrance Journal. 2007;22(1):49–52.

12. Andrianoelisoa HS, Menut C, de Chatelperron PC, Saracco J, Ramanoelina P, Danthu P. Intraspecific chemical variability and highlighting and chemotypes of leaf essential oils from *Ravensara aromatica* Sonnerat, a tree endemic to Madagascar. Flavour and Fragrance Journal. 2006;21(5):833–8.

Supplementary material

13. Apel MA, Sobral M, Menut C, Bassiere JM, Zuanazzi JA, Schapoval EES, et al. Volatile constituents of four Hexachlamys species growing in South Brazil. Flavour and Fragrance Journal. 2005;20(2):176–9.

14. Apel MA, Sobral M, Zuanazzi JA, Henriques AT. Essential oil composition of four Plinia species (Myrtaceae). Flavour and Fragrance Journal. 2006;21(3):565–7.

15. Apel MA, Sobral M, Zuanazzi JAS, Henriques AT. Essential oil composition of *Calycorectes australis* and *Calycorectes psidiiflorus* (Myrtaceae). Flavour and Fragrance Journal. 2006;21(4):656–8.

16. Ardekani NT, Khorram M, Zomorodian K, Yazdanpanah S, Veisi H. Evaluation of electrospun poly (vinyl alcohol)-based nanofiber mats incorporated with *Zataria multiflora* essential oil as potential wound dressing. International journal of biological macromolecules. 2019;125:743–50.

17. Jang HI, Rhee KJ, Eom YB. Antibacterial and antibiofilm effects of α-humulene against *Bacteroides fragilis*. Can J Microbiol. 2020 Jun;66(6):389–99.

18. AsadollahiBaboli M, Aghakhani A. Rapid analysis of *Origanum majorana* L. fragrance using a nanofiber sheet, gas chromatography with mass spectrometry, and chemometrics. Journal of Separation Science. 2014;37(8):990–6.

19. Avelar-Freitas BA, Almeida VG, Santos MG, Santos JAT, Barroso PR, Grael CFF, et al. Essential oil from *Ageratum fastigiatum* reduces expression of the pro-inflammatory cytokine tumor necrosis factor-alpha in peripheral blood leukocytes subjected to in vitro stimulation with phorbol myristate acetate. Revista Brasileira de Farmacognosia. 2015 Mar;25(2):129–33.

20. Awad NE, Kassem HA, Hamed MA, ElFeky AM, ElNaggar MAA. Hepatoprotective evaluation and isolation of the major secondary metabolites from the ethyl acetate extract of liquid culture filtrate of *Chaetomium globosum*. Biomedicine and Pharmacotherapy. 2018;97:174–80.

21. Ayoub N, AlAzizi M, Konig W, Kubeczka KH. Essential oils and a novel polyacetylene from *Eryngium yuccifolium* Michaux. (Apiaceae). Flavour and Fragrance Journal. 2006;21(6):864–8.

22. Ayuob NN, El Wahab MGA, Ali SS, AbdelTawab HS. *Ocimum basilicum* improve chronic stress-induced neurodegenerative changes in mice hippocampus. Metabolic brain disease. 2018;33(3):795–804.

23. Babu GDK, Shanmugam V, Ravindranath SD, Joshi VP. Comparison of chemical composition and antifungal activity of *Curcuma longa* L. leaf oils produced by different water distillation techniques. Flavour and Fragrance Journal. 2007;22(3):191–6.

24. Legault J, Dahl W, Debiton E, Pichette A, Madelmont JC. Antitumor activity of balsam fir oil: Production of reactive oxygen species induced by alpha-humulene as possible mechanism of action. Planta medica. 2003;69:402–7.

Supplementary material

25. Bader A, Caponi C, Cioni PL, Flamini G, Morelli I. Acorenone in the essential oil of flowering aerial parts of *Seseli tortuosum* L. Flavour and Fragrance Journal. 2003;18(1):57–8.

26. Bakr RO, El Bishbishy MH. Profile of bioactive compounds of *Capparis spinosa* var. Aegyptiaca growing in Egypt. Revista Brasileira de Farmacognosia. 2016;26(4):514–20.

27. Bakro F, Jedryczka M, Wielgusz K, Sgorbini B, Inchingolo R, Cardenia V. Simultaneous determination of terpenes and cannabidiol in hemp (*Cannabis sativa* L.) by fast gas chromatography with flame ionization detection. Journal of Separation Science. 2020;43(14):2817–26.

28. Balusamy SR, Perumalsamy H, Huq MA, Balasubramanian B. Anti-proliferative activity of *Origanum vulgare* inhibited lipogenesis and induced mitochondrial mediated apoptosis in human stomach cancer cell lines. Biomedicine and Pharmacotherapy. 2018;108:1835–44.

29. Baranauskiene R, Venskutonis PR, Demyttenaere JCR. Sensory and instrumental evaluation of sweet marjoram (*Origanum majorana* L.) aroma. Flavour and Fragrance Journal. 2005;20(5):492–500.

30. Baser KHC, Tabanca N, Ozek T, Demirci B, Duran A, Duman H. Composition of the essential oil of *Chaerophyllum aksekiense* A. Duran et Duman, a recently described endemic from Turkey. Flavour and Fragrance Journal. 2000;15(1):43–4.

31. Baser KHC, Ozek G, Ozek T, Duran A. Composition of the essential oil of *Centaurea huber-morathii* Wagenitz isolated from seeds by microdistillation. Flavour and Fragrance Journal. 2006;21(3):568–70.

32. Baser KHC, Ozek G, Ozek T, Duran A, Duman H. Composition of the essential oils of *Rhabdosciadium oligocarpum* (Post ex Boiss.) Hedge et Lamond and *Rhabdosciadium microcalycinum* Hand.-Mazz. Flavour and Fragrance Journal. 2006;21(4):650–5.

33. Basting RT, Spindola HM, Sousa IM de O, Queiroz N de CA, Trigo JR, de Carvalho JE, et al. *Pterodon pubescens* and *Cordia verbenacea* association promotes a synergistic response in antinociceptive model and improves the anti-inflammatory results in animal models. Biomedicine & Pharmacotherapy. 2019;112:108693–108693.

34. Belhadj S, Hentati O, Hammami M, Ben Hadj A, Boudawara T, Dammak M, et al. Metabolic impairments and tissue disorders in alloxan-induced diabetic rats are alleviated by *Salvia officinalis* L. essential oil. Biomedicine and Pharmacotherapy. 2018;108:985–95.

35. Ben Farhat M, Jordan MJ, Chaouech-Hamada R, Landoulsi A, Sotomayor JA. Variations in essential oil, phenolic compounds, and antioxidant activity of Tunisian cultivated *Salvia officinalis* L. Journal of Agricultural & Food Chemistry. 2009;57(21):10349–56.

Supplementary material

36. Bicchi C, Rubiolo P, Saranz Camargo EE, Vilegas W, de Souza Gracioso J, Monteiro Souza Brito AR. Components of *Turnera diffusa* Willd. var. afrodisiaca (Ward) Urb. essential oil. Flavour and Fragrance Journal. 2003;18(1):59–61.

37. Biondi DM, Sari M, Ghani ZA, Ruberto G. Essential oil of Algerian *Saccocalyx satureioides* Coss. et Durieu. Flavour and Fragrance Journal. 2006;21(3):546–8.

38. Blanc MC, Muselli A, Bradesi P, Casanova J. Chemical composition and variability of the essential oil of *Inula graveolens* from Corsica. Flavour and Fragrance Journal. 2004;19(4):314–314.

39. Blazquez MA, Perez I, Boira H. Essential oil analysis of *Teucrium libanitis* and *T. turredanum* by GC and GC-MS. Flavour and Fragrance Journal. 2003;18(6):497–501.

40. Block S, Flamini G, Brkic D, Morelli I, QuetinLeclercq J. Analysis of the essential oil from leaves of *Croton zambesicus* Muell. Arg. growing in Benin. Flavour and Fragrance Journal. 2006;21(2):222–4.

41. Borges R, Rojas LB, Cegarra JA, Usubillaga A. Study of the essential oils from the leaves and flowers of *Lepechinia conferta* (Benth) Epl. Flavour and Fragrance Journal. 2006;21(1):155–7.

42. Boszormenyi A, Hethelyi E, Farkas A, Horvath G, Papp N, Lemberkovics E, et al. Chemical and genetic relationships among sage (*Salvia officinalis* L.) cultivars and Judean sage (*Salvia judaica* Boiss.). Journal of Agricultural & Food Chemistry. 2009;57(11):4663–7.

43. Boti JB, Bighelli A, Cavaleiro C, Salgueiro L, Casanova J. Chemical variability of *Juniperus oxycedrus* ssp. oxycedrus berry and leaf oils from Corsica, analysed by combination of GC, GC-MS and 13C-NMR. Flavour and Fragrance Journal. 2006;21(2):268–73.

44. Boti JB, Koukoua G, N'Guessan TY, Casanova J. Chemical variability of *Conyza sumatrensis* and *Microglossa pyrifolia* from Cote d'Ivoire. Flavour and Fragrance Journal. 2007;22(1):27–31.

45. Boti JB, Yao PA, Koukoua G, N'Guessan TY, Casanova J. Components and chemical variability of *Isolona campanulata* Engler & Diels leaf oil. Flavour and Fragrance Journal. 2006;21(1):166–70.

46. Bouaziz M, Yangui T, Sayadi S, Dhouib A. Disinfectant properties of essential oils from *Salvia officinalis* L. cultivated in Tunisia. Food and Chemical Toxicology. 2009;47(11):2755–60.

47. Bougatsos C, Meyer JJM, Magiatis P, Vagias C, Chinou IB. Composition and antimicrobial activity of the essential oils of *Helichrysum kraussii* Sch. Bip. and *H. rugulosum* Less. from South Africa. Flavour and Fragrance Journal. 2003;18(1):48–51.

48. Boukhari F, TigrineKordjani N, Youcef Meklati B. Phytochemical investigation by microwave-assisted extraction of essential oil of the leaves of walnut cultivated in Algeria. *Helvetica chimica* acta. 2013;96(6):1168–75.

Supplementary material

49. Boutekedjiret C, Bentahar F, Belabbes R, Bessiere JM. Extraction of rosemary essential oil by steam distillation and hydrodistillation. Flavour and Fragrance Journal. 2003;18(6):481–4.

50. Brito MT, Ferreira RC, Beltrao DM, Moura APG, Xavier AL, Pita JCLR, et al. Antitumor activity and toxicity of volatile oil from the leaves of *Annona leptopetala*. Revista Brasileira de Farmacognosia. 2018;28(5):602–9.

51. Brophy JJ, Goldsack RJ, Bean AR, Forster PI, Lepsch BJ. Leaf essential oils of the genus Leptospermum (Myrtaceae) in eastern Australia. Part 6. *Leptospermum polygalifolium and* allies. Flavour and Fragrance Journal. 2000;15(4):271–7.

52. Brophy JJ, Goldsack RJ, Punruckvong A, Bean AR, Forster PI, Lepschi BJ, et al. Leaf essential oils of the genus Leptospermum (Myrtaceae) in eastern Australia. Part 7. *Leptospermum petersonii*, L. liversidgei and allies. Flavour and Fragrance Journal. 2000;15(5):342–51.

53. Brophy JJ, Goldsack RJ, Forster PI, Bean AR, Clarkson JR, Lepschi BJ. Leaf essential oils of the genus Leptospermum (Myrtaceae) in Eastern Australia. Part 1. *Leptospermum brachyandrum* and *Leptospermum pallidum* groups. Flavour and Fragrance Journal. 1998;13(1):19–25.

54. Caneschi CA, Martins FJ, Larrude DG, Romani EC, Brandao MAF, Raposo NRB. In vitro antifungal activity of *Baccharis trimera* less (DC) essential oil against dermatophytes. Tropical Journal of Pharmaceutical Research. 2015;14(11):2083–9.

55. Carrer RP, Vanderlinde R, Dutra S, Marcon A, Echeverrigaray S. Essential oil variation among Brazilian accessions of *Salvia guaranitica* L. Flavour and Fragrance Journal. 2007;22(5):430–4.

56. Carvalho HO, Santos IVFD, Rocha CFD, Barros ASA, Faria e Souza BS, Ferreira IM, et al. Effect of the treatment of *Copaifera duckei* Oleoresin (copaiba) in streptozotocin-induced diabetic rats. Revista Brasileira de Farmacognosia. 2018;28(6):724–31.

57. Cavalleri R, Becker JS, Pavan AM, Bianchetti P, Goettert MI, Ethur EM, et al. Essential oils rich in monoterpenes are unsuitable as additives to boar semen extender. Andrologia. 2018;50(8):no pagination-no pagination.

58. Cavalli JF, Tomi F, Bernardini AF, Casanova J. Chemical variability of the essential oil of *Helichrysum faradifani* Sc. Ell. from Madagascar. Flavour and Fragrance Journal. 2006;21(1):111–4.

59. Cavalli JF, Tomi F, Bernardini AF, Casanova J. Composition and chemical variability of the bark oil of *Cedrelopsis grevei* H. Baillon from Madagascar. Flavour and Fragrance Journal. 2003;18(6):532–8.

60. Cecchini C, Coman MM, Cresci A, Tirillini B, Cristalli G, Papa F, et al. Essential oil from fruits and roots of *Ferulago campestris* (Besser) Grecescu (Apiaceae): Composition and antioxidant and anti-Candida activity. Flavour and Fragrance Journal. 2010;25(6):493–502.

Supplementary material

61. Çelik G, Kılıç G, Kanbolat Ş, Özlem Şener S, Karaköse M, Yaylı N, et al. Biological activity, and volatile and phenolic compounds from five Lamiaceae species. Flavour and Fragrance Journal. 2021;36(2):223–32.

62. Chagonda LS, Chalchat JC. The essential oil of wild and cultivated *Hoslundia opposita* Vahl. from Zimbabwe. Flavour and Fragrance Journal. 2005;20(2):193–5.

63. Andrade-Ochoa S, Correa-Basurto J, Rodríguez-Valdez LM, Sánchez-Torres LE, Nogueda-Torres B, Nevárez-Moorillón GV. In vitro and in silico studies of terpenes, terpenoids and related compounds with larvicidal and pupaecidal activity against *Culex quinquefasciatus* Say (Diptera: Culicidae). Chemistry Central Journal. 2018 May 10;12(1):53.

64. Chagonda LS, Chalchat JC. The essential oil of the fruit of *Garcinia huillensis* Welw. ex. Oliv. from Zimbabwe. Flavour and Fragrance Journal. 2005;20(3):313–5.

65. Chaves AR, Silva SM, Queiroz RHC, Lanças FM, Queiroz MEC. Stir bar sorptive extraction and liquid chromatography with UV detection for determination of antidepressants in plasma samples. Journal of Chromatography B. 2007 May;850(1–2):295–302.

66. Checcucci A, Maida I, Bacci G, Ninno C, Bilia AR, Biffi S, et al. Is the plant-associated microbiota of Thymus spp. adapted to plant essential oil?. Research in microbiology. 2017;168(3):276–82.

67. Chen C, Chen H, Ni M, Yu F. Methyl jasmonate application and flowering stage affect scent emission of *Styrax japonicus*. Flavour and Fragrance Journal. 2021;36(4):497–504.

68. Chen X, Jin X, Li Y, Chen G, Chen K, Kan J. Preparation and characterization of molecularly-imprinted polymers for extraction of sanshool acid amide compounds followed by their separation from pepper oil resin derived from Chinese prickly ash (*Zanthoxylum bungeanum*). Journal of Separation Science. 2018;41(2):590–601.

69. Chen XB, Chen R, Luo ZR. Chemical composition and insecticidal properties of essential oil from aerial parts of *Mosla soochowensis* against two grain storage insects. Tropical Journal of Pharmaceutical Research. 2017;16(4):905–10.

70. Cheriti A, Saad A, Belboukhari N, Ghezali S. The essential oil composition of *Bubonium graveolens* (Forssk.) Maire from the Algerian Sahara. Flavour and Fragrance Journal. 2007;22(4):286–8.

71. Cho IH, Lee HJ, Kim YS. Differences in the volatile compositions of ginseng species (Panax sp.). Journal of Agricultural & Food Chemistry. 2012;60(31):7616–22.

72. Chu SS, Liu QZ, Du SS, Liu ZL. Chemical composition and insecticidal activity of the essential oil of the aerial parts of *Ostericum grosseserratum* (maxim) Kitag (Umbelliferae). Tropical Journal of Pharmaceutical Research. 2013;12(1):99–103.

73. Conti B, Benelli G, Flamini G, Cioni PL, Profeti R, Ceccarini L, et al. Larvicidal and repellent activity of *Hyptis suaveolens* (Lamiaceae) essential oil against the mosquito

Supplementary material

Aedes albopictus Skuse (Diptera: Culicidae). Parasitology research. 2012;110(5):2013–21.

74. Conti B, Flamini G, Cioni PL, Ceccarini L, Macchia M, Benelli G. Mosquitocidal essential oils: Are they safe against non-target aquatic organisms?. Parasitology research. 2014;113(1):251–9.

75. Couladis M, Tzakou O, Stojanovic D, MimicaDukic N, Jancic R. The essential oil composition of *Salvia argentea* L. Flavour and Fragrance Journal. 2001;16(3):227–9.

76. Couladis M, Tzakou O, MimicaDuki N, Jani R, Stojanovi D. Essential oil of Salvia officinalis L. from Serbia and Montenegro. Flavour and Fragrance Journal. 2002;17(2):119–26.

77. Cruz EMDO, CostaJunior LM, Pinto JAO, Santos DDA, Araujo SAD, ArrigoniBlank MDF, et al. Acaricidal activity of *Lippia gracilis* essential oil and its major constituents on the tick Rhipicephalus (Boophilus) microplus. Veterinary parasitology. 2013;195(1–2):198–202.

78. Cui B, Zheng T, Deng P, Zhang S, Zhao Z. Chemotaxonomic Variation in Volatile Component Contents in Ancient *Platycladus orientalis* Leaves with Different Tree Ages in Huangdi Mausoleum. Molecules. 2023 Feb 22;28(5):2043.

79. Cunha GH, Fechine FV, Frota Bezerra FA, Moraes MO, Silveira ER, Canuto KM, et al. Comparative study of the antihypertensive effects of hexane, chloroform and methanol fractions of essential oil of Alpinia zerumbet in rats Wistar. Revista Brasileira de Plantas Medicinais. 2016;18(1):113–24.

80. D'Auria FD, Tecca M, Strippoli V, Salvatore G, Battinelli L, Mazzanti G. Antifungal activity of *Lavandula angustifolia* essential oil against Candida albicans yeast and mycelial form. Medical Mycology. 2005;43(5):391–6.

81. Da Costa JS, Andrade WMS, De Figueiredo RO, Santos PVL, Freitas JJDS, Setzer WN, et al. Chemical Composition and Variability of the Volatile Components of Myrciaria Species Growing in the Amazon Region. Molecules. 2022 Mar 30;27(7):2234.

82. da Silva JD, Luz AIR, da Silva MHL, Andrade EHA, Zoghbi MGB, Maia JGS. Essential oils of the leaves and stems of four Psidium spp. Flavour and Fragrance Journal. 2003;18(3):240–3.

83. da Silva MHL, Andrade EHA, Maia JGS. The essential oil of *Pectis elongata* Kunth occurring in North Brazil. Flavour and Fragrance Journal. 2005;20(5):462–4.

84. Da Silva MHL, Andrade EHA, Zoghbi MDGB, Luz AIR, Da Silva JD, Maia JGS. The essential oils of *Lantana camara* L. occurring in North Brazil. Flavour and Fragrance Journal. 1999;14(4):208–10.

85. Dabiri M, Sefidkon F. Chemical composition of *Nepeta crassifolia* Boiss. & Buhse oil from Iran. Flavour and Fragrance Journal. 2003;18(3):225–7.

Supplementary material

86. Dabiri M, Sefidkon F. Analysis of the essential oil from aerial parts of *Perovskia atriplicifolia* Benth. at different stages of plant growth. Flavour and Fragrance Journal. 2001;16(6):435–8.

87. Das M, Ram G, Singh A, Mallavarapu GR, Ramesh S, Ram M, et al. Volatile constituents of different plant parts of *Chamomilla recutita* L. Rausch grown in the Indo-Gangetic plains. Flavour Fragr J. 2002 Jan;17(1):9–12.

88. Dawra M, El Rayess Y, El Beyrouthy M, Nehme N, El Hage R, Taillandier P, et al. Biological activities and chemical characterization of the Lebanese endemic plant *Origanum ehrenbergii* Boiss. Flavour and Fragrance Journal. 2021;36(3):339–51.

89. de Albuquerque RL, V SMG de, Machado MIL, A MFJ de, de Morais SM, Neto JS. Chemical composition and antioxidant activity of *Plectranthus grandis* and *P. ornatus* essential oils from north-eastern Brazil. Flavour and Fragrance Journal. 2007;22(1):24–6.

90. De Feo V, Soria EU, Soria RU, Senatore F. Chemical composition of essential oils of *Senecio nutans* Sch.-Bip. (Asteraceae). Flavour and Fragrance Journal. 2003;18(3):234–6.

91. De K. Martin MAC, Joseph H, Bercion S, Menut C. Chemical composition of essential oils from aerial parts of *Aframomum exscapum* (Sims) hepper collected in Guadeloupe, French West Indies. Flavour Fragr J. 2006 Nov;21(6):902–5.

92. de Vasconcelos Silva MG, de Abreu Matos FJ, Lacerda Machado MI, Aragao Craveiro A. Essential oils of *Ocimum basilicum* L., *O. basilicum*. var. minimum L. and *O. basilicum*. var. purpurascens Benth. grown in north-eastern Brazil. Flavour and Fragrance Journal. 2003;18(1):13–4.

93. Del C. Coronel A, Cerda-García-Rojas CM, Joseph-Nathan P, Catalán CAN. Chemical composition, seasonal variation and a new sesquiterpene alcohol from the essential oil of *Lippia integrifolia*. Flavour Fragr J. 2006 Sep;21(5):839–47.

94. Demirci B, Demirci F, Baser KHC. Headspace-SPME and hydrodistillation of two fragrant Artemisia sp. Flavour and Fragrance Journal. 2005;20(4):395–8.

95. Demirci B, Tsikolia M, Bernier UR, Agramonte NM, Alqasoumi SI, AlYahya MA, et al. *Phoenix dactylifera* L. spathe essential oil: Chemical composition and repellent activity against the yellow fever mosquito. Acta Tropica. 2013;128(3):557–60.

96. Demirpolat A, Akman F, Kazachenko AS. An Experimental and Theoretical Study on Essential Oil of *Aethionema sancakense*: Characterization, Molecular Properties and RDG Analysis. Molecules. 2022 Sep 19;27(18):6129.

97. Deterre S, Rega B, Delarue J, Decloux M, Lebrun M, Giampaoli P. Identification of key aroma compounds from bitter orange (*Citrus aurantium* L.) products: Essential oil and macerate-distillate extract. Flavour and Fragrance Journal. 2012;27(1):77–88.

98. Djarri L, Medjroubi K, Akkal S, Elomri A, Verite P. Composition of the essential oil of aerial parts of an endemic species of the Apiaceae of Algeria, *Daucus reboudii* Coss. Flavour and Fragrance Journal. 2006;21(4):647–9.

Supplementary material

99. Duarte PF, do Nascimento LH, Fischer B, Lohmann AM, Bandiera VJ, Fernandes IA, et al. Effect of Extraction Time on the Yield, Chemical Composition, and Antibacterial Activity of Hop Essential Oil Against Lactic Acid Bacteria (*Lactobacillus brevis* and *Lactobacillus casei*) Beer Spoilage. Current Microbiology. 2023;80(7):237–237.

100. Dudai N, Lewinsohn E, Larkov O, Katzir I, Ravid U, Chaimovitsh D, et al. Dynamics of yield components and essential oil production in a commercial hybrid sage (*Salvia officinalis* x *Salvia fruticosa* cv. Newe Ya'ar no. 4). Journal of Agricultural & Food Chemistry. 1999;47(10):4341–5.

101. Duschatzky CB, Almeida NV, Possetto M, Michis F, Scappini E, de Lampasona MP, et al. Essential oil composition of *Heterothalamus alienus* (Spreng.) Kuntze (Romerillo) from Argentina. Effect of harvesting period on the essential oil composition. Flavour and Fragrance Journal. 2007;22(1):39–41.

102. Dutta S, Mehrotra RC, Paul S, Tiwari RP, Bhattacharya S, Srivastava G, et al. Remarkable preservation of terpenoids and record of volatile signalling in plant-animal interactions from Miocene amber. Scientific Reports. 2017;7(1):10940–10940.

103. Dwivedi S, Khan M, Srivastava SK, Syamasunnder KV, Srivastava A. Essential oil composition of different accessions of Mentha x piperita L. grown on the northern plains of India. Flavour and Fragrance Journal. 2004;19(5):437–40.

104. Elmann A, Mordechay S, Rindner M, Larkov O, Elkabetz M, Ravid U. Protective Effects of the Essential Oil of *Salvia fruticosa* and Its Constituents on Astrocytic Susceptibility to Hydrogen Peroxide-Induced Cell Death. J Agric Food Chem. 2009 Aug 12;57(15):6636–41.

105. Erdem B, Bagci E, Dogan G, Aktoklu E, Dayangac A. Chemical composition and antimicrobial activities of essential oil and ethanol extract of *Cyperus fuscus* L burs from Turkey. Trop J Pharm Res. 2018 Oct 5;17(8):1637.

106. Evergetis E, Michaelakis A, Papachristos DP, Badieritakis E, Kapsaski-Kanelli VN, Haroutounian SA. Seasonal variation and bioactivity of the essential oils of two Juniperus species against Aedes (Stegomyia) albopictus (Skuse, 1894). Parasitol Res. 2016 Jun;115(6):2175–83.

107. Fanciullino AL, Tomi F, Luro F, Desjobert JM, Casanova J. Chemical variability of peel and leaf oils of mandarins. Flavour Fragr J. 2006 Mar;21(2):359–67.

108. Farah A, Afifi A, Fechtal M, Chhen A, Satrani B, Talbi M, et al. Fractional distillation effect on the chemical composition of Moroccan myrtle (Myrtus communis L.) essential oils. Flavour Fragr J. 2006 Mar;21(2):351–4.

109. Feijó EVRDS, De Oliveira RA, Costa LCDB. Light affects *Varronia curassavica* essential oil yield by increasing trichomes frequency. Revista Brasileira de Farmacognosia. 2014 Sep;24(5):516–23.

110. Feizbakhsh A, Pazoki H, Mohammadrezaei V, Ebrahimzadeh M. Effect of Phytohormones on the Composition of *Sambucus ebulus* Leaf Essential Oil. Trop J Pharm Res. 2014 May 28;13(4):573.

Supplementary material

111. Fekam Boyom F, Keumedjio F, Jazet Dongmo PM, Ngadjui BT, Amvam Zollo PH, Menut C, et al. Essential oils from *Croton zambesicus* Muell. Arg. growing in Cameroon. Flavour Fragr J. 2002 May;17(3):215–7.

112. Ferhat MA, Meklati BY, Chemat F. Comparison of different isolation methods of essential oil from Citrus fruits: cold pressing, hydrodistillation and microwave ‘dry’ distillation. Flavour Fragr J. 2007 Nov;22(6):494–504.

113. Fernandes MG, Gomes RA, Brito-Filho SG, Silva-Filho RN, Agra MF, Falcão-Silva VS, et al. Characterization and anti-staphylococcal activity of the essential oil from *Turnera subulata* Sm. Rev bras plantas med. 2014 Sep;16(3):534–8.

114. Fernandez X, Lizzani-Cuvelier L, Loiseau AM, Perichet C, Delbecque C, Arnaudo JF. Chemical composition of the essential oils from Turkish and Honduras Styrax. Flavour Fragr J. 2005 Jan;20(1):70–3.

115. Fernandez X, Pintaric C, Lizzani-Cuvelier L, Loiseau AM, Morello A, Pellerin P. Chemical composition of absolute and supercritical carbon dioxide extract of *Aframomum melegueta*. Flavour Fragr J. 2006 Jan;21(1):162–5.

116. Fernández-Ocaña AM, Gómez-Rodríguez MV, Velasco-Negueruela A, Camacho-Simarro AM, Fernández-López C, Altarejos J. In Vivo Antifungal Activity of the Essential Oil of *Bupleurum gibraltarium* against *Plasmopara halstedii* in Sunflower. J Agric Food Chem. 2004 Oct 1;52(21):6414–7.

117. Ferreira MJP, Costantin MB, Sartorelli P, Rodrigues GV, Limberger R, Henriques AT, et al. Computer-aided method for identification of components in essential oils by 13 C NMR spectroscopy. Analytica Chimica Acta. 2001 Nov;447(1–2):125–34.

118. Ferreira RO, Junior ARDC, Da Silva TMG, Castro RN, Da Silva TMS, De Carvalho MG. Distribution of metabolites in galled and non-galled leaves of *Clusia lanceolata* and its antioxidant activity. Revista Brasileira de Farmacognosia. 2014 Nov;24(6):617–25.

119. Fischer U, Lopez R, Pöll E, Vetter S, Novak J, Franz CM. Two chemotypes within *Lippia alba* populations in Guatemala. Flavour Fragr J. 2004 Jul;19(4):333–5.

120. Flamini G, Cioni PL, Morelli I. Essential oils of *Galeopsis pubescens* and *G. tetrahit* from Tuscany (Italy). Flavour Fragr J. 2004 Jul;19(4):327–9.

121. Fojtová J, Lojková L, Kubáň V. GC/MS of terpenes in walnut-tree leaves after accelerated solvent extraction. J Sep Sci. 2008 Jan;31(1):162–8.

122. Fokialakis N, Melliou E, Magiatis P, Harvala C, Mitaku S. Composition of the steam volatiles of six Euphorbia spp. from Greece. Flavour Fragr J. 2003 Jan;18(1):39–42.

123. Formisano C, Senatore F, Bruno M, Bellone G. Chemical composition and antimicrobial activity of the essential oil of *Phlomis ferruginea* Ten. (Lamiaceae) growing wild in Southern Italy. Flavour Fragr J. 2006 Sep;21(5):848–51.

124. Fournier G, Hadjiakhoondi A, Lebœuf M, Cavé A, Charles B. Essential Oils of Annonaceae. Part VII. Essential Oils of *Monanthotaxis diclina* (Sprague) Verdcourt and *Unonopsis guatterioides* R. E. Fries. Flavour Fragr J. 1997 Mar;12(2):95–8.

Supplementary material

125. Gabriele B, Fazio A, Dugo P, Costa R, Mondello L. Essential oil composition of *Citrus medica* L. Cv. Diamante (Diamante citron) determined after using different extraction methods. J Sep Sci. 2009 Jan;32(1):99–108.

126. Gagliano Candela R, Ilardi V, Badalamenti N, Bruno M, Rosselli S, Maggi F. Essential oil compositions of *Teucrium fruticans* , *T. scordium* subsp. *scordioides* and *T. siculum* growing in Sicily and Malta. Natural Product Research. 2021 Oct 18;35(20):3460–9.

127. Garneau FX, Collin G, Gagnon H, Jean FI, Strobl H, Pichette A. The essential oil composition of devil's club, *Oplopanax horridus* J. E. Smith Miq. Flavour Fragr J. 2006 Sep;21(5):792–4.

128. Giovannoni S, Lancioni C, Vaccarini C, Sedan D, Andrinolo D, Castells C. Determination of variability of terpenes and terpenoids in *Cannabis sativa* by gas chromatography-flame ionization detection and gas chromatography-mass spectrometry. Journal of Chromatography A. 2023 Jan;1687:463669.

129. Gohari AR, Hadjiakhoondi A, Sadat-Ebrahimi E, Saeidnia S, Shafiee A. Composition of the volatile oils of *Satureja spicigera* C. Koch Boiss. And *S. macrantha* C. A. Mey from Iran. Flavour Fragr J. 2006 Mar;21(2):348–50.

130. Gomes MVDS, Da Silva JD, Ribeiro AF, Cabral LM, De Sousa VP. Development and validation of a quantification method for α-humulene and trans-caryophyllene in *Cordia verbenacea* by high performance liquid chromatography. Revista Brasileira de Farmacognosia. 2019 Mar;29(2):182–90.

131. Gonçalves J, Figueira J, Rodrigues F, Câmara JS. Headspace solid-phase microextraction combined with mass spectrometry as a powerful analytical tool for profiling the terpenoid metabolomic pattern of hop-essential oil derived from Saaz variety: Other Techniques. J Sep Science. 2012 Sep;35(17):2282–96.

132. Gonçalves RDA, Pinheiro AB, Oliveira MAD, Nascimento RTD, Rosalem PF, Garcia VL, et al. Anatomical characters and chemical profile of leaves of three species in Lauraceae family. Revista Brasileira de Farmacognosia. 2018 Jan;28(1):1–8.

133. González S, Guerra PE, Bottaro H, Molares S, Demo MS, Oliva MM, et al. Aromatic plants from Patagonia. Part I. Antimicrobial activity and chemical composition of *Schinus polygamus* (Cav.) Cabrera essential oil. Flavour Fragr J. 2004 Jan;19(1):36–9.

134. Gooré SG, Ouattara ZA, Yapi AT, Békro YA, Bighelli A, Paoli M, et al. Chemical composition of the leaf oil of *Artabotrys jollyanus* from Côte d'Ivoire. Revista Brasileira de Farmacognosia. 2017 Jul;27(4):414–8.

135. Govindarajan M, Rajeswary M, Arivoli S, Tennyson S, Benelli G. Larvicidal and repellent potential of *Zingiber nimmonii* (J. Graham) Dalzell (Zingiberaceae) essential oil: an eco-friendly tool against malaria, dengue, and lymphatic filariasis mosquito vectors? Parasitol Res. 2016 May;115(5):1807–16.

136. Govindarajan M, Rajeswary M, Hoti SL, Bhattacharyya A, Benelli G. Eugenol, α-pinene and β-caryophyllene from *Plectranthus barbatus* essential oil as eco-friendly larvicides against malaria, dengue and Japanese encephalitis mosquito vectors. Parasitol Res. 2016 Feb;115(2):807–15.

Supplementary material

137. Havlik J, Kokoska L, Vasickova S, Valterova I. Chemical composition of essential oil from the seeds of *Nigella arvensis* L. and assessment of its actimicrobial activity. Flavour Fragr J. 2006 Jul;21(4):713–7.

138. Hoi TM, Satyal P, Huong LT, Hau DV, Binh TD, Duyen DTH, et al. Essential Oils from Vietnamese Asteraceae for Environmentally Friendly Control of Aedes Mosquitoes. Molecules. 2022 Nov 17;27(22):7961.

139. Houël E, Rodrigues AMS, Jahn-Oyac A, Bessière JM, Eparvier V, Deharo E, et al. *In vitro* antidermatophytic activity of *Otacanthus azureus* (Linden) Ronse essential oil alone and in combination with azoles. J Appl Microbiol. 2014 Feb;116(2):288–94.

140. Hymete A, Rohloff J, Iversen TH. Essential oil from seeds and husks of *Aframomum corrorima* from Ethiopia. Flavour Fragr J. 2006 Jul;21(4):642–4.

141. Jamoussi B, Romdhane M, Abderraba A, Hassine BB, Gadri AE. Effect of harvest time on the yield and composition of Tunisian myrtle oils. Flavour Fragr J. 2005 May;20(3):274–7.

142. Jantan I, Ahmad AS, Bakar SAA, Ahmad AR, Trockenbrodt M, Chak CV. Constituents of the essential oil of *Baeckea frutescens* L. from Malaysia. Flavour Fragr J. 1998 Jul;13(4):245–7.

143. Jantan IB, Ayop N, Mohd Ali NA, Ahmad AS, Yalvema MF, Muhammad K, et al. The essential oils of *Cinnamomum rhyncophyllum* Miq. as natural sources of benzyl benzoate, safrole and methyl(E)-cinnamate. Flavour Fragr J. 2004 May;19(3):260–2.

144. Jassbi AR, Ahmad VU, Tareen RB. Constituents of the essential oil of *Perovskia atriplicifolia* Benth. Flavour Fragr J. 1999 Jan;14(1):38–40.

145. Javidnia K, Miri R, Jafari A, Rezai H. Analysis of the volatile constituents of *Nepeta macrosiphon* Boiss. grown in Iran. Flavour Fragr J. 2004 Mar;19(2):156–8.

146. Jesus AS, Blank AF, Alves MF, Arrigoni-Blank MF, Lima RN, Alves PB. Influence of storage time and temperature on the chemical composition of the essential oil of *Hyptis pectinata* L. Poit. Rev bras plantas med. 2016;18(1 suppl 1):336–40.

147. Jiang C, Sun Y, Zhu X, Gao Y, Wang L, Wang J, et al. Solvent-free microwave extraction coupled with headspace single-drop microextraction of essential oils from flower of *Eugenia caryophyllata* Thunb. J Sep Science. 2010 Sep;33(17–18):2784–90.

148. Jiang J. Volatile composition of the laksa plant (*Polygonum hydropiper* L.), a potential source of green note aroma compounds. Flavour Fragr J. 2005 Sep;20(5):455–9.

149. Jinhua S, Yufei Z, Zhiyong Z, Xiaoming C, Fulin H. Chemical components of volatile oil from *Cinnamomum jensenianum* Hand Mazz leaf in Yongzhou, and its antibacterial and antioxidant properties. Trop J Pharm Res. 2018 Oct 3;17(9):1839.

150. Jirovetz L, Buchbauer G, Stoilova I, Stoyanova A, Krastanov A, Schmidt E. Chemical Composition and Antioxidant Properties of Clove Leaf Essential Oil. J Agric Food Chem. 2006 Aug 1;54(17):6303–7.

Supplementary material

151. Jovanovic T, Kitic D, Palic R, Stojanovic G, Ristic M. Chemical composition and antimicrobial activity of the essential oil of *Acinos arvensis* (Lam.) Dandy from Serbia. Flavour Fragr J. 2005 May;20(3):288–90.

152. Juliani HR, Zygadlo JA, Scrivanti R, De La Sota E, Simon JE. The essential oil of *Anemia tomentosa* (Savigny) Sw. var.anthriscifolia (Schrad.) Mickel. Flavour Fragr J. 2004 Nov;19(6):541–3.

153. Kambiré DA, Boti JB, Ouattara ZA, Yapi TA, Bighelli A, Tomi F, et al. Leaf essential oil from Ivorian *Isolona dewevrei* (Annonaceae): Chemical composition and structure elucidation of four new natural sesquiterpenes. Flavour Fragr J. 2021 Jan;36(1):22–33.

154. Kapoor R, Ali M, Mir SR, Rafiullah MRM. Essential oil constituents of aerial parts of *Artemisia scoparia* Waldst. & Kit. Flavour Fragr J. 2004 Mar;19(2):109–11.

155. Kasali AA, Ekundayo O, Winterhalter P, Koenig WA, Eshilokun AO. Chemical constituents of the essential oil of *Lippia adoensis* Hochst. ex Walp. Flavour Fragr J. 2004 May;19(3):210–2.

156. Keskin Ş. Orange peel volatile oil: A green solvent for propolis extraction, enhanced α-amylase inhibition activity. Flavour Fragr J. 2020 Jul;35(4):411–6.

157. Khan M, Srivastava SK, Jain N, Syamasundar KV, Yadav AK. Chemical composition of fruit and stem essential oils of *Lantana camara* from northern India. Flavour Fragr J. 2003 Sep;18(5):376–9.

158. Kim MR, Kim CW. Human blood plasma preparation for two-dimensional gel electrophoresis. Journal of Chromatography B. 2007 Apr;849(1–2):203–10.

159. Kim TH, Thuy NT, Shin JH, Baek HH, Lee HJ. Aroma-Active Compounds of Miniature Beefsteakplant (*Mosla dianthera* Maxim.). J Agric Food Chem. 2000 Jul 1;48(7):2877–81.

160. Kimbaris AC, Koliopoulos G, Michaelakis A, Konstantopoulou MA. Bioactivity of *Dianthus caryophyllus*, *Lepidium sativum*, *Pimpinella anisum*, and *Illicium verum* essential oils and their major components against the West Nile vector Culex pipiens. Parasitol Res. 2012 Dec;111(6):2403–10.

161. Kishimoto T, Wanikawa A, Kagami N, Kawatsura K. Analysis of Hop-Derived Terpenoids in Beer and Evaluation of Their Behavior Using the Stir Bar−Sorptive Extraction Method with GC-MS. J Agric Food Chem. 2005 Jun 1;53(12):4701–7.

162. Kitic D, Palic R, Ristic M, Sojanovic G, Jovanovic T. The volatile constituents of *Calamintha sylvatica* Bromf. subsp.sylvatica. Flavour Fragr J. 2001 Jul;16(4):257–8.

163. Kjeldsen F, Christensen LP, Edelenbos M. Quantitative Analysis of Aroma Compounds in Carrot (*Daucus carota* L.) Cultivars by Capillary Gas Chromatography Using Large-Volume Injection Technique. J Agric Food Chem. 2001 Sep 1;49(9):4342–8.

164. Kjeldsen F, Christensen LP, Edelenbos M. Changes in Volatile Compounds of Carrots (*Daucus carota* L.) During Refrigerated and Frozen Storage. J Agric Food Chem. 2003 Aug 1;51(18):5400–7.

Supplementary material

165. Koundal R, Kumar D, Walia M, Kumar A, Thakur S, Chand G, et al. Chemical and *in vitro* cytotoxicity evaluation of essential oil from *Eucalyptus citriodora* fruits growing in the Northwestern Himalaya, India: Chemical and *in vitro* cytotoxicity of the essential oil of *E . citriodora*. Flavour Fragr J. 2016 Mar;31(2):158–62.

166. Kowalski R, Wolski T. The chemical composition of essential oils of *Silphium perfoliatum* L. Flavour Fragr J. 2005 May;20(3):306–10.

167. Krishnamoorthy S, Chandrasekaran M, Raj GA, Jayaraman M, Venkatesalu V. Identification of chemical constituents and larvicidal activity of essential oil from *Murraya exotica* L. (Rutaceae) against *Aedes aegypti*, *Anopheles stephensi* and *Culex quinquefasciatus* (Diptera: Culicidae). Parasitol Res. 2015 May;114(5):1839–45.

168. Kukić J, Petrović S, Pavlović M, Couladis M, Tzakou O, Niketić M. Composition of essential oil of *Stachys alpina* L. ssp.dinarica Murb. Flavour Fragr J. 2006 May;21(3):539–42.

169. Kundakovic T, Fokialakis N, Kovacevic N, Chinou I. Essential oil composition of *Achillea lingulata* and *A. umbellata*. Flavour Fragr J. 2007 May;22(3):184–7.

170. Lago JHG, deÁvila P, De Aquino EM, Moreno PRH, Ohara MT, Limberger RP, et al. Volatile oils from leaves and stem barks of Cedrelafissilis (Meliaceae): chemical composition and antibacterial activities. Flavour Fragr J. 2004 Sep;19(5):448–51.

171. Langsdorf A, Drommershausen AL, Volkmar M, Ulber R, Holtmann D. Fermentative α-Humulene Production from Homogenized Grass Clippings as a Growth Medium. Molecules. 2022 Dec 8;27(24):8684.

172. Le NT, Donadu MG, Ho DV, Doan TQ, Le AT, Raal A, et al. Biological activities of essential oil extracted from leaves of *Atalantia sessiflora* Guillauminin Vietnam. J Infect Dev Ctries. 2020 Sep 30;14(09):1054–64.

173. Le TX, Ho ASH, Mah SH, Wong TW, Ong HC, Loh PHM, et al. Determination of borneol and other chemical compounds of essential oil of *Dryobalanops aromatica* exudate from Malaysia. Trop J Pharm Res. 2016 Jul 12;15(6):1293.

174. Le TX, Ho ASH, Mah SH, Wong TW, Ong HC, Loh PHM, et al. Determination of borneol and other chemical compounds of essential oil of *Dryobalanops aromatica* exudate from Malaysia. Trop J Pharm Res. 2016 Jul 12;15(6):1293.

175. Lemos M, Santin JR, Mizuno CS, Boeing T, De Sousa JPB, Nanayakkara D, et al. *Copaifera langsdorffii*: evaluation of potential gastroprotective of extract and isolated compounds obtained from leaves. Revista Brasileira de Farmacognosia. 2015 May;25(3):238–45.

176. Letchamo W, Ward W, Heard B, Heard D. Essential Oil of *Valeriana officinalis* L. Cultivars and Their Antimicrobial Activity As Influenced by Harvesting Time under Commercial Organic Cultivation. J Agric Food Chem. 2004 Jun 1;52(12):3915–9.

177. Lima MAS, Barros MCP, Pinheiro SM, Do Nascimento RF, De Abreu Matos FJ, Silveira ER. Volatile compositions of two Asteraceae from the north-east of Brazil:

Supplementary material

Ageratum conyzoides and *Acritopappus confertus* (Eupatorieae). Flavour Fragr J. 2005 Nov;20(6):559–61.

178. Lis A, Boczek E, Góra J. Chemical composition of the essential oils from fruits, leaves and flowers of the Amur cork tree (*Phellodendron amurense* Rupr.): ESSENTIAL OILS OF *PHELLODENDRON AMURENSE*. Flavour Fragr J. 2004 Nov;19(6):549–53.

179. Liu J, Nan P, Tsering Q, Tsering T, Bai Z, Wang L, et al. Volatile constituents of the leaves and flowers of *Salvia przewalskii* Maxim. from Tibet. Flavour Fragr J. 2006 May;21(3):435–8.

180. Liu XC, Liu QY, Zhou L, Liu QR, Liu ZL. Chemical Composition of *Zanthoxylum avicennae* Essential Oil and its Larvicidal Activity on Aedes albopictus Skuse. Trop J Pharm Res. 2016 Jun 23;13(3):399.

181. Lo Presti M, Ragusa S, Trozzi A, Dugo P, Visinoni F, Fazio A, et al. A comparison between different techniques for the isolation of rosemary essential oil. J Sep Science. 2005 Feb;28(3):273–80.

182. Lockwood GB, Asghari G, Hakimi B. Production of essential oil constituents by cultured cells of *Carum copticum* L. Flavour Fragr J. 2002 Nov;17(6):456–8.

183. López MA, Stashenko EE, Fuentes JL. Chemical composition and antigenotoxic properties of *Lippia alba* essential oils. Genet Mol Biol. 2011 Jul 29;34(3):479–88.

184. Lorenzo D, Loayza I, Dellacassa E. Composition of the essential oils from leaves of two Hedyosmum spp. from Bolivia. Flavour Fragr J. 2003 Jan;18(1):32–5.

185. Lorenzo D, Loayza I, Dellacassa E. Composition and chiral characterization of the essential oil of *Buddleja tucumanensis* from Bolivia. Flavour Fragr J. 2006 Jan;21(1):95–8.

186. Lorenzo D, Paz D, Davies P, Villamil J, Vila R, Cañigueral S, et al. Characterization and enantiomeric distribution of some terpenes in the essential oil of a Uruguayan biotype of *Salvia sclarea* L. Flavour Fragr J. 2004 Jul;19(4):303–7.

187. Machado JC, Lehnhardt F, Martins ZE, Kollmannsberger H, Gastl M, Becker T, et al. Prediction of Fruity-Citrus Intensity of Beers Dry Hopped with Mandarina Bavaria Based on the Content of Selected Volatile Compounds. J Agric Food Chem. 2020 Feb 19;68(7):2155–63.

188. Machado M, Dinis AM, Salgueiro L, Cavaleiro C, Custódio JBA, Sousa MDC. Anti-Giardia activity of phenolic-rich essential oils: effects of *Thymbra capitata*, *Origanum virens*, *Thymus zygis* subsp. sylvestris, and *Lippia graveolens* on trophozoites growth, viability, adherence, and ultrastructure. Parasitol Res. 2010 Apr;106(5):1205–15.

189. Magalhães LG, De Souza JM, Wakabayashi KAL, Da S. Laurentiz R, Vinhólis AHC, Rezende KCS, et al. In vitro efficacy of the essential oil of *Piper cubeba* L. (Piperaceae) against *Schistosoma mansoni*. Parasitol Res. 2012 May;110(5):1747–54.

Supplementary material

190. Maggi F, Tirillini B, Papa F, Sagratini G, Vittori S, Cresci A, et al. Chemical composition and antimicrobial activity of the essential oil of *Ferulago campestris* (Besser) Grecescu growing in central Italy. Flavour Fragr J. 2009 Nov;24(6):309–15.

191. Maia JGS, Andrade EHA, Carreira LMM, Oliveira J, Araújo JS. Essential oils of the Amazon Guatteria and Guatteriopsis species. Flavour Fragr J. 2005 Sep;20(5):478–80.

192. Maia JGS, Da Silva MHL, Andrade EHA, Rosa NA. Essential oil composition of *Scleria hirtella* Swartz (Cyperaceae). Flavour Fragr J. 2005 Sep;20(5):472–3.

193. Maietti S, Rossi D, Guerrini A, Useli C, Romagnoli C, Poli F, et al. A multivariate analysis approach to the study of chemical and functional properties of chemo-diverse plant derivatives: lavender essential oils: Chemodiversity and multivariate analysis: the lavender case. Flavour Fragr J. 2013 May;28(3):144–54.

194. Malenčić Dj, Couladis M, Mimica-Dukić N, Popović M, Boža P. Essential oils of three Salvia species from the Pannonian part of Serbia. Flavour Fragr J. 2004 May;19(3):225–8.

195. Marongiu B, Porcedda APS, Casu R, Pierucci P. Chemical composition of the oil and supercritical CO2 extract of *Schinus molle* L. Flavour Fragr J. 2004 Nov;19(6):554–8.

196. Marongiu B, Porcedda S, Piras A, Sanna G, Murreddu M, Loddo R. Extraction of *Juniperus communis* L. ssp.nana Willd. essential oil by supercritical carbon dioxide. Flavour Fragr J. 2006 Jan;21(1):148–54.

197. Marques FG, De Oliveira Neto JR, Da Cunha LC, De Paula JR, Bara MTF. Identification of terpenes and phytosterols in *Dipteryx alata* (baru) oil seeds obtained through pressing. Revista Brasileira de Farmacognosia. 2015 Sep;25(5):522–5.

198. Masola B, Oguntibeju OO, Oyenihi AB. *Centella asiatica* ameliorates diabetes-induced stress in rat tissues via influences on antioxidants and inflammatory cytokines. Biomedicine & Pharmacotherapy. 2018 May;101:447–57.

199. Masoudi S, Esmaeili A, Ali Khalilzadeh M, Rustaiyan A, Moazami N, Akhgar MR, et al. Volatile constituents of *Dorema aucheri* Boiss.,Seseli libanotis (L.) W. D. Koch var. armeniacum Bordz. and *Conium maculatum* L. three Umbelliferae herbs growing wild in Iran. Flavour Fragr J. 2006 Sep;21(5):801–4.

200. Meccia G, Rosquete C, Rojas LB, Feliciano AS. New labdane derivative from the essential oil of *Acalypha plicata* Müll.Arg. Flavour Fragr J. 2006 May;21(3):559–61.

201. Meekijjaroenroj A, Bessière JM, Anstett MC. Chemistry of floral scents in four Licuala species (Arecaceae). Flavour Fragr J. 2007 Jul;22(4):300–10.

202. Mendes GD, Hamamoto D, Ilha J, Pereira ADS, De Nucci G. Anastrozole quantification in human plasma by high-performance liquid chromatography coupled to photospray tandem mass spectrometry applied to pharmacokinetic studies. Journal of Chromatography B. 2007 May;850(1–2):553–9.

Supplementary material

203. Merle H, Verdeguer M, Blázquez MA, Boira H. Chemical composition of the essential oils from *Eriocephalus africanus* L. var.africanus populations growing in Spain. Flavour Fragr J. 2007 Nov;22(6):461–4.

204. Mevy JP, Bessiere JM, Rabier J, Dherbomez M, Ruzzier M, Millogo J, et al. Composition and antimicrobial activities of the essential oil of *Triumfetta rhomboidea* Jacq. Flavour Fragr J. 2006 Jan;21(1):80–3.

205. Mezzoug N, Elhadri A, Dallouh A, Amkiss S, Skali NS, Abrini J, et al. Investigation of the mutagenic and antimutagenic effects of *Origanum compactum* essential oil and some of its constituents. Mutation Research/Genetic Toxicology and Environmental Mutagenesis. 2007 May;629(2):100–10.

206. Michielin EMZ, Salvador AA, Riehl CAS, Smânia A, Smânia EFA, Ferreira SRS. Chemical composition and antibacterial activity of *Cordia verbenacea* extracts obtained by different methods. Bioresource Technology. 2009 Dec;100(24):6615–23.

207. Mir SR, Ali M, Kapoor R. Chemical composition of essential oil of *Cinnamomum tamala* Nees et Eberm. leaves. Flavour Fragr J. 2004 Mar;19(2):112–4.

208. Mirjalili MH, Salehi P, Badi HN, Sonboli A. Volatile constituents of the flowerheads of three Echinacea species cultivated in Iran. Flavour Fragr J. 2006 Mar;21(2):355–8.

209. Mirza M, Ahmadi L, Tayebi M. Volatile constituents of *Hymenocrater incanus* Bunge, an Iranian endemic species. Flavour Fragr J. 2001 Jul;16(4):239–40.

210. Mirza M, Nik ZB. Volatile constituents of *Phlomis olivieri* Benth. from Iran. Flavour Fragr J. 2003 Mar;18(2):131–2.

211. Miyazawa M, Yamafuji C, Kurose K, Ishikawa Y. Volatile components of the rhizomes of *Cirsium japonicum* DC. Flavour Fragr J. 2003 Jan;18(1):15–7.

212. Moemenbellah-Fard MD, Abdollahi A, Ghanbariasad A, Osanloo M. Antibacterial and leishmanicidal activities of *Syzygium aromaticum* essential oil versus its major ingredient, eugenol. Flavour Fragr J. 2020 Sep;35(5):534–40.

213. Mohammadi Pour P, Bidad S, Bahrami G, Hosseinzadeh L, Mojarrab M, Farzaei MH. Evaluation of the Cytotoxicity of Aqueous Extract and Oleo-Essential Oil of *Dorema ammoniacum* Plant Oleo-Gum Resin in Some Human Cancer Cell Lines. Amantini C, editor. Analytical Cellular Pathology. 2022 Aug 9;2022:1–9.

214. Mondêgo-Oliveira R, De Sá Sousa JC, Moragas-Tellis CJ, De Souza PVR, Dos Santos Chagas MDS, Behrens MD, et al. *Vernonia brasiliana* (L.) Druce induces ultrastructural changes and apoptosis-like death of *Leishmania infantum* promastigotes. Biomedicine & Pharmacotherapy. 2021 Jan;133:111025.

215. Mukherjee M, Blair RH, Wang ZQ. Machine-learning guided elucidation of contribution of individual steps in the mevalonate pathway and construction of a yeast platform strain for terpenoid production. Metabolic Engineering. 2022 Nov;74:139–49.

Supplementary material

216. Musenga A, Ferranti A, Saracino MA, Fanali S, Raggi MA. Simultaneous determination of aromatic and terpenic constituents of cloves by means of HPLC with diode array detection. J Sep Sci. 2006 Jun;29(9):1251–8.

217. Musenga A, Mandrioli R, Ferranti A, D'Orazio G, Fanali S, Raggi MA. Analysis of aromatic and terpenic constituents of pepper extracts by capillary electrochromatography. J Sep Sci. 2007 Mar;30(4):612–9.

218. Najar B, Pistelli L, Mancini S, Fratini F. Chemical composition and in vitro antibacterial activity of essential oils from different species of *Juniperus* (section *Juniperus*). Flavour Fragr J. 2020 Nov;35(6):623–38.

219. Ng F, Basri N, Wu W, Thong A, Thong G, Chew W, et al. Characterization of volatile compounds in Ylang-Ylang essential oils from Comoros and Madagascar by gas chromatography and principal component analysis. Flavour Fragr J. 2021 Jan;36(1):159–66.

220. Ngassapa OD, Runyoro DKB, Vagionas K, Graikou K, Chinou IB. Chemical Composition and Antimicrobial Activity of *Geniosporum rotundifolium* Briq and *Haumaniastrum villosum* (Bene) AJ Paton (Lamiaceae) Essential Oils from Tanzania. Trop J Pharm Res. 2016 May 11;15(1):107.

221. Niu JF, Wang GC, Lin X zhi, Zhou BC. Large-scale recovery of C-phycocyanin from *Spirulina platensis* using expanded bed adsorption chromatography. Journal of Chromatography B. 2007 May;850(1–2):267–76.

222. Norouzi-Arasi H, Yavari I, Chalabian F, Kiarostami V, Ghaffarzadeh F, Nasirian A. Chemical constituents and antimicrobial activities of the essential oil of *Acroptilon repens* (L.) DC. Flavour Fragr J. 2006 Mar;21(2):247–9.

223. Novak J, Langbehn J, Pank F, Franz CM. Essential oil compounds in a historical sample of marjoram (*Origanum majorana* L., Lamiaceae). Flavour Fragr J. 2002 May;17(3):175–80.

224. Novak J, Zitterl-Eglseer K, Deans SG, Franz CM. Essential oils of different cultivars of *Cannabis sativa* L. and their antimicrobial activity. Flavour Fragr J. 2001 Jul;16(4):259–62.

225. Ogunwande IA, Olawore NO, Kasali AA, König WA. Chemical composition of the leaf volatile oils of *Callitris intratropica* R. T. Baker & H. G. Smith from Nigeria: LEAF VOLATILE OIL OF *CALLITRIS INTRATROPICA*. Flavour Fragr J. 2003 Sep;18(5):387–9.

226. Olawore NO, Ogunwande IA, Ekundayo O, Adeleke KA. Chemical composition of the leaf and fruit essential oils of *Murraya paniculata* (L.) Jack. (Syn.Murraya exotica Linn.). Flavour Fragr J. 2005 Jan;20(1):54–6.

227. Oliveira GL, Moreira DDL, Mendes ADR, Guimarães EF, Figueiredo LS, Kaplan MAC, et al. Growth study and essential oil analysis of *Piper aduncum* from two sites of Cerrado biome of Minas Gerais State, Brazil. Revista Brasileira de Farmacognosia. 2013 Sep;23(5):743–53.

Supplementary material

228. Ottavioli J, Bighelli A, Casanova J. Diterpene-rich needle oil of *Pinus pinaster* Ait. from Corsica. Flavour Fragr J. 2008 Mar;23(2):121–5.

229. Ouamba JM, Ouabonzi A, Ekouya A, Bessière JM, Menut C, Abena AA, et al. Volatile constituents of the essential oil leaf of *Lantana salvifolia* Jacq. (Verbenaceae). Flavour Fragr J. 2006 Jan;21(1):158–61.

230. Oyedeji OA, Ekundayo O, König WA. Volatile leaf oil constituents of *Lantana camara* L from Nigeria: VOLATILE LEAF OIL OF *LANTANA CAMARA*. Flavour Fragr J. 2003 Sep;18(5):384–6.

231. Palmeira SF, Moura FDS, Alves VDL, Oliveira FMD, Bento ES, Conserva LM, et al. Neutral components from hexane extracts of *Croton sellowii*. Flavour Fragr J. 2004 Jan;19(1):69–71.

232. Paolini J, Muselli A, Bernardini AF, Bighelli A, Casanova J, Costa J. Thymol derivatives from essential oil of *Doronicum corsicum* L. Flavour Fragr J. 2007 Nov;22(6):479–87.

233. Parrot S, Lambás-Señas L, Sentenac S, Denoroy L, Renaud B. Highly sensitive assay for the measurement of serotonin in microdialysates using capillary high-performance liquid chromatography with electrochemical detection. Journal of Chromatography B. 2007 May;850(1–2):303–9.

234. Pavlović M, Tzakou O, Petrakis PV, Couladis M. The essential oil of *Hypericum perforatum* L., *Hypericum tetrapterum* Fries and *Hypericum olympicum* L. growing in Greece. Flavour Fragr J. 2006 Jan;21(1):84–7.

235. Pavlović M, Kovačević N, Tzakou O, Couladis M. Essential oil composition of *Anthemis triumfetti* (L.) DC. Flavour Fragr J. 2006 Mar;21(2):297–9.

236. Péres VF, Moura DJ, Sperotto ARM, Damasceno FC, Caramão EB, Zini CA, et al. Chemical composition and cytotoxic, mutagenic and genotoxic activities of the essential oil from *Piper gaudichaudianum* Kunth leaves. Food and Chemical Toxicology. 2009 Sep;47(9):2389–95.

237. Petrakis PV, Tsitsimpikou C, Tzakou O, Couladis M, Vagias C, Roussis V. Needle volatiles from five Pinus species growing in Greece. Flavour Fragr J. 2001 Jul;16(4):249–52.

238. Pitarokili D, Couladis M, Petsikos-Panayotarou N, Tzakou O. Composition and Antifungal Activity on Soil-Borne Pathogens of the Essential Oil of *Salvia sclarea* from Greece. J Agric Food Chem. 2002 Nov 1;50(23):6688–91.

239. Podduturi R, Petersen MA, Mahmud S, Rahman MdM, Jørgensen NOG. Potential Contribution of Fish Feed and Phytoplankton to the Content of Volatile Terpenes in Cultured Pangasius (*Pangasianodon hypophthalmus*) and Tilapia (*Oreochromis niloticus*). J Agric Food Chem. 2017 May 10;65(18):3730–6.

240. Pourmortazavi SM, Sefidkon F, Hosseini SG. Supercritical Carbon Dioxide Extraction of Essential Oils from *Perovskia atriplicifolia* Benth. J Agric Food Chem. 2003 Aug 1;51(18):5414–9.

Supplementary material

241. Pripdeevech P, Chukeatirote E. Chemical compositions, antifungal and antioxidant activities of essential oil and various extracts of *Melodorum fruticosum* L. flowers. Food and Chemical Toxicology. 2010 Oct;48(10):2754–8.

242. Qnais E, Bseiso Y, Wedyan M, Al-Omari M, Alkhateeb H. Chemical composition and antinociceptive effects of essential oil from aerial parts of *Gundelia tournefortii* L Asteraceae (Compositae) in rats. Trop J Pharm Res. 2016 Nov 15;15(10):2183.

243. Quijano CE, Salamanca G, Pino JA. Aroma volatile constituents of Colombian varieties of mango (*Mangifera indica* L.). Flavour Fragr J. 2007 Sep;22(5):401–6.

244. Rajeswara Rao BR, Sastry KP, Saleem SM, Prakasa Rao EVS, Syamasundar KV, Ramesh S. Volatile flower oils of three genotypes of rose-scented geranium (Pelargonium sp.). Flavour Fragr J. 2000 Mar;15(2):105–7.

245. Rana VS, Juyal JP, Blazquez MA, Bodakhe SH. Essential oil composition of *Artemisia parviflora* aerial parts. Flavour Fragr J. 2003 Jul;18(4):342–4.

246. Ravi Kiran S, Sita Devi P. Evaluation of mosquitocidal activity of essential oil and sesquiterpenes from leaves of *Chloroxylon swietenia* DC. Parasitol Res. 2007 Jul;101(2):413–8.

247. Rezende WP, Borges LL, Alves NM, Ferri PH, Paula JR. Chemical variability in the essential oils from leaves of *Syzygium jambos*. Revista Brasileira de Farmacognosia. 2013 May;23(3):433–40.

248. Rodrigues FSLM, Antunes LCS, Figueiredo AC, Costa MM, Pereira JDS, Colaço RDR, et al. Composition of the leaf, flower and fruit volatile oils of *Pittosporum tobira* (Thunb.) W. T. Aiton grown in three locations in Portugal. Flavour Fragr J. 2007 Jul;22(4):311–6.

249. Rout PK, Rao YR, Sree A, Naik SN. Composition of essential oil, concrete, absolute, wax and headspace volatiles of *Murrarya paniculata* (Linn.) Jack flowers. Flavour Fragr J. 2007 Sep;22(5):352–7.

250. Sá SD, Fiuza TS, Borges LL, Ferreira HD, Tresvenzol LMF, Ferri PH, et al. Chemical composition and seasonal variability of the essential oils of leaves and morphological analysis of *Hyptis carpinifolia*. Revista Brasileira de Farmacognosia. 2016 Nov;26(6):688–93.

251. Saei-Dehkordi SS, Tajik H, Moradi M, Khalighi-Sigaroodi F. Chemical composition of essential oils in *Zataria multiflora* Boiss. from different parts of Iran and their radical scavenging and antimicrobial activity. Food and Chemical Toxicology. 2010 Jun;48(6):1562–7.

252. Sajjadi SE, Ghassemi N. Volatile constituents of *Nepeta glomerulosa* Boiss. subsp.carmanica. Flavour Fragr J. 1999 Sep;14(5):265–7.

253. Sajjadi SE, Mehregan I, Khatamsaz M, Asgari Gh. Chemical composition of the essential oil of *Perovskia abrotanoides* Karel. growing wild in Iran. Flavour Fragr J. 2005 Jul;20(4):445–6.

Supplementary material

254. Sakurai K, Tomiyama K, Yaguchi Y, Asakawa Y. The characteristic smell emitted from two scale insects, *Ceroplastes japonicus* and *Ceroplastes rubens*. Bioscience, Biotechnology, and Biochemistry. 2020 Aug 2;84(8):1541–5.

255. Santos AP, Lopes MC, Limberger RP, Apel MA, Henriques AT, Moreno PRH. Analysis of the volatile oil from *Pilocarpus pennatifolius* Lemmaire (Rutaceae) leaves by GC–MS. Flavour Fragr J. 2004 Jul;19(4):325–6.

256. Santos DL, Ferreira HD, Borges LL, Paula JR, Tresvenzol LMF, Santos PA, et al. Chemical composition of essential oils of leaves, flowers and fruits of *Hortia oreadica*. Revista Brasileira de Farmacognosia. 2016 Jan;26(1):23–8.

257. Santos-Gomes PC, Fernandes-Ferreira M. Organ- and Season-Dependent Variation in the Essential Oil Composition of *Salvia officinalis* L. Cultivated at Two Different Sites. J Agric Food Chem. 2001 Jun 1;49(6):2908–16.

258. Santos-Gomes PC, Fernandes-Ferreira M. Essential Oils Produced by in Vitro Shoots of Sage (*Salvia officinalis* L.). J Agric Food Chem. 2003 Apr 1;51(8):2260–6.

259. Sari M, Biondi DM, Kaâbeche M, Mandalari G, D'Arrigo M, Bisignano G, et al. Chemical composition, antimicrobial and antioxidant activities of the essential oil of several populations of Algerian *Origanum glandulosum* Desf. Flavour Fragr J. 2006 Nov;21(6):890–8.

260. Sarkhail P, Amin G, Sha?ee A. Composition of the essential oil of *Phlomis persica* Boiss and *Phlomis chorassanica* Bunge from Iran. Flavour Fragr J. 2004 Nov;19(6):538–40.

261. Saroglou V, Arfan M, Shabir A, Hadjipavlou-Litina D, Skaltsa H. Composition and antioxidant activity of the essential oil of *Teucrium royleanum* Wall. ex Benth growing in Pakistan. Flavour Fragr J. 2007 Mar;22(2):154–7.

262. Satou T, Kasuya H, Takahashi M, Murakami S, Hayashi S, Sadamoto K, et al. Relationship between duration of exposure and anxiolytic-like effects of essential oil from *Alpinia zerumbet*: Effects of essential oil from *Alpinia zerumbet*. Flavour Fragr J. 2011 May;26(3):180–5.

263. Sefidkon F. Essential oil of *Lantana camara* L. occurring in Iran. Flavour Fragr J. 2002 Jan;17(1):78–80.

264. Sefidkon F, Khajavi MS. Chemical composition of the essential oils of two Salvia species from Iran: *Salvia verticillata* L. and *Salvia santolinifolia* Boiss. Flavour Fragr J. 1999 Mar;14(2):77–8.

265. Senatore F, Landolfi S, Celik S, Bruno M. Volatile components of *Centaurea calcitrapa* L. and *Centaurea sphaerocephala* L. ssp.sphaerocephala, two Asteraceae growing wild in Sicily. Flavour Fragr J. 2006 Mar;21(2):282–5.

266. Senthilkumar A, Venkatesalu V. Chemical composition and larvicidal activity of the essential oil of *Plectranthus amboinicus* (Lour.) Spreng against *Anopheles stephensi*: a malarial vector mosquito. Parasitol Res. 2010 Oct;107(5):1275–8.

Supplementary material

267. Shafi PM, Jose B, Radhamani KT, Clery RA. Influence of pH on essential oil composition of *Zanthoxylum rhetsa* seeds obtained by steam distillation. Flavour Fragr J. 2006 Mar;21(2):317–8.

268. Sharma A, Rajendran S, Srivastava A, Sharma S, Kundu B. Antifungal activities of selected essential oils against *Fusarium oxysporum* f. sp. lycopersici 1322, with emphasis on *Syzygium aromaticum* essential oil. Journal of Bioscience and Bioengineering. 2017 Mar;123(3):308–13.

269. Sharmeen Jugreet B, Kouadio Ibrahime S, Zengin G, Abdallah HH, Fawzi Mahomoodally M. GC/MS Profiling, *In Vitro* and *In Silico* Pharmacological Screening and Principal Component Analysis of Essential Oils from Three Exotic and Two Endemic Plants from Mauritius. Chem Biodiversity [Internet]. 2021 Mar [cited 2023 Aug 16];18(3). Available from: https://onlinelibrary.wiley.com/doi/10.1002/cbdv.202000921

270. Shimizu Y, Imayoshi Y, Kato M, Maeda K, Iwabuchi H, Shimomura K. Volatiles from leaves of field-grown plants and shoot cultures of *Gynura bicolor* DC. Flavour Fragr J. 2009 Sep;24(5):251–8.

271. Shimizu Y, Imayoshi Y, Kato M, Maeda K, Iwabuchi H, Shimomura K. New eudesmane-type sesquiterpenoids and other volatile constituents from the roots of *Gynura bicolor* DC. Flavour Fragr J. 2011 Jan;26(1):55–64.

272. Silva GNS, Spader TB, Alves SH, Mallmann CA, Heinzmann BM. Composition and evaluation of the antimicrobial activity of the essential oil of *Senecio selloi* Spreng DC. Rev bras plantas med. 2013;15(4):503–7.

273. Silva Lima A, Milhomem MN, Santos Monteiro O, Arruda ACP, De Castro JAM, Fernandes YML, et al. Seasonal analysis and acaricidal activity of the thymol-type essential oil of *Ocimum gratissimum* and its major constituents against *Rhipicephalus microplus* (Acari: Ixodidae). Parasitol Res. 2018 Jan;117(1):59–65.

274. Sinan KI, Etienne OK, Stefanucci A, Mollica A, Mahomoodally MF, Jugreet S, et al. Chemodiversity and biological activity of essential oils from three species from the *Euphorbia* genus. Flavour Fragr J. 2021 Jan;36(1):148–58.

275. Singh AK, Raina VK, Naqvi AA, Patra NK, Kumar B, Ram P, et al. Essential oil composition and chemoarrays of menthol mint (*Mentha arvensis* L. f.piperascens Malinvaud ex. Holmes) cultivars. Flavour Fragr J. 2005 May;20(3):302–5.

276. Skalicka-Woźniak K, Walasek M, Ludwiczuk A, Głowniak K. Isolation of terpenoids from *Pimpinella anisum* essential oil by high-performance counter-current chromatography: Liquid Chromatography. J Sep Science. 2013 Aug;36(16):2611–4.

277. Sonboli A, Azizian D, Yousefzadi M, Kanani MR, Mehrabian AR. Volatile constituents and antimicrobial activity of the essential oil of *Tetrataenium lasiopetalum* (Apiaceae) from Iran. Flavour Fragr J. 2007 Mar;22(2):119–22.

278. Sperotto ARM, Moura DJ, Péres VF, Damasceno FC, Caramão EB, Henriques JAP, et al. Cytotoxic mechanism of *Piper gaudichaudianum* Kunth essential oil and its major compound nerolidol. Food and Chemical Toxicology. 2013 Jul;57:57–68.

Supplementary material

279. Srivastava AK, Srivastava SK, Syamsundar KV. Volatile composition of *Curcuma angustifolia* Roxb. rhizome from central and southern India. Flavour Fragr J. 2006 May;21(3):423–6.

280. Stevanovic T, Garneau FX, Jean FI, Gagnon H, Vilotic D, Petrovic S, et al. The essential oil composition of *Pinus mugo* Turra from Serbia. Flavour Fragr J. 2005 Jan;20(1):96–7.

281. Su YC, Ho CL, Wang EIC. Analysis of leaf essential oils from the indigenous ve conifers of Taiwan. Flavour Fragr J. 2006 May;21(3):447–52.

282. Sulborska-Różycka A, Weryszko-Chmielewska E, Polak B, Stefańczyk B, Matysik-Woźniak A, Rejdak R. Secretory Products in Petals of *Centaurea cyanus* L. Flowers: A Histochemistry, Ultrastructure, and Phytochemical Study of Volatile Compounds. Molecules. 2022 Feb 17;27(4):1371.

283. Šulniūtė V, Baranauskienė R, Ragažinskienė O, Venskutonis PR. Comparison of composition of volatile compounds in ten *Salvia* species isolated by different methods. Flavour Fragr J. 2017 Jul;32(4):254–64.

284. Sun D, Petracek PD. Grapefruit Gland Oil Composition Is Affected by Wax Application, Storage Temperature, and Storage Time. J Agric Food Chem. 1999 May 1;47(5):2067–9.

285. Sundufu AJ, Shoushan H. Chemical composition of the essential oils of *Lantana camara* L. occurring in south China. Flavour Fragr J. 2004 May;19(3):229–32.

286. Sutour S, Bradesi P, De Rocca-Serra D, Casanova J, Tomi F. Chemical composition and antibacterial activity of the essential oil from *Mentha suaveolens* ssp.insularis (Req.) Greuter. Flavour Fragr J. 2008 Mar;23(2):107–14.

287. Szafranek B, Chrapkowska K, Pawińska M, Szafranek J. Analysis of Leaf Surface Sesquiterpenes in Potato Varieties. J Agric Food Chem. 2005 Apr 1;53(8):2817–22.

288. Tabanca N, Demirci B, Baser KHC, Mincsovics E, Khan SI, Jacob MR, et al. Characterization of volatile constituents of *Scaligeria tripartita* and studies on the antifungal activity against phytopathogenic fungi. Journal of Chromatography B. 2007 May;850(1–2):221–9.

289. Tavares Trindade FT, Stabeli RG, Pereira AA, Facundo VA, Almeida E Silva AD. *Copaifera multijuga* ethanolic extracts, oilresin, and its derivatives display larvicidal activity against *Anopheles darlingi* and *Aedes aegypti* (Diptera: Culicidae). Revista Brasileira de Farmacognosia. 2013 May;23(3):464–70.

290. Tirillini B, Pellegrino R, Bini LM. Essential oil composition of *Stachys sylvatica* L. from Italy. Flavour Fragr J. 2004 Jul;19(4):330–2.

291. Tonzibo ZF, Coffy AA, Chalachat JC, N'guessan YT. Chemical composition of essential oils of *Hoslundia opposita* Vahl. from Ivory Coast. Flavour Fragr J. 2006 Sep;21(5):789–91.

Supplementary material

292. Tosun A, Kürkçüoglu M, Dogan E, Duman H, Başer KHC. Essential oil composition of *Seseli petraeum* M. Bieb. and *Seseli andronakii* Woron. growing in Turkey. Flavour Fragr J. 2006 Mar;21(2):257–9.

293. Trilles BL, Bombarda I, Bouraïma-Madjebi S, Raharivelomanana P, Bianchini JP, Gaydou EM. Occurrence of various chemotypes in niaouli [*Melaleuca quinquenervia* (Cav.) S. T. Blake] essential oil from New Caledonia. Flavour Fragr J. 2006 Jul;21(4):677–82.

294. Tzakou O, Couladis M, Slavkovska V, Mimica-Dukic N, Jancic R. The essential oil composition of *Salvia brachyodon* Vandas. Flavour Fragr J. 2003 Jan;18(1):2–4.

295. Uçar G, Balaban M, Usta M. Volatile needle and wood extracts of oriental spruce *Picea orientalis* (L.) Link: VOLATILE OILS OF *PICEA ORIENTALIS*. Flavour Fragr J. 2003 Sep;18(5):368–75.

296. Vallejo MCG, Moujir L, Burillo J, Guerra LL, González M, Peñate RD, et al. Chemical composition and biological activities of the essential oils of *Salvia canariensis*. Flavour Fragr J. 2006 Jan;21(1):72–6.

297. Van Opstaele F, Praet T, Aerts G, De Cooman L. Characterization of Novel Single-Variety Oxygenated Sesquiterpenoid Hop Oil Fractions via Headspace Solid-Phase Microextraction and Gas Chromatography–Mass Spectrometry/Olfactometry. J Agric Food Chem. 2013 Nov 6;61(44):10555–64.

298. Vasilijević B, Knežević-Vukčević J, Mitić-Ćulafić D, Orčić D, Francišković M, Srdic-Rajic T, et al. Chemical characterization, antioxidant, genotoxic and in vitro cytotoxic activity assessment of *Juniperus communis* var. saxatilis. Food and Chemical Toxicology. 2018 Feb;112:118–25.

299. Vieira RF, Simon JE. Chemical characterization of basil (Ocimum spp.) based on volatile oils. Flavour Fragr J. 2006 Mar;21(2):214–21.

300. Vila R, Mundina M, Tomi F, Cicció JF, Gupta MP, Iglesias J, et al. Constituents of the essential oils from *Piper friedrichsthalii* C.DC. and *P. pseudolindenii* C.DC. from Central America: ESSENTIAL OILS FROM *PIPER* SPP. Flavour Fragr J. 2003 May;18(3):198–201.

301. Vila R, Tomi F, Mundina M, Santana AI, Solís PN, López Arce JB, et al. Unusual composition of the essential oils from the leaves of *Piper aduncum*: UNUSUAL COMPOSITION OF ESSENTIAL OILS. Flavour Fragr J. 2005 Jan;20(1):67–9.

302. Vourlioti-Arapi F, Michaelakis A, Evergetis E, Koliopoulos G, Haroutounian SA. Essential oils of indigenous in Greece six Juniperus taxa: Chemical composition and larvicidal activity against the West Nile virus vector *Culex pipiens*. Parasitol Res. 2012 May;110(5):1829–39.

303. Vunda SLL, Sauter IP, Cibulski SP, Roehe PM, Bordignon SAL, Rott MB, et al. Chemical composition and amoebicidal activity of *Croton pallidulus*, *Croton ericoides*, and *Croton isabelli* (Euphorbiaceae) essential oils. Parasitol Res. 2012 Sep;111(3):961–6.

Supplementary material

304. Wong KC, Lim TB, Ali DMH. Essential oil of *Homalomena sagittifolia* Jungh. Flavour Fragr J. 2006 Sep;21(5):786–8.

305. Wong KC, Sivasothy Y, Boey PL. Essential oil of *Elettariopsis elan* C.K. Lim. Flavour Fragr J. 2006 May;21(3):562–4.

306. Xiao Z, Chen J, Niu Y, Chen F. Characterization of the key odorants of fennel essential oils of different regions using GC–MS and GC–O combined with partial least squares regression. Journal of Chromatography B. 2017 Sep;1063:226–34.

307. Xie F, Rizvi SAH, Zeng X. Fumigant toxicity and biochemical properties of (α + β) thujone and 1, 8-cineole derived from *Seriphidium brevifolium* volatile oil against the red imported fire ant *Solenopsis invicta* (Hymenoptera: Formicidae). Revista Brasileira de Farmacognosia. 2019 Nov;29(6):720–7.

308. Xing X, Ma JH, Fu Y, Zhao H, Ye XX, Han Z, et al. Essential oil extracted from *Erythrina corallodendron* L. leaves inhibits the proliferation, migration, and invasion of breast cancer cells. Medicine. 2019 Sep;98(36):e17009.

309. Xu T, Gherib M, Bekhechi C, Atik-Bekkara F, Casabianca H, Tomi F, et al. Thymyl esters derivatives and a new natural product modhephanone from *Pulicaria mauritanica* Coss . (Asteraceae) root oil: Thymyl esters and modhephanone from *Pulicaria mauritanica* root oil. Flavour Fragr J. 2015 Jan;30(1):83–90.

310. Yahyaa M, Tholl D, Cormier G, Jensen R, Simon PW, Ibdah M. Identification and Characterization of Terpene Synthases Potentially Involved in the Formation of Volatile Terpenes in Carrot (*Daucus carota* L.) Roots. J Agric Food Chem. 2015 May 20;63(19):4870–8.

311. Yang YC, Lee SH, Lee WJ, Choi DH, Ahn YJ. Ovicidal and Adulticidal Effects of *Eugenia caryophyllata* Bud and Leaf Oil Compounds on *Pediculus capitis*. J Agric Food Chem. 2003 Aug 1;51(17):4884–8.

312. Yapi TA, Boti JB, Ahibo AC, Bighelli A, Casanova J, Tomi F. Combined analysis of *Xylopia rubescens* Oliv. leaf oil using gas chromatography with flame ionization detection, gas chromatography with mass spectrometry and ^{13}C nuclear magnetic resonance: structure elucidation of new compounds: Structure elucidation of new compounds from *Xylopia rubescens* oil. Flavour Fragr J. 2013 Nov;28(6):373–9.

313. Yüce E, Paksoy MY, Bagci E. Essential Oil Composition of Two *Grammosciadium* DC Species, *G. platycarpum* (Boiss et Hausskn) Schischk and *G. macrodon* Boiss (Apiaceae), from Turkey. Trop J Pharm Res. 2016 Mar 4;15(2):411.

314. Zaouali Y, Bouzaine T, Boussaid M. Essential oils composition in two *Rosmarinus officinalis* L. varieties and incidence for antimicrobial and antioxidant activities. Food and Chemical Toxicology. 2010 Nov;48(11):3144–52.

315. Zeng WC, Zhu RX, Jia LR, Gao H, Zheng Y, Sun Q. Chemical composition, antimicrobial and antioxidant activities of essential oil from *Gnaphlium affine*. Food and Chemical Toxicology. 2011 Jun;49(6):1322–8.

Supplementary material

316. Zheljazkov VD, Cantrell CL, Tekwani B, Khan SI. Content, Composition, and Bioactivity of the Essential Oils of Three Basil Genotypes as a Function of Harvesting. J Agric Food Chem. 2008 Jan 1;56(2):380–5.

317. Zheljazkov VD, Maggi F. Valorization of CBD-hemp through distillation to provide essential oil and improved cannabinoids profile. Sci Rep. 2021 Oct 6;11(1):19890.

318. Zheljazkov VD, Noller JS, Maggi F, Dale R. Terpenes and Cannabinoids Yields and Profile from Direct-Seeded and Transplanted CBD- *Cannabis sativa*. J Agric Food Chem. 2022 Aug 31;70(34):10417–28.

319. Zini CA, Zanin KD, Christensen E, Caramão EB, Pawliszyn J. Solid-Phase Microextraction of Volatile Compounds from the Chopped Leaves of Three Species of *Eucalyptus*. J Agric Food Chem. 2003 Apr 1;51(9):2679–86.

320. Schmidt JM, Noletto JA, Vogler B, Setzer WN. Abaco Bush Medicine: Chemical Composition of the Essential Oils of Four Aromatic Medicinal Plants from Abaco Island, Bahamas. Journal of Herbs, Spices & Medicinal Plants. 2007;12(3):43–65.

Supplementary material

Additional file 1
Search strategies and results

Table S1: Summary of Databases Searched

Table	Vendor/ Interface	Database	Date searched	Database update	Searcher(s)
1a	Ovid	MEDLINE	14/07/2023	1946 to July 13 2023	N. Dalavaye; M. Nicholas; M. Pillai
1b	National Library of Medicine	PubMed	14/07/2023	July 13 2023	N. Dalavaye; M. Nicholas; M. Pillai
1c	Ovid	EMBASE	14/07/2023	1947 to July 13 2023	N. Dalavaye; M. Nicholas; M. Pillai

Supplementary material

Table S1a: Ovid MEDLINE search strategy

**Provider/Interface**	Ovid
**Database**	MEDLINE
**Date searched**	14/07/2023
**Database update**	1946 to July 13 2023
**Search developer(s)**	S. Erridge
**Limit to English**	No
**Date range**	1946–2023

1	Humulene.mp. [mp=ti, ab, hw, tn, ot, dm, mf, dv, kf, fx, dq, nm, ox, px, rx, an, ui, sy]
2	Alpha-Humulene.mp. [mp=ti, ab, hw, tn, ot, dm, mf, dv, kf, fx, dq, nm, ox, px, rx, an, ui, sy]
3	Alpha-Caryophyllene.mp. [mp=ti, ab, hw, tn, ot, dm, mf, dv, kf, fx, dq, nm, ox, px, rx, an, ui, sy]
4	1 or 2 or 3

Supplementary material

S1b: PubMed search strategy

**Provider/Interface**	National Library of Medicine
**Database**	PubMed
**Date searched**	14/07/2023
**Database update**	13/07/2023
**Search developer(s)**	S. Erridge
**Limit to English**	No
**Date range**	-13/07/2023

1	Humulene
2	Alpha-Humulene
3	Alpha-Caryophyllene
4	1 OR 2 OR 3

Supplementary material

S1c: Ovid EMBASE search strategy

**Provider/Interface**	Ovid
**Database**	EMBASE
**Date searched**	14/07/2023
**Database update**	1947 to July 13 2023
**Search developer(s)**	S. Erridge
**Limit to English**	No
**Date range**	1947–2023

1	Humulene.mp. [mp=ti, ab, hw, tn, ot, dm, mf, dv, kf, fx, dq, nm, ox, px, rx, an, ui, sy]
2	Alpha-Humulene.mp. [mp=ti, ab, hw, tn, ot, dm, mf, dv, kf, fx, dq, nm, ox, px, rx, an, ui, sy]
3	Alpha-Caryophyllene.mp. [mp=ti, ab, hw, tn, ot, dm, mf, dv, kf, fx, dq, nm, ox, px, rx, an, ui, sy]
4	1 or 2 or 3

Supplementary material

Supplementary Table 2: Overview of extraction of α-humulene by included studies

Organism	Chemovar	Extraction	Isolation	Yield	Reference
Acalypha plicata Müll-Arg.	Venezuela	Hydrodistillation in a Clevenger-type apparatus for 5 h	GC-MS	1.20%	10.1002/ffj.1679
Achillea lingulata	Serbia	Hydrodistillation in a Clevenger-type apparatus for 2.5 h	GC-MS	0.48%	10.1002/ffj.1778
Achillea mbellata	Greece	Hydrodistillation in a Clevenger-type apparatus for 2.5 h	GC-MS	0.04%	10.1002/ffj.1778
Acinos arvensis (Lam.) Dandy	Serbia	Hydrodistillation for 2.5 h using a Clevenger-type apparatus	GC-MS	0.70%	10.1002/ffj.1409
Acritopappus confertus	Brazil	Hydrodistillation using a Clevenger-type apparatus modified by Gottlieb for 3 hours	GC-MS	1.30%	10.1002/ffj.1483
Acroptilon repens (L.) DC. (Russian knapweed)	Iran	Hydrodistillation using a Clevenger-type apparatus	GC-MS	1.00%	10.1002/ffj.1568
Aethionema sancakense	Turkey	Hydrodistillation using a Clevenger-type apparatus	GC-MS	19.8%	10.3390/molecules27186129
Aframomum corrorima	Ethiopia	Steam distillation	GC-MS	0.1% (seeds) 1.1% (husks)	10.1002/ffj.1634
Aframomum exscapum (Sims) hepper	Guadeloupe	Hydrodistillation using a Clevenger-type apparatus for 10 h	GC-MS	0.1% (fruit pulp), 0.4% (stems), nil (leaves), nil (seeds)	10.1002/ffj.1741
Aframomum giganteum	Gabon	Hydrodistillation	GC-MS	0.2% (leaves) 0.6% (rhizomes)	10.1002/ffj.1403
Aframomum melegueta	France	Commercial (hexane:ethyl acetate extract), supercritical fluid extraction product (carbon dioxide extract)	GC-MS	10.5% [commercial], 7.2% [supercritical fluid extraction product]	10.1002/ffj.1554
Aframomum melegueta (Roscoe) K. Schum. (alligator pepper)	Nigeria	Hydrodistillation for 3h	Fractionation	60.90%	10.1002/%28SICI%291099-1026%28199903/04%2914:2%3C109::AID-FFJ775%3E3.0.CO;2-M
Ageratum fastigiatum	Brazil	Hydrodistillation according to Method I of the Brazilian Pharmacopeia, 5th Edition (2010) for 4 h	GC-MS	3.52%	10.1016/j.bjp.2015.03.002
Alpinia zerumbet	Japan	Hydrodistillation using a Clevenger-type apparatus	GC-MS	2.0 g/l (leaves)	10.1002/ffj.2047
	Brazil	Hydrodistillation using a Clevenger-type apparatus	GC-MS	0.29%	10.1590/1983-084X/15_054

Supplementary material

Anemia tomentosa var.anthriscifolia	Argentina	Hydrodistillation in a Clevenger-type apparatus	GC-MS	0.20%	10.1002/ffj.1341 Juliani
Annona leptopetala	Brazil	Hydrodistillation using a Clevenger-type apparatus for 4 h	GC-MS	1.32%	10.1016/j.bjp.2018.06.009
Anthemis triumfetti (Asteraceae)	NR	Hydrodistillation using a Clevenger-type apparatus	GC-MS	1.60%	10.1002/ffj.1592
Artabotrys jollyanus	Cote d'Ivoire	Hydrodistillation in a Clevenger type apparatus	GC-MS	3.00%	10.1016/j.bjp.2017.04.001 Goore
Artemisia scoparia Waldst. & Kit	India	Hydrodistillation according to the method recommended by the British Pharmacopoeia, 1988.	GC-MS	0.30%	10.1002/ffj.1278
Artemisia scoparia Waldst. et Kit	Turkey	Hydrodistillation using a Clevenger-type apparatus for 3 h	GC-MS	0.70%	10.1002/ffj.1426
Artemisia spicigera C. Koch	Turkey	Hydrodistillation using a Clevenger-type apparatus for 3 h	GC-MS	nil	10.1002/ffj.1426
Atlantia sessiflorawere	Vietnam	Hydrodistillation using a Clevenger apparatus for 3.5 hours	GC-MS	8.02+0.05%	10.3855/JIDC.12469
Baccharis trimera Less	Brazil	Commercial	GC-FID	3.10%	10.4314/tjpr.v14i11.19
Baeckea frutescens	Vietnam	Hydrodistillation using a Clevenger-type apparatus	GC-MS	5.80%	10.1016/j.jchromb.2006.11.042
Baeckea frutescens L	Malaysia	Hydrodistillation for 8 hours. Separated and dried over anhydrous magnesium sulphate	GC-MS	10.6% (coastal sample)	10.1002/%28SICI%291099-1026%281998070%2913:4%3C245::AID-FFJ736%3E3.0.CO;2-J
Blumea lacera	Vietnam	Hydrodistillation using a Clevenger-type apparatus	GC-MS	3.7% (flower), 3.5% (leaf), 1.5% (stem)	10.3390/molecules27227961
Blumea sinuata	Vietnam	Hydrodistillation using a Clevenger-type apparatus	GC-MS	4.3%	10.3390/molecules27227961
Boesenbergia stenophylla R. M. Sm	Malaysia	Hydrodistillation using a Clevenger-type apparatus for 8 h	GC-MS	5.3% (leaf), 2.8% (rhizone)	10.1002/ffj.1227
Bubonium graveolens	Algeria	Hydrodistillation using a Clevenger-type apparatus for 6 h	GC-MS	2.1% (leaves), 1.9% (flower)	10.1002/ffj.1794
Buddleja tucumanensis	Bolivia	Hydrodistillation with a Clevenger-type apparatus	GC-MS	1.10%	10.1002/ffj.1526 Lorenzo
Bupleurum gibraltarium	Spain	Hydrodistillation using a Clevenger-type apparatus for 8 h	GC-MS	0.40%	10.1021/jf040219n
C. japonicus (an insect, collected form the twigs of *Podocarpus nagi*)	Japan	Macerated in 10 ml of pentane	GC-MS	35.80%	https://dx.doi.org/10.1080/09168451.2020.1763156
C. obtusa var. formosana	Taiwan	Hydrodistillation using a Clevenger-type apparatus	GC-MS, GC-FID	0.30%	10.1002/ffj.1685

Supplementary material

Calamintha sylvatica Bromf. Subs. Sylvatica	Serbia	Hydrodistillation for 3 h using a Clevenger-type apparatus	GC-MS	0.2% (pre-blossom) 0.6% (full blossom) 0.8% (post-blossom)	10.1002/ffj.995
Calendula officinalis L.	Bosnia	Hydrodistillation	GC-MS, GC-FID	1.9% (leaves) 1.3% (flowers)	10.1002/ffj.3661
Callicarpa americana	Mississippi	Hydrodistillation using a Clevenger-type apparatus	GC-MS	10.00%	10.1016/j.jchromb.2006.11.045
Callitris intratropica	Nigeria	Hydrodistillation using a Clevenger-type apparatus	GC-MS	0.40%	10.1002/ffj.1214
Calocedrus formosana	Taiwan	Hydrodistillation using a Clevenger-type apparatus	GC-MS, GC-FID	0.40%	10.1002/ffj.1685
Calycorectes australis	Brazil	Hydrodistillation using a Clevenger-type apparatus for 5 h	GC-MS, GC-FID	1%	10.1002/ffj.1640
Calycorectes psidiiflorus	Brazil	Hydrodistillation using a Clevenger-type apparatus for 5 h	GC-MS, GC-FID	1%	10.1002/ffj.1640
Cannabis sativa L	Argentina (Cepas Argentinas Terapéuticas)	Headspace extraction with NaCL at 90°C	GC-FID	0.0059-0.0071 mg/g	https://doi.org/10.1016/j.chroma.2022.463669
	France	Commercial	GC-MS	8.71%	10.1002/ffj.993
	Poland (Henola variety; fibre type)	Ethanolic extract filtered through a Millipore filter	GC-FID	0.206-0.534 mg/g (fast GC-FID); 0.138-0.531 mg/g (conventional GC-FID)	10.1002/jssc.201900822
	United States (Culver cultivar)	Steam distillation	GC-MS	7.365% (30 mins distillation of dioecious, densely seeded system); 7.336% (240 mins of distillation of dioecious, densely seeded system); 2.59% (30 mins distillation of open, all-female, clonal transplant system); 4.23% (240 mins of distillation of open, all-female, clonal transplant system)	https://doi.org/10.1021/acs.jafc.1c06912?urlappend=%3Fref%3DPDF&jav=VoR&rel=cite-as
	United States	Non-stop steam distillation	GC-MS	9.1% (chopped autoflower type hemp t&h)	https://dx.doi.org/10.1038/s41598-021-99335-4
Capparis spinosa var. aegyptia (Lam.) Boiss	Egypt	Hydrodistillation using a Clevenger-type apparatus for 3 h	GC-MS	4.24%	10.1016/j.bjp.2016.04.001
Carum copticum	Iran	Extracted with equal volumes redistilled dichloromethane	GC-MS	2.01%	10.1002/ffj.1129 Lockwood
Cedrela fissilis	Brazil	Hydrodistillation in a Clevenger-type apparatus for 4 h	GC-MS	4.9% (leaf) 1.2% (stem bark)	10.1002/ffj.1347

Supplementary material

Cedrelopsis grevei H. Baillon	Madagascar	Commercial	GC-MS	0.8-5.4%	10.1002/ffj.1263
Centaurea calcitrapa L. (C.c.)	Italy	Hydrodistillation using a Clevenger-type apparatus	GC-MS	0.40%	10.1002/ffj.1585
	Poland	Solid-phase microextraction	GC-MS	9.77%	10.3390/molecules27041371
Centaurea huber-morathii Wagenitz	Turkey	Plant material was placed in an Eppendorf Microdistiller sample vial together with water. n-Hexane (0.3 ml) was added to the collecting vial to trap volatile compounds	GC-MS	0.30%	10.1002/ffj.1620
Centaurea sphaerocephala L. ssp. sphaerocephala (C.s.)	Italy	Hydrodistillation using a Clevenger-type apparatus	GC-MS	0.70%	10.1002/ffj.1585
Centella asiatica (L.) Urban (Family: Apiaceae)	South Africa	Leaf powder soaked in 1 L of methanol with continuous stirring for 72 h	GC-MS	1.25%	10.1016/j.biopha.2018.02.115
Ceroplastes rubens (an insect, collected form the twigs of *Podocarpus nagi*)	Japan	Macerated into 10 ml of pentane	GC-MS	3.90%	https://dx.doi.org/10.1080/09168451.2020.1763156
Chaerophyllum aksekiense	Turkey	Hydrodistillation using a Clevenger-type apparatus for 3 h	GC-MS	5.50%	10.1002/(SICI)1099-1026(200001/02)15:1<43::AID-FFJ864>3.0.CO;2-%23
Chaetomium globosum	N/A	Dried ethyl acetate extract of the liquid culture filtrate	GC-MS	1.60%	10.1016/j.biopha.2017.10.120
Chamaecyparis formosensis	Taiwan	Hydrodistillation using a Clevenger-type apparatus	GC-MS, GC-FID	2%	10.1002/ffj.1685
Chamomilla recutita L. Rausch	India	Hydrodistillation using a Clevenger-type apparatus	GC-MS	nr	10.1002/ffj.1035
Chloroxylon swietenia DC.	India	Hydrodistillation using a Clevenger-type apparatus	GC-MS	0.46% (leaves)	10.1007/s00436-007-0485-z
Cinnamomum camphora	Mauritius	NR	GC-MS	1.00%	10.1002/cbdv.202000921
Cinnamomum jensenianum	China	Plant material soaked in distilled water, extracted with volatile oil extractor	GC-MS	0.26%	10.4314/tjpr.v17i9.23
Cinnamomum rhyncophyllum Miq.	Malaysia	Hydrodistillation using a Clevenger-type apparatus for 8 h	GC-MS	1.1% (leaf), 0.1% (bark), nil (wood)	10.1002/ffj.1301
Cinnamomum tamala Nees et Eberm.	India	Hydrodistillation method recommended by the British Pharmacopoeia	GC-MS	0.20%	10.1002/ffj.1236
Cirsium japonicum DC	Japan	Hydrodistillation in a Likens–Nickerson-type apparatus	GC-MS	0.60% (rhizomes)	10.1002/ffj.1135
Citrus	France	Hydrodistillation using a Clevenger-type apparatus	GC-MS	0.1-0.2%	10.1002/ffj.1658
Citrus aurantium L.	West Indies	Rasping fresh bitter orange peels + cold pressing	GC-MS, GC-FID	0.01%	10.1002/ffj.2087
Citrus limon (L.)	Algeria	Hydrodistillation using a Clevenger-	GC-FID	0.04%	10.1002/ffj.1829

Supplementary material

		type apparatus for 3 h			
Citrus medical. Cv. Diamante	Italy	Syringe aspiration	GC-MS, GC-FID	0.06% (peel) 0.04% (rind)	10.1002/jssc.200800404
Clinopodium nepeta L	Turkey	Homogenised plant item was extracted with 250 ml extraction solvent (methanol) for 24 hours	GC-MS	0.10%	10.1002/ffj.3636
Clusia lanceolata	Brazil	Hydrodistillation using a Clevenger-type apparatus for 2 h	GC-MS	8.42% (galled leaves), 8.941% (non-galled leaves)	10.1016/j.bjp.2014.11.005
Conium maculatum L.	Iran	Hydrodistillation using a Clevenger-type apparatus for 3 h	GC-MS	1.40%	10.1002/ffj.1722
Conyza sumatrensis	Côte d'Ivoire	Hydrodistillation using a Clevenger-type apparatus for 3 h	GC-MS	1-1.4% (leaves), 1.9-2.4% (flower), 0.2% (roots)	10.1002/ffj.1743
Copaifera duckei oleoresin	Brazil	Commercial	GC-MS, GC-FID	2%	10.1016/j.bjp.2018.09.004
Copaifera langsdorffii Desf., Fabaceae,	Brazil	Macerated for 72 h with 70% aqueous ethanol. Filtered and concentrated under reduced pressure	GC-MS	Major component	10.1016/j.bjp.2015.05.005
Copaifera multijuga	Brazil	Distilled 4 h in distillation column and a serpentine condenser	GC-MS	10.20%	https://dx.doi.org/10.1590/S0102-695X2013005000038
Cordia verbenacea	Turkey	Steam distillation for 1.5 to 2 h	GC-MS, GC-FID	1.23%	10.1016/j.biopha.2019.108693
	Brazil	NR	HPLC	2.90%	10.1016/j.bjp.2019.01.009
	Brazil	Supercritical fluid extraction; Soxhlet extraction for 6h	GC-MS	2.10% (SFE) 1.10% (Soxhlet)	10.1016/j.biortech.2009.07.061
Croton ericoides	Rio, Brazil	Hydrodistillation using a Clevenger-type apparatus	GC-MS	1.10%	10.1007/s00436-012-2918-6
Croton isabelli	Rio, Brazil	Hydrodistillation using a Clevenger-type apparatus	GC-MS	2.30%	10.1007/s00436-012-2918-6
Croton pallidulus	Rio, Brazil	Hydrodistillation using a Clevenger-type apparatus	GC-MS	2.20%	10.1007/s00436-012-2918-6
Croton sellowii Baill (shrub)	NR	Maceration with acetone. Solvent removed under vacuum	GC-MS, GC-FID	0.8% (leaves)	10.1002/ffj.1298
Croton zambesicus	Benin	Hydrodistillation using a Clevenger-type apparatus for 4 h	GC-MS, GC-FID	1.60%	10.1002/ffj.1558
	Cameroon	Hydrodistillation using a Clevenger-type apparatus for 12 h	GC-MS	2.2% (leaves), 2% (rootbark), 2.3% (stembark)	10.1002/ffj.1081

Supplementary material

Cunninghamia lanceolata var. konishii	Taiwan	Hydrodistillation using a Clevenger-type apparatus	GC-MS, GC-FID	0.50%	10.1002/ffj.1685
Cupressus sempervirens ssp. Pyramidalis L.	NR	Hydrodistillation using a Clevenger-type apparatus	GC-MS	0.4% (leaves) trace (cones)	10.1111/j.1365-2184.2008.00561.x
Cupriavidus necator	Germany	20% n-dodecane	GC-MS	2-10 mg/l	https://doi.org/10.3390%2Fmolecules27248684
Curcuma angustifolia	India	Hydrodistillation using a Clevenger-type apparatus	GC-MS	0.30%	10.1002/ffj.1680
Curcuma longa L	India	Hydrodistillation using a Clevenger-type apparatus for 4.5 h	GC-MS	0.1 - 0.3%	10.1002/ffj.1780
Cyperus fuscus L	Turkey	Hydrodistillation using a Clevenger-type apparatus	GC-MS, GC-FID	0.60%	10.4314/tjpr.v17i8.24
Daucus carota L	Israel	Solid-phase microextraction device extraction	GC-MS	124.47 ng/g	https://dx.doi.org/10.1021/acs.jafc.5b00546
	Denmark	Dynamic headspace sampling with nitrogen	GC-MS	Cultivars (brasilia -1200 , duke -740 , fancy- 1610, and cortez - 2540) ng/50 g	10.1021/jf010213n
	Denmark	Dynamic headspace sampling	GC-MS	294 ng/g (refrigerated (1 °C)) 64 ng/g (frozen (-24°C))	10.1021/jf030212q
Daucus reboudii Coss.	Algeria	Hydrodistillation using a Clevenger-type apparatus	GC-MS, GC-FID	0.10%	10.1002/ffj.1636
Dianthus caryophyllus	Greece	Steam distillation for 4 h in a modified Clevenger distillation apparatus	GC-MS	1.90%	10.1007/s00436-012-3097-1
Dipteryx alata Vogel, Fabaceae	Brazil	Manual hydraulic pressing and mechanical continuous pressing	GC-MS	0.08% (hydraulic pressing) nil (continuous screw pressing)	10.1016/j.bjp.2015.07.019
Dorema ammoniacum	NR	Steam-distillation method via Clevenger apparatus	GC-MS	4.25%	https://doi.org/10.1155%2F2022%2F9725244
Dorema aucheri Boiss., Seseli	Iran	Hydrodistillation using a Clevenger-type apparatus for 3 h	GC-MS	0.20%	10.1002/ffj.1722
Doronicum corsicum	France	Hydrodistillation using a Clevenger-type apparatus	GC-MS	2.40%	10.1002/ffj.1824
Dryobalanops aromatica	Malaysia	Fractional distillation in the presence of double distilled water for 2 h	GC-MS	16.31%	10.4314/tjpr.v15i6.23
Echinacea angustifolia	Iran	Hydrodistillation for 3 h using a Clevenger-type apparatus	GC-MS	2.80%	10.1002/ffj.1657
Echinacea pallida	Iran	Hydrodistillation for 3 h using a	GC-MS	1.50%	10.1002/ffj.1657

Supplementary material

		Clevenger-type apparatus.			
Echinacea purpurea	Iran	Hydrodistillation for 3 h using a Clevenger-type apparatus.	GC-MS	1.50%	10.1002/ffj.1657
Elettariopsis elan C.K. Lim	Malaysia	Hydrodistillation using a Clevenger-type apparatus	GC-MS	0.1% (leaves), 0.2% (rhizomes), 0.2% roots	10.1002/ffj.1654
Emilia sonchifolia	Vietnam	Hydrodistillation using a Clevenger-type apparatus	GC-MS	2.8%	10.3390/molecules27227961
Eriocephalus africanus L.var. Africanus	Spain	Hydrodistillation for 3 h in a Clevenger-type apparatus	GC-MS	0.09±0.09% (burjassot) 0.03±0.04% (sagunto) 0.03±0.02% (valencia)	10.1002/ffj.1821
Eryngium yuccifolium Michaux	Germany	Hydrodistillation using n-pentane as a solvent for 6 h	GC-MS	0.60%	10.1002/ffj.1631
Erythrina corallodendron L.	China	Hydrodistillation using a Clevenger-type apparatus	GC-MS	1.57%	10.1097/MD.0000000000017009
Eucalyptus (*E.*) *dunnii*	Brazil	Headspace solid-phase microextraction	CG- ion-trap MS	NR (predicted 1-10%)	10.1021/jf026047g
Eucalyptus citriodora	Brazil	Headspace solid-phase microextraction	CG- ion-trap MS	Nil	10.1021/jf026047g
	India	Hydrodistillation for 3 h using a Clevenger-type apparatus	GC-MS	0.6g/100g	10.1002/ffj.3296
Eucalyptus saligna	Brazil	Headspace solid-phase microextraction	CG- ion-trap MS	nil	10.1021/jf026047g
Eugenia caryophyllata	South Korea	NR	GC-MS, GC-FID	0.8% (bud oil) 3.4% (leaf oil)	10.1021/jf034225f
	China	Solvent-free microwave extraction and hydrodistillation	GC-MS	3.09% (hydrodistillation) 5.06% (solvent free microwave extraction)	10.1002/jssc.201000148
Eugenia caryophyllus	Germany	NR	GC-MS	2.10%	10.1021/jf060608c
Euphorbia convolvuloides	Ivory coast	Hydrodistillation using a Clevenger-type apparatus	GC-MS, GC-FID	1.7% (aerial plant parts)	https://dx.doi.org/10.1002/ffj.3624
Euphorbia acanthothamnos	Greece	Dichloromethane extract	GC-MS	nil	10.1002/ffj.1148
Euphorbia apios	Greece	Dichloromethane extract	GC-MS	0.60%	10.1002/ffj.1148
Euphorbia characias	Greece	Dichloromethane extract	GC-MS	nil	10.1002/ffj.1148
Euphorbia dendroides	Greece	Dichloromethane extract	GC-MS	1.10%	10.1002/ffj.1148
Euphorbia helioscopia	Greece	Dichloromethane extract	GC-MS	0.40%	10.1002/ffj.1148
Euphorbia heterophylla	Ivory Coast	Hydrodistillation using a Clevenger-type apparatus	GC-MS, GC-FID	1.5% (aerial plant parts)	https://dx.doi.org/10.1002/ffj.3624

Supplementary material

Euphorbia hirta	Ivory Coast	Hydrodistillation using a Clevenger-type apparatus	GC-MS, GC-FID	1.4% (aerial plant parts)	https://dx.doi.org/10.1002/ffj.3624
Euphorbia rigida	Greece	Dichloromethane extract	GC-MS	0.70%	10.1002/ffj.1148
Ferulago campestris (Apiaceae)	Italy	Hydrodistillation in a Clevenger-type apparatus for 4 h	GC-MS GC-FID	1.6 ±0.14% (flowers) 5.1 ±0.52% (leaves)	10.1002/ffj.1941
Ferulago campestris (Besser) Grecescu	Italy	Hydrodistillation using a Clevenger-type apparatus for 3 h	GC-MS, GC-FID	0.6-0.7%	10.1002/ffj.2010
Foeniculum vulgare Mill (Fennel)	China	Hydrodistillation using a Clevenger-type apparatus	GC-MS	0.07%	https://dx.doi.org/10.1016/j.jchromb.2017.07.053
Galeopsis pubescens	Italy	Hydrodistillation using a Clevenger-type apparatus for 2 h	GC-MS, GC-FID	0.80%	10.1002/ffj.1307
Galeopsis tetrahit	Italy	Hydrodistillation using a Clevenger-type apparatus for 2 h	GC-MS, GC-FID	0.30%	10.1002/ffj.1307
Garcinia atroviridis Griff. Ex T. Anders (Clusiaceae)	Malaysia	Hydrodistillation using a Clevenger-type apparatus	GC-MS	10.70%	10.1016/j.jchromb.2006.11.043
Garcinia huillensis Welw. ex. Oliv.	Zimbabwe	Hydrodistillation using a Clevenger-type apparatus for 1.5 h	GC-MS	10.1-23%	10.1002/ffj.1420
Geniosporum rotundifolium Briq	Tanzania	Hydrodistillation using a Clevenger-type apparatus	GC-MS	0.53%	10.4314/tjpr.v15i1.15
Gnaphlium affine	China	Hydrodistillation using a Clevenger-type apparatus	GC-MS	3.22%	https://dx.doi.org/10.1016/j.fct.2011.03.014
Grammosciadium macrodon Boiss	Turkey	Hydrodistillation using a Clevenger-type apparatus	GC-MS	1%	10.4314/tjpr.v15i2.26
Grammosciadium platycarpum	Turkey	Hydrodistillation using a Clevenger-type apparatus	GC-MS	nil	10.4314/tjpr.v15i2.26
Guatteria juruensis	Brazil	Hydrodistillation for 4 h using a Clevenger-type apparatus	GC-MS	nil	10.1002/ffj.1500
Guatteria microcalyx	Brazil	Hydrodistillation for 4 h using a Clevenger-type apparatus	GC-MS	0.10%	10.1002/ffj.1500
Guatteria poeppigiana	Brazil	Hydrodistillation for 4 h using a Clevenger-type apparatus	GC-MS	trace	10.1002/ffj.1500
Gundelia. tournefortii (EOGT)	Zarka, Jordan	Hydrodistillation using a Clevenger-type apparatus	GC-MS, GC-FID	2.10%	10.4314/tjpr.v15i10.17
Gynura bicolor DC (Asteraceae - plants and shoots	Japan	Solvent-assisted flavour evaporation (SAFE) of solvent extracts	GC-MS	9.6% (plants), 11.6% (regenerates), 5.6% (cultured shoots)	10.1002/ffj.1938
Gynura bicolor DC (Asteraceae)- roots	Japan	Roots immersed in freshly distilled diethyl ether	GC-MS	8.1% (field grown roots), 12.3% (cultured)	10.1002/ffj.2016
Haumaniastrum villosum (Bene) AJ Paton (Lamiaceae)	Tanzania	Hydrodistillation using a Clevenger-type apparatus	GC-MS	5.63%	10.4314/tjpr.v15i1.15
Hedyosmum angustifolium	Bolivia	A Clevenger-type glass hydrodistillation apparatus	GC-MS	0.20%	10.1002/ffj.1146

Supplementary material

Helichrysum faradifani Sc. Ell.	Madagascar	Commercial	GC-MS	1.40%	10.1002/ffj.1531
Helichrysum kraussii Sch. Bip	South Africa	Steam distillation using a Clevenger-type apparatus for 3 h	GC-MS	9.80%	10.1002/ffj.1152
Helichrysum rugulosum Less	South Africa	Steam distillation using a Clevenger-type apparatus for 3 h	GC-MS	Nil	10.1002/ffj.1152
Heterothalamus alienus (Spreng.) Kuntze	Argentina	Hydrodistillation using a Clevenger-type apparatus for 3 h	GC-MS	1.6-2.1%	10.1002/ffj.1747
Hexachlamys edulis	Brazil	Hydrodistillation using a Clevenger-type apparatus for 5 h	GC-MS, GC-FID	8.00%	10.1002/ffj.1385
Hexachlamys hamiltonii	Brazil	Hydrodistillation using a Clevenger-type apparatus for 5 h	GC-MS, GC-FID	2.50%	10.1002/ffj.1385
Hexachlamys humilis	Brazil	Hydrodistillation using a Clevenger-type apparatus for 5 h	GC-MS, GC-FID	2.70%	10.1002/ffj.1385
Hexachlamys itatiaiensis	Brazil	Hydrodistillation using a Clevenger-type apparatus for 5 h	GC-MS, GC-FID	5.80%	10.1002/ffj.1385
Homalomena sagittifolia Jungh.	Malaysia	Hydrodistillation using a Clevenger-type apparatus	GC-MS, GC-FID	3.9% (leaves), 0.2% (rhizomes)	10.1002/ffj.1714
Hortia oreadica	Brazil	Hydrodistillation using a Clevenger-type apparatus	GC-MS	0.59%	10.1016/j.bjp.2015.08.008
Hoslundia opposita Vahl	Zimbabwe	Hydrodistillation using a Clevenger-type apparatus for 1.5 to 2 h	GC-MS	0.2-7.6%	10.1002/ffj.1402
	Ivory Coast	Hydrodistillation using a Clevenger-type apparatus	GC-MS	5.70%	10.1002/ffj.1715
Humulus lupulus L.	Brazil (Chinook variety)	Hydrodistillation using a Clevenger-type apparatus	GC-MS	31.50% (90 mins distillation); 32.63% (180 mins distillation); 34.62% (300 mins distillation)	https://doi.org/10.1007/s00284-023-03359-0
	Germany	Supercritical fluid carbon dioxide extraction	GC-MS	6.72%	10.1021/jf402496t
	Japan	Stir bar−sorptive extraction (SBSE) method	GC-MS	0.73%	10.1021/jf050072f
	Poland (Marynka and Magnum varieties)	Headspace extraction at 40°C for 20 mins	GC-MS	0.0032-0.0169mg/l	https://doi.org/10.3390%2Fmolecules27227910
	Portugal	Headspace solid-phase microextraction	GC-MS	16.6 ± 0.8%	10.1002/jssc.201200244
Hymenocrater incanus Bunge	Iran	Hydrodistillation using a Clevenger-type apparatus for 3.5 h	GC-MS	0.60%	10.1002/ffj.983
Hypericum brasiliense	Brazil	Hydrodistillation for 3 h	GC-MS	12.74%	10.1002/ffj.1319
Hypericum olympicum L	Greece	Hydrodistillation using a Clevenger-type apparatus	GC-MS, GC-FID	1.50%	10.1002/ffj.1521

Supplementary material

Hypericum perforatum L.	Greece	Hydrodistillation using a Clevenger-type apparatus	GC-MS, GC-FID	Trace	10.1002/ffj.1521
Hypericum tetrapterum Fries	Greece	Hydrodistillation using a Clevenger-type apparatus	GC-MS, GC-FID	Trace	10.1002/ffj.1521
Hyptis carpinifolia.	Brazil	Hydrodistillation using a Clevenger-type apparatus for 2 h	GC-MS	0.2-0.9%	10.1016/j.bjp.2016.05.011
Hyptis pectinata	Brazil	Hydrodistillation for 140 mins in Clevenger style apparatus	GC-MS	Room temperature storage 2.79% to 2.21% at 1 year;<br>freezer 2.79% to 2.43% at 1 year.	10.1590/1983-084X/15_177
Hyptis suaveolens (Lamiaceae)	Italy	Hydrodistillation using a Clevenger-type apparatus for 2 h	GC-MS	0.90%	10.1007/s00436-011-2730-8
Illicium verum	Greece	Steam distillation for 4 h in a modified Clevenger distillation apparatus	GC-MS	Nil	10.1007/s00436-012-3097-1
Inula graveolens	France	Commercial	GC-MS	0.20%	10.1002/ffj.1304
Isolona campanulata Engler & Diels	Côte-d'Ivoire	Hydrodistillation using a Clevenger-type apparatus for 3 h	GC-MS, GC-FID	10.40%	10.1002/ffj.1555
Isolona dewevrei	Cote d'Ivoire	Hydrodistillation for 3 h in a Clevenger-type apparatus	GC-MS	1.20%	Https://dx.doi.org/10.1002/ffj.3612 Kambire
J. drupacea Labill.	Greece	Hydrodistillation using a Clevenger-type apparatus	GC-MS	0.80%	10.1007/s00436-011-2706-8
J. foetidissima Willd.	Greece	Hydrodistillation using a Clevenger-type apparatus	GC-MS	Nil	10.1007/s00436-011-2706-8
J. oxycedrus L. ssp. macrocarpa	Greece	Hydrodistillation using a Clevenger-type apparatus	GC-MS	Nil	10.1007/s00436-011-2706-8
J. oxycedrus L. ssp. oxycedrus	Greece	Hydrodistillation using a Clevenger-type apparatus	GC-MS	0.44%	10.1007/s00436-011-2706-8
J. phoenicea L	Greece	Hydrodistillation using a Clevenger-type apparatus	GC-MS	1.01%	10.1007/s00436-011-2706-8
Juglans regia L	Czech Republic	Solvent extraction with a shaker	GC-MS	≈ 9%	10.1002/jssc.200700371
	Algeria	Microwave-assisted hydrodistillation for 1 h; hydrodistillation using a Clevenger-type apparatus for 3 h	GC-MS, GC-FID	15.64% (microwave-assisted hydrodistillation for 1 h), 8.08% (hydrodistillation using a Clevenger-type apparatus for 3 h)	10.1002/hlca.201200359
Juniperus communis	Croatia and Bosnia	Hydrodistillation using a Clevenger-type apparatus	GC-MS	2.40% (fruit)	Https://dx.doi.org/10.1002/ffj.3602
Juniperus communis var. saxatilis	Belgrade	Hydrodistillation using a Clevenger-type apparatus	GC-MS	3.08%	10.1016/j.fct.2017.12.044

Supplementary material

Juniperus communisl. Ssp. Nana	Italy	Supercritical CO_2 extractions and hydrodistillation: performed in a circulatory Clevenger-type apparatus for 5 h	GC-MS	Leaves: 0.8-2.7%; berries: 1.5-2.0%; wood: 2.8-4.9%	10.1002/ffj.1549
Juniperus deltoides	Croatia and Bosnia	Hydrodistillation using a Clevenger-type apparatus	GC-MS	0.90% (leaf)	Https://dx.doi.org/10.1002/ffj.3602
Juniperus drupacea	Greece	Steam distillation using a Clevenger apparatus for 3 h	GC-MS	0.99%	10.1007/s00436-016-4959-8
Juniperus macrocarpa	Croatia and Bosnia	Hydrodistillation using a Clevenger-type apparatus	GC-MS	1.30% (leaf)	Https://dx.doi.org/10.1002/ffj.3602
Juniperus oxycedrus	Croatia and Bosnia	Hydrodistillation using a Clevenger-type apparatus	GC-MS	1.3% (leaf)	Https://dx.doi.org/10.1002/ffj.3602
Juniperus oxycedrusssp.oxycedrus	France	Hydrodistillation using a Clevenger-type apparatus for 3 h	GC-MS	0.8-1.2% (berry oil), 0.2% (leaf oil)	10.1002/ffj.1579
Juniperus phoenica	Greece	Steam distillation using a Clevenger apparatus for 3 h	GC-MS	1.15%	10.1007/s00436-016-4959-9
Juniperus- *J. communis* L. ssp. hemisphaerica	Greece	Hydrodistillation using a Clevenger-type apparatus	GC-MS	0.44%	10.1007/s00436-011-2706-8
Kielmeyera rugosa	Brazil	Hydrodistillation using a Clevenger-type apparatus for 3 h	GC-MS	3 - 5%	10.1002/ffj.1751
Lantana camara L.	Congo	Hydrodistillation using a Clevenger-type apparatus	GC-MS	10.6% (leaves)	10.1002/ffj.1553
	Nigeria	Hydrodistillation using a Clevenger-type apparatus	GC-MS	19.5% (leaves)	10.1002/ffj.1206
	India	Hydrodistillation in a conventional Clevenger-type apparatus for 4 h	GC-MS	2.4% (fruit); 0.7% (stem); 2.7% (leaves); 2.7% (flowers)	10.1002/ffj.1197
	Iran	Hydrodistillation using a Clevenger-type apparatus for 4 h	GC-MS	6-10.8%	10.1002/ffj.1048
	NR	Hydrodistillation using a Clevenger-type apparatus	GC-MS, GC-FID	5.2% (pink flowers) 2.6% (yellow flowers)	10.1002/ffj.1239
	Brazil	Hydrodistillation using n-pentane and a Chrompak distillation apparatus	GC-MS	1.2-10.7% (leaves and thin branches), 9.5% (flowers)	10.1002/(SICI)1099-1026(199907/08)14:4<208::AID-FFJ811>3.0.CO;2-F
	South China	Hydrodistillation using a Clevenger-type apparatus	GC-MS	9.31%	10.1002/ffj.1292
	Vietnam	Hydrodistillation using a Clevenger-type apparatus	GC-MS	2.3-6.9%	https://doi.org/10.1002/cbdv.202100145
Lantana salvifolia Jacq. (Verbenaceae)	Congo	Hydrodistillation using a Clevenger-type apparatus	GC-MS	0.5% (leaves)	10.1002/ffj.1553
Lavandula angustifolia	Italy	Commercial	GC-MS, GC-FID	0.41%	10.1080/13693780400004810

Lavandula angustifolia x hybrida cultivars	Italy	Hydrodistillation with a Clevenger apparatus for 2 h	GC-MS	0.06% (*L. angustifolia*) hybrida cultivars: 0.25% (ordinario) nil (alardii) 0.11% (abrialis) 0.13% (r.c) 0.07% (super z)	10.1002/ffj.3145
Lepechinia conferta	Venezuela	Hydrodistillation using a Clevenger-type apparatus	GC-MS	0.70%	10.1002/ffj.1550
Lepidium sativum	Greece	Steam distillation for 4 h in a modified Clevenger distillation apparatus	GC-MS	Nil	10.1007/s00436-012-3097-1
Leptospermum amboinense	Australia	Hydrodistillation with cohobation	GC-MS	0.4 - 0.9%	10.1002/1099-1026(200009/10)15:5<342::AID-FFJ924>3.0.CO;2-V
Leptospermum brachyandrum (F. Muell.) Druce	Australia	Steam distillation with cohobation	GC-MS	9-18%	10.1002/(SICI)1099-1026(199801/02)13:1<19::AID-FFJ679>3.0.CO;2-9
Leptospermum emarginatum	Australia	Hydrodistillation with cohobation	GC-MS	0.10%	10.1002/1099-1026(200009/10)15:5<342::AID-FFJ924>3.0.CO;2-V
Leptospermum grandiflorum	Australia	Hydrodistillation with cohobation	GC-MS	0.6 - 0.8%	10.1002/1099-1026(200009/10)15:5<342::AID-FFJ924>3.0.CO;2-V
Leptospermum liversidgei	Australia	Hydrodistillation with cohobation	GC-MS	0.40%	10.1002/1099-1026(200009/10)15:5<342::AID-FFJ924>3.0.CO;2-V
Leptospermum luehmannii F. M. Bailey	Australia	Steam distillation with cohobation	GC-MS	3-5%	10.1002/(SICI)1099-1026(199801/02)13:1<19::AID-FFJ679>3.0.CO;2-9
Leptospermum madidum A. R. Bean subsp. madidum	Australia	Steam distillation with cohobation	GC-MS	4-11%	10.1002/(SICI)1099-1026(199801/02)13:1<19::AID-FFJ679>3.0.CO;2-9
Leptospermum madidum ssp.sativum	Australia	Hydrodistillation with incubation	GC-MS	2.30%	10.1002/1099-1026(200007/08)15:4<271::AID-FFJ910>3.0.CO;2-E
Leptospermum morrisonii	Australia	Hydrodistillation with incubation	GC-MS	0.60%	10.1002/1099-1026(200007/08)15:4<271::AID-FFJ910>3.0.CO;2-E
Leptospermum oreophilum	Australia	Hydrodistillation with incubation	GC-MS	1 - 2%	10.1002/1099-1026(200007/08)15:4<271::AID-FFJ910>3.0.CO;2-E
Leptospermum pallidum A. R. Bean	Australia	Steam distillation with cohobation	GC-MS	0.30%	10.1002/(SICI)1099-1026(199801/02)13:1<19::AID-FFJ679>3.0.CO;2-9
Leptospermum petersonii	Australia	Hydrodistillation with cohobation	GC-MS	0.40%	10.1002/1099-1026(200009/10)15:5<342::AID-FFJ924>3.0.CO;2-V
Leptospermum polygalifolium ssp. 'wallum'	Australia	Hydrodistillation with incubation	GC-MS	7-11%	10.1002/1099-1026(200007/08)15:4<271::AID-FFJ910>3.0.CO;2-E
Leptospermum polygalifolium ssp. howese	Australia	Hydrodistillation with incubation	GC-MS	0.20%	10.1002/1099-1026(200007/08)15:4<

Supplementary material

					271::AID-FFJ910>3.0.CO;2-E
Leptospermum polygalifolium ssp. montanum	Australia	Hydrodistillation with incubation	GC-MS	1.00%	10.1002/1099-1026(200007/08)15:4<271::AID-FFJ910>3.0.CO;2-E
Leptospermum polygalifolium ssp. polygalifolium	Australia	Hydrodistillation with incubation	GC-MS	0.10%	10.1002/1099-1026(200007/08)15:4<271::AID-FFJ910>3.0.CO;2-E
Leptospermum polygalifolium ssp. Transmontanum	Australia	Hydrodistillation with incubation	GC-MS	1.20%	10.1002/1099-1026(200007/08)15:4<271::AID-FFJ910>3.0.CO;2-E
Leptospermum polygalifolium ssp. tropicum	Australia	Hydrodistillation with incubation	GC-MS	Nil	10.1002/1099-1026(200007/08)15:4<271::AID-FFJ910>3.0.CO;2-E
Leptospermum polygalifolium ssp.cismontanum	Australia	Hydrodistillation with incubation	GC-MS	0.8-9%	10.1002/1099-1026(200007/08)15:4<271::AID-FFJ910>3.0.CO;2-E
Leptospermum purpurascens Joy Thomps	Australia	Steam distillation with cohobation	GC-MS	0.30%	10.1002/(SICI)1099-1026(199801/02)13:1<19::AID-FFJ679>3.0.CO;2-9
Leptospermum rotundifolium	Australia	Hydrodistillation with cohobation	GC-MS	0.20%	10.1002/1099-1026(200009/10)15:5<342::AID-FFJ924>3.0.CO;2-V
Leptospermum sp. (Mt Maroon A.R. Bean 6665)	Australia	Hydrodistillation with incubation	GC-MS	44-51%	10.1002/1099-1026(200007/08)15:4<271::AID-FFJ910>3.0.CO;2-E
Leptospermum speciosum Schauer	Australia	Steam distillation with cohobation	GC-MS	0.10%	10.1002/(SICI)1099-1026(199801/02)13:1<19::AID-FFJ679>3.0.CO;2-9
Leptospermum variabile	Australia	Hydrodistillation with incubation	GC-MS	11-22%	10.1002/1099-1026(200007/08)15:4<271::AID-FFJ910>3.0.CO;2-E
Leptospermum whitei Cheel	Australia	Steam distillation with cohobation	GC-MS	0.50%	10.1002/(SICI)1099-1026(199801/02)13:1<19::AID-FFJ679>3.0.CO;2-9
Leptospermum wooroonooran	Australia	Hydrodistillation with cohobation	GC-MS	11 - 20%	10.1002/1099-1026(200009/10)15:5<342::AID-FFJ924>3.0.CO;2-V
Libanotis W. D. Koch var. Armeniacum Bordz.	Iran	Hydrodistillation using a Clevenger-type apparatus for 3 h	GC-MS	Nil	10.1002/ffj.1722
Licuala grandis	Thailand	Dynamic headspace extraction	GC-MS	1.60%	10.1002/ffj.1797
Licuala lauterbachii	Thailand	Dynamic headspace extraction	GC-MS	Nil	10.1002/ffj.1797
Licuala mattanensis	Thailand	Dynamic headspace extraction	GC-MS	0.10%	10.1002/ffj.1797
Licuala spinosa	Thailand	Dynamic headspace extraction	GC-MS	Nil	10.1002/ffj.1797
Lippia adoensis	Nigeria	Hydrodistillation for 4 h	GC-MS	0.60%	10.1002/ffj.1234
Lippia alba	Guatemala	Hydrodistillation using a Clevenger-type apparatus for 1.5 h	GC-MS	1.10%	10.1002/ffj.1309

Supplementary material

Lippia alba (Mill.) N.E. Brown (Verbenaceae)	Colombia	Microwave-assisted hydrodistillation method	Chromatog GC-MS	nil	10.1590/S1415-47572011005000030
Lippia gracilis	Brazil	Hydrodistillation using a Clevenger-type apparatus for 140 mins	GC-MS	0.47% (LGRA-106), 1% (LGRA-108), 0.38% (LGRA-109), 0.49% (LGRA-201)	10.1016/j.vetpar.2012.12.046
Lippia Graveolens	NR	Water distillation in a Clevenger-type apparatus	GC-MS	1.60%	10.1007/s00436-010-1800-7
Lippia integrifolia	Argentina	Hydrodistillation using a Clevenger-type apparatus for 4 h	GC-MS	1.3-4.5%	10.1002/ffj.1736
Lippia javanica (Burm. f.)	Tanzania	Hydrodistillation using a Clevenger-type apparatus	GC-MS	1.40%	https://dx.doi.org/10.1002/ffj.3625
Liquidambar orientalis Mill.	Turkey	Hydrodistillation using a Clevenger-type apparatus for 4 h	GC-MS	0%	10.1002/ffj.1370
Liquidambar styraciflua	Honduras	Hydrodistillation using a Clevenger-type apparatus for 4 h	GC-MS	1.10%	10.1002/ffj.1370
Mandarina Bavaria hops	Germany	Headspace solid-phase microextraction	GC-MS	25 ± 9%	10.1021/acs.jafc.9b06139<br>Machado
Mangifera indica (mango fruit)	Colombia	Simultaneous distillation–extraction	GC-MS	0.90%	10.1002/ffj.1812
Pinus pinaster Ait	France	Hydrodistillation using a Clevenger-type apparatus	GC-MS	2.20%	10.1002/ffj.1865
Marsh white grapefruit	Florida	Fruit extract dissolved in 0.1 ml of methylene chloride	Capillary gas chromatography	0.03%	10.1021/jf981064k
Melaleuca alternifolia	Italy	Hydrodistillation using a Clevenger-type apparatus for 2 h	GC-MS	Nil	10.1007/s00436-013-3651-5
Melaleuca quinquenervia (Cav.) S. T. Blake	New Caledonia	Hydrodistillation using a Clevenger-type apparatus	GC-MS, GC-FID	0.21%	10.1002/ffj.1649
Melodorum fruticosum flowers	Thailand	modified Likens–Nickerson apparatus	GC-MS	0.18%	10.1016/j.fct.2010.07.002
Mentha avensis (corn mint)	India	Hydrodistillation using a Clevenger-type apparatus	GC-MS, GC-FID	<0.05%	10.1002/ffj.1417
Mentha suaveolens ssp. insularis	France	Hydrodistillation using a Clevenger-type apparatus	GC-MS	0.10%	10.1002/ffj.1863
Mentha x piperita L.	India	Hydrodistillation using a Clevenger-type apparatus	GC-MS	Nil	10.1002/ffj.1333
Meum athamanticum (L.) Jacq.,	Germany	Hydrodistillation using a Clevenger-type apparatus	GC-MS	0.10%	10.1016/j.jchromb.2006.11.046
Microglossa pyrifolia	Côte d'Ivoire	Hydrodistillation using a Clevenger-type apparatus for 3 h	GC-MS	27.1–36.4% (leaves), 1.4% (buds)	10.1002/ffj.1743
Miocene amber	India	Dichloromethane: methanol by ultrasonication for 20 mins	GC-MS	NR	10.1038/s41598-017-09385-w

Supplementary material

Monanthotaxis diclina (Sprague)	Congo (Zaire)	Steam distilled 3 h	Filtered over anhydrous sodium sulphate	0.2% (root) 6.9% (fruit)	10.1002/%28SICI%291099-1026%28199703%2912:2%3C95::AID-FFJ611%3E3.0.CO;2-Z
Mosla dianthera Maxim	Vietnam	Steam distillation for 1 h with distilled water	GC-MS	5.09%	Https://pubmed.ncbi.nlm.nih.gov/10898640/ Kim
Mosla soochowensis	China	Steam distillation	GC-MS	4.04%	10.4314/tjpr.v16i4.23
Murraya exotica	India	Hydro-distillation using the Clevenger X77 type of apparatus for 4 h	GC-MS	0.03%	https://dx.doi.org/10.1007/s00436-015-4370-x
Murraya paniculata (L.) Jack	Nigeria	Hydrodistillation using a Clevenger-type apparatus	GC-MS	5.10%	10.1002/ffj.1365
	India	Hydrodistillation using a Clevenger-type apparatus	GC-MS	0.80%	10.1002/ffj.1804
Myrciaria tenella	Brazil	Hydrodistillation using a Clevenger-type apparatus	GC-MS	2.3-5.3%	10.3390/molecules27072234
Myriactis nepalensis Less.	China	Hydrodistillation using a Clevenger-type apparatus for 3.5 h	GC-MS	3.2%	10.3390/molecules27144631
Myrrhinium atropurpureum	Brazil	Hydrodistillation using a Clevenger-type apparatus for 3 h	GC-MS	1.42%	10.1111/and.13074
Myrtus communis	Tunisia	Steam distillation	GC-MS	0.25% (flowering stage)	10.1002/ffj.1453
	Morocco	Continuous distillation	GC-MS	0.30%	10.1002/ffj.1651
Nectandra barbellata	Brazil	Hydrodistillation in a Clevenger apparatus for 3 h	Thin layer chromatography then GCMS	3.79%	10.1016/j.bjp.2017.11.008
Nepeta crassifolia Boiss	Iran	Hydrodistillation using a Clevenger-type apparatus for 6 h	GC-MS	nil	10.1002/ffj.1199
Nepeta glomerulosa Boiss. subsp. carmanica	Iran	Hydrodistillation using a Clevenger-type apparatus for 4 h	GC-MS	3.20%	10.1002/(SICI)1099-1026(199909/10)14:5<265::AID-FFJ822>3.0.CO;2-A
Nepeta italica L	Turkey	Homogenised plant item was extracted with 250 ml extraction solvent (methanol) for 24 hours	GC-MS	Nil	10.1002/ffj.3636
Nepeta macrosiphon Boiss.	Iran	Steam-distilled for 5 h using a Clevenger-type apparatus	GC-MS	0.60%	10.1002/ffj.1287
Nigella arvensis L	Czech Republic	Hydrodistillation in a Clevenger-type apparatus for 3 h	GC-MS	Trace	10.1002/ffj.1713

Supplementary material

Ocimum basilicum	Saudi Arabia	Hydrodistillation using a Clevenger-type apparatus for 4 h	GC-MS	0.93%	10.1007/s11011-017-0173-3
	West Lafayette, USA	Hydrodistillation using a Clevenger-type apparatus	GC-MS, GC-FID	11.50%	10.1002/ffj.1513
	Brazil	Steam distillation for 1 h	GC-MS	nil	10.1002/ffj.1134
Ocimum basilicum L. (sweet basil)	Germany; Mesten	Hydrodistillation using a Clevenger-type apparatus	GC-MS	0.52% German 0.67% Mesten	https://doi.org/10.1021/jf0725629
Ocimum basilicum. var. minimum	Brazil	Steam distillation for 1 h	GC-MS	nil	10.1002/ffj.1134
Ocimum basilicum. var. purpurascens Benth	Brazil	Steam distillation for 1 h	GC-MS	1.60%	10.1002/ffj.1134
Ocimum gratissimum	Brazil	Hydrodistillation using a Clevenger-type apparatus	GC-MS	0.20%	10.1007/s00436-017-5662-0
Ocimum sanctum	Mississippi	Hydrodistillation using a Clevenger-type apparatus	GC-MS	1.99%	https://doi.org/10.1021/jf0725629
Ocotea elegans	Brazil	Hydrodistillation in a Clevenger apparatus for 3 h	Thin layer chromatography then GCMS	nil	10.1016/j.bjp.2017.11.008
Ocotea indecora	Brazil	Hydrodistillation in a Clevenger apparatus for 3 h	Thin layer chromatography then GCMS	nil	10.1016/j.bjp.2017.11.008
Oplopanax horridus	Canada	Steam distillation	GC-MS	0.2% (stem) 0.1% (root)	10.1002/ffj.1716
Origanum compactum	Morocco	Hydrodistillation	GC-MS	0.22%	10.1016/j.mrgentox.2007.01.011
Origanum ehrenbergii Boiss	Lebanon	Cyclohexane, dichlormethane, ethyl acetate and methanol extracts	GC-MS	"Low presence" (cyclohexane extract), "low presence" (dichloromethane extract), nil (ethyl acetate extract), nil (methanol extract)	10.1002/ffj.3646
Origanum glandulosum Desf	Algeria	Hydrodistillation using a Clevenger-type apparatus	GC-MS	0.30%	10.1002/ffj.1738
Origanum majorana	Iran	Leaves were placed in a sealed glass vial for 30 min at room temperature with a nanofiber sheet above it to collect volatiles. The nanofiber sheet was folded and inserted inside a 5 ml glass vial for solvent desorption using 2 ml of hexane for 10 min and the organic extract was concentrated by a gentle flow of nitrogen up to 0.5 ml	GC-MS	0.17%	10.1002/jssc.201301355

Supplementary material

	Lithuania	Hydrodistillation using a Clevenger-type apparatus; simultaneous distillation–solvent extraction	GC-MS	0.2% (hydrodistillation using a Clevenger-type apparatus); 0.1% (simultaneous distillation–solvent extraction)	10.1002/ffj.1478
	Germany	Hydrodistillation using a Clevenger-type apparatus	GC-MS	0.20%	10.1002/ffj.1077
Origanum virens		Water distillation in a Clevenger-type apparatus	GC-MS	0.10%	10.1007/s00436-010-1800-7
Origanum vulgare	USA	Commercial	GC-MS, GC-FID	0.51%	10.1016/j.biopha.2018.10.028
Ostericum grosseserratum	China	Hydrodistillation using a Clevenger-type apparatus for 6 h	GC-MS	0.70%	10.4314/tjpr.v12i1.16
Otacanthus azureus	French Guyana	Hydrodistillation	GC-MS	10.56%	10.1111/jam.12377
Panax ginseng	Korea	Dichloromethane extract	GC-MS	5.5 - 6.4%	10.1021/jf301835v
Panax notoginseng	Korea	Dichloromethane extract	GC-MS	3.70%	10.1021/jf301835v
Panax quinquefolius	Korea	Dichloromethane extract	GC-MS	Nil	10.1021/jf301835v
Pangasius (*Pangasianodon hypophthalmus*)	Bangladesh	Dynamic headspace sampling method (terpenes in the flesh)	GC-MS	8.3 ng/g	10.1021/acs.jafc.7b00497
Parthenium hysterophorus	Vietnam	Hydrodistillation using a Clevenger-type apparatus	GC-MS	1.5%	10.3390/molecules27227961
Pectis elongata Kunth	Brazil	Hydrodistillation using a Clevenger-type apparatus for 4 h	GC-MS	0.10%	10.1002/ffj.1546
Pelargonium geraniaceae	India	Hydrodistillation using a Clevenger-type apparatus	GC-MS	1.50%	10.1002/%28SICI%291099-1026%28200003/04%2915:2%3C105::AID-FFJ875%3E3.0.CO;2-G
Perovskia abrotanoides Karel.	Iran	Hydrodistillation using a Clevenger-type apparatus	GC-MS	6.40%	10.1002/ffj.1508
Perovskia atriplicifolia Benth	Iran	Hydrodistillation using a Clevenger-type apparatus	GC-MS	8.0% (arial plant parts)	10.1021/jf0341619
	Iran	Steam distillation	GC-MS	6.39% (flower), 9.36% (leaf), 9.55% (stem)	10.1002/ffj.988
Perovskia atriplicifolia Benth	Pakistan	Hydro-distillation in a Clevenger-type apparatus for 5 h	GC-MS	5.70%	10.1002/%28SICI%291099-1026%28199901/02%2914:1%3C38::AID-FFJ778%3E3.0.CO;2-8
Petroselinum crispum	Mauritius	NR	GC-MS	Nil	10.1002/cbdv.202000921

Supplementary material

Phellodendron amurenserupr.	Poland	Hydrodistillation	GC-MS	0.60% (unripe fruit)<br>0.40% (ripe fruit)<br>0.40% (air-dried ripe fruit)<br>0.40% (leaves)<br>0.30% (flowers)	10.1002/ffj.1349<br>Lis
Phlomis chorassanica Bunge. (Lamiaceae)	Iran	Hydrodistillation using a Clevenger-type apparatus	GC-MS	3.3% (aerial plant parts)	10.1002/ffj.1338
Phlomis cretica	Greece	Hydrodistillation using a Clevenger-type apparatus for 3 h	GC-MS	2.20%	10.1002/ffj.1717
Phlomis ferruginea Ten.	Italy	Hydrodistillation using a Clevenger-type apparatus for 3 h	GC-MS	4.10%	10.1002/ffj.1740
Phlomis olivieri Benth	Iran	Steam distillation	GC-MS	2.70%	10.1002/ffj.1156
Phlomis persica Boiss	Iran	Hydrodistillation using a Clevenger-type apparatus	GC-MS	1.4% (aerial plant parts)	10.1002/ffj.1338
Phoenix dactylifera L.	Saudi Arabia	Hydrodistillation using a Clevenger-type apparatus for 4 to 5 h	GC-MS, GC-FID	0.40%	10.1016/j.actatropica.2013.08.003
Pilocarpus pennatifolius Lemmaire (Rutaceae)	Brazil	Hydrodistillation using a Clevenger-type apparatus	GC-MS	0.1% (leaves)	10.1002/ffj.1306
Pimpinella anisum	Greece	Steam distillation for 4 h in a modified Clevenger distillation apparatus	GC-MS	nil	10.1007/s00436-012-3097-1<br>KIMBARIS
	Poland	Hydrodistillation using a Clevenger-type apparatus	GC-MS, counter-current chromatography	0.19%	10.1002/jssc.201300407
Pinus attenuata Lemmon	Greece	Hydrodistillation using a Clevenger-type apparatus	GC-MS	3.50%	10.1002/ffj.990
Pinus heldreichii Christ	Greece	Hydrodistillation using a Clevenger-type apparatus	GC-MS	1.00%	10.1002/ffj.990
Pinus mugo Turra	Serbia	Hydrodistillation using a Clevenger-type apparatus	GC-MS	0.40%	10.1002/ffj.1390
Pinus peuce Griseb	Greece	Hydrodistillation using a Clevenger-type apparatus	GC-MS	0.90%	10.1002/ffj.990
Pinus pinaster Ait.	Greece	Hydrodistillation using a Clevenger-type apparatus	GC-MS	14.80%	10.1002/ffj.990
	France	Hydrodistillation using a Clevenger-type apparatus	GC-MS	2.20%	10.1002/ffj.1865
Pinus radiata D. Don	Greece	Hydrodistillation using a Clevenger-type apparatus	GC-MS	Trace <0.05%	10.1002/ffj.990
Piper aduncum	Panama, Bolivia	Hydrodistillation using a Clevenger-type apparatus	GC-MS, GC-FID	1.9% (Panama), no trace (Bolivia)	10.1002/ffj.1369
	Brazil	Hydrodistillation using a Clevenger-type apparatus	GC-MS, GC-FID	4.1% (leaves)	https://dx.doi.org/10.1590/S0102-695X2013000500005
Piper cernuum		Computer aided detection (SISTEMAT system)	13C NMR spectroscopy	1.74%	10.1016/S0003-2670(01)01204-1

Supplementary material

Piper cubeba	India	NR	GC-MS	0.19%	10.1007/s00436-011-2695-7
Piper fridrichsthalii	Panama, Costa Rica	Hydrodistillation using a Clevenger-type apparatus	GC-MS, GC-FID	0.3% (Costa Rica), 1.4% (Panama)	10.1002/ffj.1181
Piper gaudichaudianum	Brazil	Hydrodistillation using a Clevenger-type apparatus	GC-MS, GC-FID	16.50%	https://dx.doi.org/10.1016/j.fct.2009.06.035
	Brazil	Hydrodistillation using a Clevenger-type apparatus	GC-MS, GC-FID	16.50%	10.1016/j.fct.2013.03.013
Piper nigrum		Extraction with methanol and extraction with water reflux distillation	Capillary electrochromatography	0.70%	10.1002/jssc.200600456
Piper pseudoliindenii	Costa Rica	Hydrodistillation using a Clevenger-type apparatus	GC-MS, GC-FID	7.00%	10.1002/ffj.1181
Piper regnellii		Computer aided detection (SISTEMAT system)	13C NMR spectroscopy	0.40%	10.1016/S0003-2670(01)01204-1
Pittosporum senacia subsp. senacia	Mauritius	NR	GC-MS	0.30%	10.1002/cbdv.202000921
Pittosporum tobira	Lisbon, Portugal	Hydrodistillation using a Clevenger-type apparatus	GC-MS	0.3% (leaves), 1.0% (fruit, capsules), 0.2% (flower)	10.1002/ffj.1798
Platycladus orientalis L.		Hydrodistillation using a Clevenger-type apparatus	GC-MS	0.40%	10.1111/j.1365-2184.2008.00561.x
	China	Soaked in sodium chloride solution and distilled by electric heating	Headspace solid-phase microextraction combined with GC-MS	7.34–14.41%	https://doi.org/10.3390/molecules28052043
Plectranthus amboinicus (Lour.) Spreng	India	Hydrodistillation using a Clevenger-type apparatus	GC-MS	9.67%	https://dx.doi.org/10.1007/s00436-010-1996-6
Plectranthus barbatus	India	Hydro-distillation of in a Clevenger apparatus for 8 h	GC-MS	1.62%	10.1007/s00436-015-4809-0
Plectranthus grandis	Brazil	Steam distillation using a Clevenger apparatus for 2 h	GC-MS	2.5 – 3.8%	10.1002/ffj.1730
Plectranthus ornatus	Brazil	Steam distillation using a Clevenger apparatus for 2 h	GC-MS	2.9 – 3.3%	10.1002/ffj.1730
Plinia cauliflora	Brazil	Hydrodistillation using a Clevenger-type apparatus for 5 h	GC-MS, GC-FID	nil	10.1002/ffj.1638
Plinia cordifolia	Brazil	Hydrodistillation using a Clevenger-type apparatus for 5 h	GC-MS, GC-FID	1.80%	10.1002/ffj.1638
Plinia edulis	Brazil	Hydrodistillation using a Clevenger-type apparatus for 5 h	GC-MS, GC-FID	2.60%	10.1002/ffj.1638
Plinia trunciflora	Brazil	Hydrodistillation using a Clevenger-type apparatus for 5 h	GC-MS, GC-FID	0.90%	10.1002/ffj.1638

Supplementary material

Polygonum hydropiper L	Singapore	Dynamic headspace sampling, simultaneous distillation and extraction, and liquid–liquid extraction with dichloromethane (D)	GC-MS	1.3% (dynamic headspace sampling) 0.9% (liquid extraction)	10.1002/ffj.1363
Prangos asperula Boiss.		Hydrodistillation using a Clevenger-type apparatus	GC-MS	0.30%	10.1111/j.1365-2184.2008.00561.x
Psidium acutangulum	Brazil	Hydrodistillation using a Clevenger-type apparatus for 4 h	GC-MS	4.90%	10.1002/ffj.1219
Psidium guajava	Brazil	Hydrodistillation using a Clevenger-type apparatus for 4 h	GC-MS	1.10%	10.1002/ffj.1219
Psidium guineense	Brazil	Hydrodistillation using a Clevenger-type apparatus for 4 h	GC-MS	Nil	10.1002/ffj.1219
Psidium striatulum	Brazil	Hydrodistillation using a Clevenger-type apparatus for 4 h	GC-MS	2.80%	10.1002/ffj.1219
Pterodon pubescensm	Turkey	Stainless-steel tank with mechanical stirring using dichloromethane as liquid extractor	GC-MS, GC-FID	0.64%	10.1016/j.biopha.2019.108693
Pulicaria mauritanica Coss. (Asteraceae)	Algeria	Hydrodistillation using a Clevenger-type apparatus	GC-MS, GC-FID, C-NMR	GC-MS trace <0.05% C-NMR 0.4%	https://doi.org/10.1002/ffj.3223
Ravensara aromatica Sonnerat	Madagascar	Hydrodistillation using a Clevenger-type apparatus for 4 h	GC-MS	0 - 0.1%	10.1002/ffj.1735
Rhabdosciadium microcalycinum Hand.-Mazz	Turkey	Hydrodistillation using a Clevenger-type apparatus for 3 h	GC-MS	0.20%	10.1002/ffj.1639
Rhabdosciadium oligocarpum (Post ex Boiss.) Hedge et Lamond	Turkey	Hydrodistillation using a Clevenger-type apparatus for 3 h	GC-MS	0.20%	10.1002/ffj.1639
Rosmarinus officinalis var. troglodytorum	Tunisia	Hydrodistillation using a Clevenger-type apparatus	GC-MS	0.38%	10.1016/j.fct.2010.08.010
Rosmarinus officinalis var. typicus	Tunisia	Hydrodistillation using a Clevenger-type apparatus	GC-MS	0.78%	
Rosmarinus officinalis	Algeria	Steam distillation	GC-MS	0.4% (steam distillation), nil (hydrodistillation using a Clevenger-type apparatus)	10.1002/ffj.1226
	Messina, Sicily	MAHD- milestone dry dist microwave reactor	GC-MS, GC-FID	0.78%	10.1002/jssc.200400037
Saccharomyces cerevisiae (with engineered mevalonate pathway	Germany	Ethyl acetate extraction	GC-MS	12.5-22.5mg/l	https://doi.org/10.1016/j.ymben.2022.10.004
Saccocalyx satureioides Coss et Durieu	Algeria	Hydrodistillation using a Clevenger-type apparatus for 3 h	GC-MS	0.30%	10.1002/ffj.1661

Supplementary material

Salvia amplexicaulis	Lithuania	Simultaneous distillation/extraction in a Likens–Nickerson apparatus and supercritical fluid extraction with CO_2	GC-MS, GC-FID	6.9 mg/kg	10.1002/ffj.3389
Salvia argentea L.	Serbia	Hydrodistillation using a Clevenger-type apparatus for 3 h	GC-MS	10.70%	10.1002/ffj.989
Salvia austriaca	Lithuania	simultaneous distillation/extraction in a Likens–Nickerson apparatus and supercritical fluid extraction with CO_2	GC-MS, GC-FID	1.3 mg/kg	10.1002/ffj.3389
Salvia brachyodon	Belgrade	Hydrodistillation using a Clevenger-type apparatus	GC-MS	10.80%	10.1002/ffj.1132
Salvia canariensis	Gran Canaria	Hydrodistillation using a Clevenger-type apparatus	GC-MS	1.1% (after flowering) 1.6% (before) 0.8% (during)	Https://onlinelibrary.wiley.com/doi/10.1002/ffj.1504
Salvia chionantha Boiss	Turkey	Hydrodistillation using a Clevenger-type apparatus	GC-MS	4.82%	10.1016/j.jchromb.2006.11.044
Salvia dumetorum	Lithuania	Simultaneous distillation/extraction in a Likens–Nickerson apparatus and supercritical fluid extraction with CO_2	GC-MS, GC-FID	1.6 mg/kg	10.1002/ffj.3389
Salvia forsskaolei	Lithuania	Simultaneous distillation/extraction in a Likens–Nickerson apparatus and supercritical fluid extraction with CO_2	GC-MS, GC-FID	23.5 mg/kg	10.1002/ffj.3389
Salvia fruticosa	Israel	Steam distillation for 1 h	GC-MS	3.90%	10.1021/jf901162f
Salvia glutinosa	Lithuania	Simultaneous distillation/extraction in a Likens–Nickerson apparatus and supercritical fluid extraction with CO_2	GC-MS, GC-FID	30.2 mg/kg	10.1002/ffj.3389
Salvia Glutinosa L	Serbia	Hydrodistillation	GC-MS	4.20%	10.1002/ffj.1291
Salvia guaranitica	Brazil	Hydrodistillation using a Clevenger-type apparatus for 2 h	GC-MS	1.02-3.32%	10.1002/ffj.1817
Salvia nemorosa	Lithuania	Simultaneous distillation/extraction in a Likens–Nickerson apparatus and supercritical fluid extraction with CO_2	GC-MS, GC-FID	2.3 mg/kg	10.1002/ffj.3389
Salvia nemorosa	Serbia	Hydrodistillation	GC-MS	1.90%	10.1002/ffj.1291
Salvia officinalis	Tunisia (Sfax town)	Hydrodistillation using a Clevenger-	GC-MS, GC-FID	4.60%	10.1016/j.biopha.2018.09.108

		type apparatus for 2 h			
	Tunisia (Kelibia)	Hydrodistillation using a Clevenger-type apparatus for 3 h	GC-MS	8.94%	10.1021/jf901877x
	Lithuania	Simultaneous distillation/extraction in a Likens–Nickerson apparatus and supercritical fluid extraction with CO_2	GC-MS, GC-FID	2057.9 mg/kg	10.1002/ffj.3389
	Hungary	Steam distillation using a Clevenger-type apparatus for 3 h	GC-MS, GC-FID	15.1% (*Salvia officinalis* l), 33.24% (*Salvia officinalis* cv. ‘purpurascens), 23.38% (*Salvia officinalis* cv. ‘tricolor’), 14.55% (*Salvia officinalis* cv. ‘kew gold’), 8.52% (*Salvia judaica* boiss)	10.1021/jf9005092
	Tunisa	Hydrodistillation using a Clevenger-type apparatus for 4 h	GC-MS	4.37%	10.1016/j.fct.2009.08.005
	Serbia, Montenegro	Hydrodistillation using n-hexane	GC-MS, GC-FID	3.35-12.49%	10.1002/ffj.1065
	Portugal	Macerated in 10 ml of pentane	GC-MS	7.46% (leaves), 5.23% (stem), 4.31% (flowers)	https://doi.org/10.1021/jf001102b
	Portugal	Hydrodistillation using a Clevenger-type apparatus	GC-MS	6.80%	https://doi.org/10.1021/jf020945v
Salvia officinalis × *Salvia fruticosa,* cv. Newe Ya'ar No. 4	Israel	Hydrodistillation using a Clevenger-type apparatus for 1.5 h	GC-MS	5.19% (stem), 3.17% (mature leaves), 4.96% (young leaves), 6.59% (leaf primordia in main branch), 6.34% (leaf primordia in secondary branches), 6.34% (leaf primordia in secondary branches), 3.42% (upper shoots), 3.86% (lower shoots)	10.1021/jf9901587
Salvia pratensis	Lithuania	Simultaneous distillation/extraction in a Likens–Nickerson apparatus and supercritical fluid extraction with CO_2	GC-MS, GC-FID	11.6 mg/kg	10.1002/ffj.3389
Salvia przewalskiimaxim	Tibet	Hydrodistillation for 3 h, using a Clevenger-type apparatus	GC-MS	0.21% (leaves) 3.64% (flowers)	10.1002/ffj.1607
Salvia reflexa Hornem	Serbia	Hydrodistillation	GC-MS	Nil	10.1002/ffj.1291
Salvia santoliniifolia	Iran	Hydrodistillation using a Clevenger-type apparatus	GC-MS	7.80%	10.1002/%28SICI%291099-1026%28199903/04%2914:2%3C77::AID-

Supplementary material

					FFJ726%3E3.0.CO;2-9
Salvia sclarea	Greece	Hydrodistillation using a Clevenger-type apparatus	GC-MS	<0.05%	10.1021/jf020422n
	Lithuania	simultaneous distillation/extraction in a Likens–Nickerson apparatus and supercritical fluid extraction with CO_2	GC-MS, GC-FID	Nil	10.1002/ffj.3389
	Uruguay	Steam distillation for 2 h at normal atmospheric pressure	GC-MS	0.40%	10.1002/ffj.1282
Salvia verticillata	Lithuania	simultaneous distillation/extraction in a Likens–Nickerson apparatus and supercritical fluid extraction with CO_2	GC-MS, GC-FID	11.6 mg/kg	10.1002/ffj.3389
	Iran	Hydrodistillation using a Clevenger-type apparatus	GC-MS	Nil	10.1002/%28SICI%291099-1026%28199903/04%2914:2%3C77::AID-FFJ726%3E3.0.CO;2-9
Sambucus ebulus	Iran	Hydrodistillation using a Clevenger-type apparatus for 4 h	GC-MS	Nil (control), 5.41% (treated with indole-3-acetic acid), 1.85% (treated with naphthalene acetic acid)	10.4314/tjpr.v13i4.13
Santolina chamaecyparissus	India	Hydrodistillation	Triplicate distillations	0.6% (jammu), 2.3% (srinagar) 2.5% (tissue culture raised foliage)	10.1002/ffj.1440
Satureja spicigera C. Koch Boiss.	Iran	Hydrodistilled using a Clevenger-type apparatus for 4 h	Dried over anhydrous sodium sulphate	0.2%.	10.1002/ffj.1642
Satureja macrantha C. A. Mey	Iran	Hydrodistilled using a Clevenger-type apparatus for 4 h	Dried over anhydrous sodium sulphate	0.2%.	10.1002/ffj.1642
Scaligeria tripartita	Turkey	Hydrodistillation using a Clevenger-type apparatus	GC-MS	0.2% (fruit)	10.1016/j.jchromb.2006.11.041
Schinus mole	Sardinia	CO_2-based extraction; hydrodistilled using a Clevenger-type apparatus for 4 h	GC-MS	0.4% (CO_2-based extraction) 0.2% (hydrodistilled)	10.1002/ffj.1350
Schinus polygamus (Cav.) Cabrera f. Chubutensis	Argentina	Hydrodistilled in a Clevenger-type apparatus	GC-MS	0.80%	Https://onlinelibrary.wiley.com/doi/10.1002/ffj.1270
Scleria hirtella	Brazil	Hydrodistillation for 4 h using a Clevenger apparatus.	GC-MS	0.10%	10.1002/ffj.1593
Senecio nutans Sch.-Bip.	Peru	Hydrodistillation using a Clevenger-type apparatus for 3 h	GC-MS	Nil	10.1002/ffj.1204

Supplementary material

Senecio selloi Spreng. DC.	Brazil	Hydrodistillation using a Clevenger-type apparatus	GC-MS	0.6% (aerial plant parts)	10.1590/S1516-05722013000400005
Sephredium brevifolium	Skardu Baltistan, Pakistan	Hydrodistillation using a Clevenger-type apparatus	GC-MS	3%	10.1016/j.bjp.2019.04.013
Seseli andronakii Woron.	Athens	Hydrodistillation using a Clevenger-type apparatus	GC-MS	no trace <0.1%	10.1002/ffj.1572
Seseli petraeum M. Bieb.	Athens	Hydrodistillation using a Clevenger-type apparatus	GC-MS	1.00%	10.1002/ffj.1572
Seseli tortuosum	Italy	Hydrodistillation using a Clevenger-type apparatus for 2 h	GC-MS	0.30%	10.1002/ffj.1154
Silphium perfoliatum	Poland	Steam distillation method in Deryng's apparatus	GC-MS	1.4% (leaf oil) 0.6% (inflorescence oil) 2.9% (rhizome oil)	10.1002/ffj.1418
Solanum tuberosum	Bonin, Japan	Hydrodistillation using a Clevenger-type apparatus	GC-MS	41.2 ng/cm2	10.1021/jf040437g
Sphaeranthus africans	Vietnam	Hydrodistillation using a Clevenger-type apparatus	GC-MS	0.4%	10.3390/molecules27227961
Spreng (Verbenaceae)	Tanzania	Hydrodistillation using a Clevenger-type apparatus	GC-MS	1.40%	https://dx.doi.org/10.1002/ffj.3625
Spruce *Picea orientalis* (L.) Link	Belgrade	Boiled in water then mixed with petroleum benzine for distillation	GC-MS	1.02% (wood extract), 0.18% (needle extract)	10.1002/ffj.1196
Stachys alpina ssp. Dinarica	Bosnia and Herzegovina	Hydrodistillation in a Clevenger-type apparatus	GC-MS	2.80%	10.1002/ffj.1684
Stachys sylvatica L.	Italy	Hydrodistillation using a Clevenger-type apparatus	GC-MS	0.1% (inflorescence), 0.6% (leaves)	10.1002/ffj.1308
Styrax japonicus	China	Static headspace solid-phase microextraction	GC-MS	1.57%	10.1002/ffj.3654
Syzygium aromaticum	Madagascar	Clove oil purchased	GC-MS	0.5% (Madagascar) 1.80% (Indian)	10.1080/10611860500422958
	India	NR	GC-MS	3.78%	10.1016/j.jbiosc.2016.09.011
	Iran	NR	GC-MS	1.73%	10.1002/ffj.3595
Syzygium aromaticum (Eugenia caryophyllata)	Italy	Commercial and steam-distilled clove oil	HPLC	1.10 ±0.02 g/100ml	10.1002/jssc.200600023
Syzygium coriaceum	Mauritius	NR	GC-MS	0.70%	10.1002/cbdv.202000921
Syzygium jambos (L.) Alston, (Myrtaceae)- rose apple	Brazil	Hydrodistillation using a Clevenger-type apparatus	GC-MS	7.07% (leaves)	https://dx.doi.org/10.1590/S0102-695X2013005000035
Syzygium samarangense	Mauritius	NR	GC-MS	0.30%	10.1002/cbdv.202000921
Syzygium zeylanicum (Myrtaceae)	India	Hydrodistillation 8 h Clevenger apparatus, dried with anhydrous NaSO4	GC-MS	37.80%	10.1007/s00436-016-5025-2

Taiwania cryptomerioides	Taiwan	Hydrodistillation using a Clevenger-type apparatus	GC-MS, GC-FID	0.30%	10.1002/ffj.1685
Tetrataenium lasiopetalum	Iran	Hydrodistillation using a Clevenger-type apparatus	GC-MS, GC-FID	0.4% (aerial plant parts)	10.1002/ffj.1767
Teucrium scordium	Sicily	Hydrodistillation 3 h	Dried over anhydrous sodium sulphate	0.50%	Https://www.tandfonline.com/doi/full/10.1080/14786419.2019.1709193
Teucrium fruticans	Sicily and Malta	Hydrodistillation 3 h	Dried over anhydrous sodium sulphate	5.6% (Sicily) 3.3%, (Malta)	Https://www.tandfonline.com/doi/full/10.1080/14786419.2019.1709193
Teucrium libanitis	Spain	Hydrodistillation using a Clevenger-type apparatus for 2.5 h	GC-MS	nil	10.1002/ffj.1256
Teucrium royleanum	Pakistan	Hydrodistillation using a Clevenger-type apparatus	GC-MS	0.60%	10.1002/ffj.1774
Teucrium siculum	Sicily	Hydrodistillation 3 h	Dried over anhydrous sodium sulphate	8.60%	Https://www.tandfonline.com/doi/full/10.1080/14786419.2019.1709193
Teucrium turredanum	Spain	Hydrodistillation using a Clevenger-type apparatus for 2.5 h	GC-MS	4.7–10.1%	10.1002/ffj.1256
Thymbra capitata	NR	Water distillation in a Clevenger-type apparatus	GC-MS	0.10%	10.1007/s00436-010-1800-7
Thymbra spicata L	Turkey	Homogenised plant item was extracted with 250 ml extraction solvent (methanol) for 24 hours	GC-MS	nil	10.1002/ffj.3636
Thymus cilicicus	Turkey	Homogenised plant item was extracted with 250 ml extraction solvent (methanol) for 24 hours	GC-MS	0.10%	10.1002/ffj.3636
Thymus citriodorus	Italy	Steam distillation	GC-MS	nil	10.1016/j.resmic.2016.11.004
Thymus vulgaris	Italy	Steam distillation	GC-MS	0.10%	10.1016/j.resmic.2016.11.004
Thymus *Zygis sylvestris*		Water distillation in a Clevenger-type apparatus	GC-MS	Trace	10.1007/s00436-010-1800-7
Tilapia (*Oreochromis niloticus*)	Bangladesh	Dynamic headspace sampling method	GC-MS	115 ng/g	10.1021/acs.jafc.7b00497
Triumfetta rhomboideajacq	Burkina Faso	Hydrodistillation with a Clevenger-type apparatus for 2 h	GC-MS	4.90%	10.1002/ffj.1511 Mevy
Turnera diffusa Willd. var.afrodisiaca(Ward) Urb.	Brazil	Hydrodistillation using a Clevenger-type apparatus for 4 h	GC-MS	0.20%	10.1002/ffj.1155
Turnera subulata Sm.	Brazil	Hydrodistillation using a Clevenger-type apparatus for 3 h	GC-MS	1.30%	10.1590/1983-084X/13_011

Supplementary material

Unonopsis guatterioides	French Guyana	Steam distilled 3 h	Filtered over anhydrous sodium sulphate	2.5% (root)<br>6.3% (fruit)	10.1002/%28SICI%291099-1026%28199703%2912:2%3C95::AID-FFJ611%3E3.0.CO;2-Z
Valeriana officinalis	United States	3 h of hydrodistillation using a Clevenger type distillation apparatus	GC-MS	0.68% (select cultivar)<br>8.46% (Anthose cultivar)	10.1021/jf0353990
Varronia curassavica	Brazil	Hydrodistillation using a Clevenger-type apparatus	GC-MS, GC-FID	1.36% (plant subject to 20% light-full sun), 1.24% (plant subject to 50% light-full sun), 1.14% (plant subject to 70% light-full sun), 1.58% (plant subject to 100% light-full sun)	10.1016/j.bjp.2014.10.005
Vernonia brasiliana (L.) Druce	Brazil	Hydrodistillation	GC-MS	8.85%	10.1016/j.biopha.2020.111025
Washington-navel-type oranges	Turkey	Peel oil extracted by simple distillation	GC-MS	0.11%	10.1002/ffj.3576
Xylopia rubescens Oliv.	Côte d'Ivoire	Hydrodistillation using a Clevenger-type apparatus	GC-MS	0.80%	10.1002/ffj.3155
Ylang-ylang	Comoro Islands	NR	GC-MS, GC-FID	20.9 mg/ml	https://dx.doi.org/10.1002/ffj.3625
	Madagascar	NR	GC-MS, GC-FID	39.9 mg/ml	https://dx.doi.org/10.1002/ffj.3625
Zanthoxylum avicennae (Lam.) DC. (Rutaceae)	China	Hydrodistillation using a modified Clevenger-type apparatus for 6 h	GC-MS	0.07%	10.4314/tjpr.v13i3.13
Zanthoxylum bungeanum	China	Molecularly imprinted solid-phase extraction	GC-MS	1.11%	10.1002/jssc.201701014
Zanthoxylum rhetsa seeds	India	Hydrodistillation using a Clevenger-type apparatus	GC-MS	Trace <0.1%	10.1002/ffj.1598
Zataria multiflora	Iran	Commercial	GC-MS	0.13%	10.1016/j.ijbiomac.2018.12.085
Zataria multiflora Boiss.	Iran	Hydrodistillation using a Clevenger-type apparatus	GC-MS	0.19%	10.1016/j.fct.2010.03.025
Zingiber nimmonii	India	Hydro-distillation in a Clevenger apparatus for 8 h	GC-MS	19.60%	10.1007/s00436-016-4920-x Govindarajan
Zingiber zerumbet	Malaysia	Root dried at 60°C for 24 h. Dried root underwent Soxhlet extraction	HPLC	60-15,800 µg/g (plant grown in a variety of growth regulators and elicitors)	10.3390/molecules27154744
Ziziphora clinopodioides	Turkey	Homogenised plant item was extracted with 250 ml extraction solvent	GC-MS	Nil	10.1002/ffj.3636

		(methanol) for 24 hours			

GC-FID – gas chromatography–flame ionisation detection; GC-MS – gas chromatography–mass spectrometry; HPLC – high-performance liquid chromatography; NMR – nuclear magnetic resonance; NR – not recorded
